# VqLecRKV.4 and VqBAK1 Modulate Grapevine Resistance to Powdery Mildew by Regulating Dynamic Balance of ROS

**DOI:** 10.1111/pbi.70595

**Published:** 2026-02-18

**Authors:** Yajuan Li, Ruilin Li, Zhuoyu Liu, Kewei You, Rongxin Li, Chen Jiao, Zhenjiang Wei, Zhi Li, Yijie Zhao, Xiping Wang

**Affiliations:** ^1^ State Key Laboratory for Crop Stress Resistance and High‐Efficiency Production, College of Horticulture Northwest A&F University Yangling Shaanxi China; ^2^ Key Laboratory of Horticultural Plant Biology and Germplasm Innovation in Northwest China, Ministry of Agriculture Northwest A&F University Yangling Shaanxi China; ^3^ Key Laboratory of Molecular Biology of Crop Pathogens and Insects, Institute of Biotechnology Zhejiang University Hangzhou China

**Keywords:** grapevine, powdery mildew, ROS dynamic balance, TurboID‐mediated proximity labelling, VqBAK1, VqLecRKV.4

## Abstract

Grapevine powdery mildew, caused by the fungal pathogen *Erysiphe necator*, severely impacts plant growth and berry quality. However, the grapevine receptors and molecular mechanisms underlying grapevine resistance to *E. necator* remain poorly understood. In this study, we identify a G‐type Lectin receptor‐like kinase (LecRK), *VqLecRKV.4*, identified from the wild Chinese grapevine *Vitis quinquangularis*, whose expression is significantly upregulated in response to *E. necator* infection. Overexpression of *VqLecRKV.4* in the susceptible cultivar *V. vinifera* ‘Thompson Seedless’ confers enhanced resistance against the pathogen, as evidenced by significantly reduced fungal colonisation and sporulation. Through TurboID‐mediated proximity labelling, we demonstrate that VqLecRKV.4 interacts with VqCu/ZnSOD1. Further analysis reveals that VqLecRKV.4 promotes ROS accumulation and cell death by upregulating *VqCu/ZnSOD1* expression, thereby inhibiting *E. necator* colonisation. A critical challenge in plant immunity is balancing immune responses to avoid overactivation. Here, we discover that VqBAK1 interacts with VqLecRKV.4 and attenuates its overreaction. Collectively, our findings reveal that the VqLecRKV.4‐VqBAK1 module fine‐tunes grapevine resistance to powdery mildew by maintaining ROS homeostasis, providing novel insights into the molecular mechanisms of grapevine immunity and its regulation to prevent detrimental overreaction.

## 1 Introduction

Grapevines hold significant socio‐economic importance worldwide, with the global cultivation area reaching approximately 7.2 million hectares in 2023 (https://www.oiv.int/). The most widely cultivated grapevine variety is the European grapevine ( *Vitis vinifera* L.), highly susceptible to powdery mildew (PM) caused by *Erysiphe necator* (Cadle‐Davidson et al. 2011; Gadoury et al. 2012). In contrast, many wild Chinese *Vitis* species, such as *V. quinquangularis*, exhibit strong resistance to this pathogen (Gao et al. 2016). This natural variation provides an invaluable genetic resource for identifying key resistance genes (Hu et al. 2021). In this study, we leveraged this advantage by focusing on the wild Chinese grapevine *V. quinquangularis* clone ‘Shang‐24’ as a potential source of resistance genes. Our objective was to identify and characterise PM‐resistance genes from this wild species for potential incorporation into *V. vinifera* cultivars to enhance their immunity against powdery mildew.

In response to pathogens, plants utilise receptors to detect pathogen‐associated molecular patterns (PAMPs), thereby activating pattern‐triggered immunity (Boller and Felix 2009; Yu et al. 2017). These receptors, which primarily belong to the receptor‐like kinase (RLK) family, play a central role in initiating immune responses (Macho and Zipfel 2014; Wu and Zhou 2013). Structurally, RLK proteins comprise an extracellular domain, a single transmembrane domain, and a cytoplasmic kinase domain, the latter facilitating signal transduction to activate plant defence mechanisms (Shiu and Bleecker 2001; Wang et al. 2008). The lectin receptor‐like kinase (LecRK) subfamily represents one of the largest and most well‐characterised RLKs, further subdivided into three categories based on their extracellular domains: G, C, and L types. G‐type LecRKs, characterised by a conserved extracellular B‐lectin domain, are particularly known for their ability to recognise a broad range of pathogens, making them an important factor in plant immune responses (Vaid et al. 2013, 2012; Yang et al. 2016).

Studies have revealed that G‐type LecRKs play conserved roles in immune signalling by regulating reactive oxygen species (ROS) accumulation, programmed cell death (PCD) and mitogen‐activated protein kinase (MAPK) cascades (Labbé et al. 2019; Mustamin et al. 2023; Tobias et al. 1992; Trontin et al. 2014; Walker and Zhang 1990; Wang et al. 2021). For example, *OsLecRK* has been shown to enhance innate immunity (Cheng et al. 2013), and *OsSDS2*, *OsPWL1* and *AtG‐LecRK‐I.2* regulate ROS signalling to activate immune responses (Chien, Chang, and Ting 2024; Fan et al. 2018; Xu et al. 2023). Similarly, *OsPID2* contributes to resistance against blast fungus by regulating plant cell death and innate immunity (Wang, Li, et al. 2023). Despite these findings, the precise function and downstream signalling mechanisms of G‐type LecRKs in grapevine disease resistance remain poorly understood.

While immune activation is crucial for pathogen defence, excessive immune responses can be detrimental to plant growth and development. To ensure survival, plants have evolved intricate regulatory mechanisms to balance immunity and growth (Fisher et al. 2012; Saijo and Loo 2020). Plants activate basal immune responses by recognising the *flg22* peptide derived from bacterial flagellin. However, this immune activation inhibits plant growth. To achieve a balance between defence and growth, plants have developed multiple strategies to fine‐tune immune activation and prevent excessive responses. For example, the immunosuppressive subtilase A (IssA), secreted by rhizosphere bacteria, degrades immune‐activating peptides, thereby mitigating excessive *flg22‐*mediated signalling and supporting an appropriate balance between growth and defence (Eastman et al. 2024). Furthermore, plants regulate immune balance by modulating protein degradation pathways.

In rice, for instance, the E3 ubiquitin ligase system suppresses premature immune activation by degrading OsNPR1 (Choi et al. 2022). Similarly, AtPAM16 facilitates the import of certain immune‐negative regulatory factors into mitochondria, protecting plant cells from damage caused by uncontrolled immune responses, such as excessive production of ROS (Huang et al. 2013). Recent studies have demonstrated that LecRKs play a crucial role in balancing plant immunity and growth by modulating the intensity and duration of signalling pathways. For instance, P2K1, upon sensing extracellular ATP (eATP), activates the S‐acylation capacity of PATs to suppress its own overactivation, thereby maintaining equilibrium between immune responses and plant growth (Chen et al. 2021). This integrative role is particularly critical for balancing immune metabolism during periods when plants contend with the competing demands of growth and defence. However, the molecular mechanisms by which LecRKs achieve the fine‐tuning of immune activation and suppression remain largely unknown in grapevine.

In this study, we identified a G‐type LecRK in grapevine, *VqLecRKV*.4, and demonstrated its positive role in conferring resistance against *E. necator*. Our findings reveal that during *E. necator* infection, VqLecRKV.4 interacts with VqCu/ZnSOD1, increasing its expression, which promotes an ROS burst to trigger immune defence. Furthermore, we discovered that VqBAK1 interacts with VqLecRKV.4 and can suppress the overactivation triggered by VqLecRKV.4. This suppression occurs through the interaction between VqBAK1 and VqCAT2, which reduces ROS levels. Our findings highlight how grapevine fine‐tunes its defence response through the VqLecRKV.4‐VqBAK1 complex, regulating the dynamic balance of ROS, a process which is crucial for the grapevine's resistance to PM.

## 2 Results

### 2.1 VqLecRKV.4 Positively Regulates Grapevine Resistance to Powdery Mildew

**FIGURE 1:**
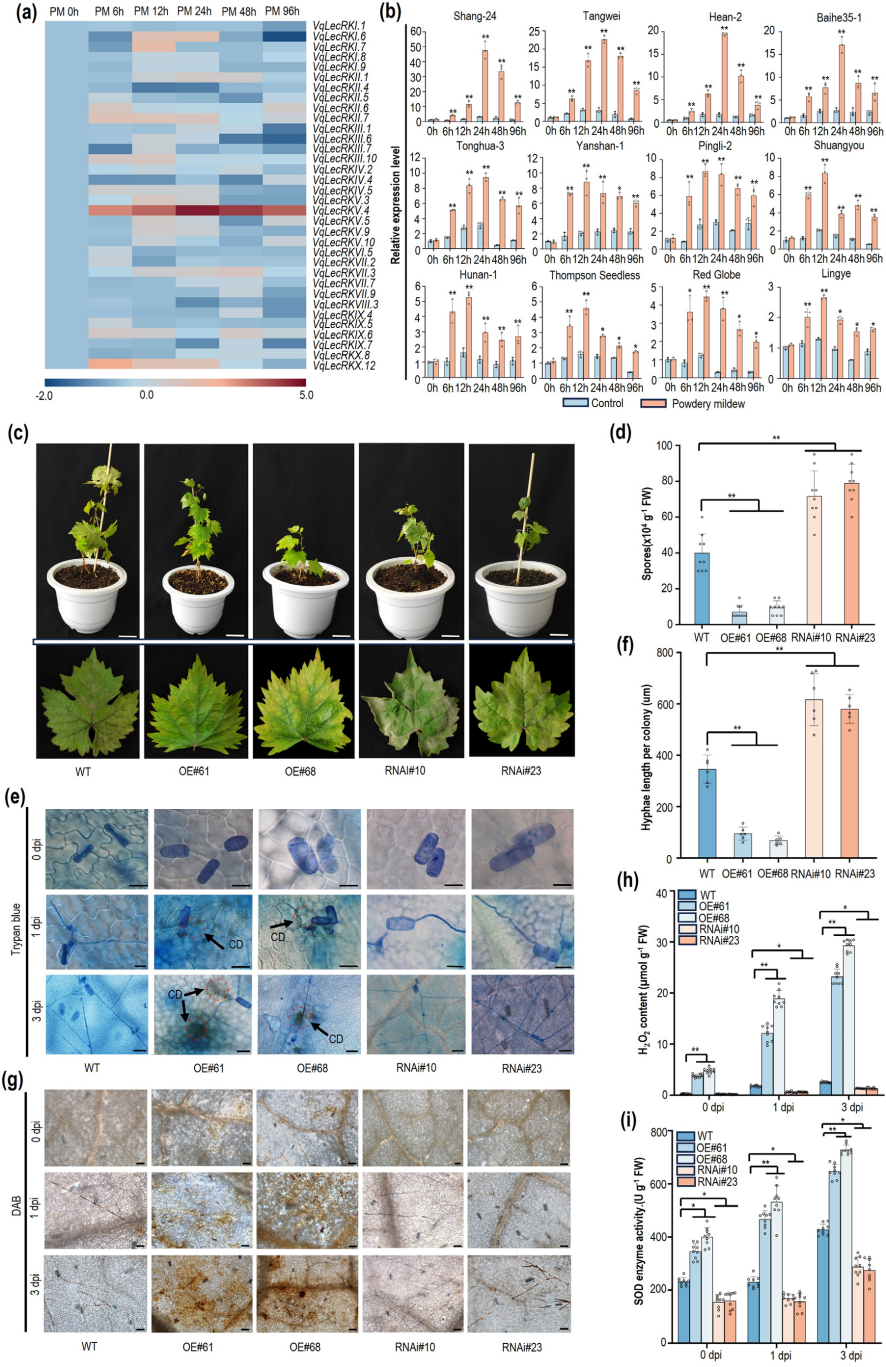
Overexpressing *VqLecRKV.4* enhances resistance to powdery mildew in *V. vinifera*. (a) Semiquantitative expression profiles of the G‐type *LecRK* genes in ‘Shang‐24’ leaves inoculated with *E. necator*. Red represents an increase in transcript abundance, whereas blue denotes a decrease. (b) Expression profiles of *VqLecRKV.4* gene in various grapevine cultivars. Values are the means ± SD of three biological replicates (*n* = 3). Asterisks indicate statistical significance using based on a t test (*p < 0.05, **p < 0.01). (c) The phenotype of wild type (WT), overexpressing *VqLecRKV.4* lines (OE61 and OE68) and RNAi‐*VqLecRKV.4* lines (RNAi10 and RNAi13) at 11 days' post inoculation (dpi) with *E. necator*. (d) Quantitative analysis of spore numbers per milligram fresh leaves at 11dpi with *E. necator*. Values are the means ± SD of three biological replicates, each with three technical replicates (*n* = 9). (e) Trypan blue‐stained observation of WT and transgenic *VqLecRKV.4* lines with *E. necator*; CD: Cell death; Scale bars = 20 μm. (f) Average hyphae length per colony of *E. necator* on plant leaves at 1 dpi. Values are the means ± SD of three biological replicates, each with two technical replicates (*n* = 6) (g) 3,3‐diaminobenzidine (DAB) staining of H_2_O_2_ production in *E. necator*‐infected WT and transgenic *VqLecRKV.4* lines. Scale bars = 50 μm. (h) H_2_O_2_ content of WT and transgenic *VqLecRKV.4* lines with *E. necator*. Values are the means ± SD of three biological replicates, each with three technical replicates (*n* = 9). (i) SOD enzyme activity in leaves of in *E. necator*‐infected WT and transgenic *VqLecRKV.4* lines. Values are the means ± SD of three biological replicates, each with three technical replicates (*n* = 9) (d, f, h, i) Asterisks indicate statistical significance using a Tukey's multiple comparison test followed by one‐way ANOVA (*p < 0.05, **p < 0.01).

LecRKs are a subset of RLKs characterised by extracellular lectin domains (Vaid et al. 2012; Yang et al. 2016). Previous transcriptome analysis of PM‐infected the resistant wild Chinese grapevine *V. quinquangularis* ‘Shang‐24’ identified 34 G‐type *LecRK* genes associated with disease resistance (Jiao et al. 2021). To phylogenetically classify these candidate genes within the broader *LecRK* family, we analysed the orthologous G‐type *LecRK* gene family in the *Vitis vinifera* reference genome (see Supporting Information). A total of 94 G‐type *LecRK* genes were identified in the *V. vinifera* genome (Figures S1–S3, Tables S2–S4, Supporting Information), underscoring the potential significance of this gene family in disease resistance. Expression analysis of these 34 G‐type *LecRK*s at multiple time points revealed that *VqLecRKV.4* consistently exhibited the highest transcript levels under *E. necator* inoculation (Figure 1a). Further investigation confirmed its importance, as the expression of *VqLecRKV.4* was significantly induced by *E. necator* across 12 grapevine varieties (Figure 1b). Protein sequence analysis of VqLecRKV.4 predicted a transmembrane domain (Figure S4, Supporting Information), and subcellular localisation experiment confirmed its presence on the cell membrane (Figure S5, Supporting Information). Transgenic *Arabidopsis* lines overexpressing *VqLecRKV.4* (OE‐*VqLecRKV.4*) exhibited enhanced resistance to PM, as evidenced by ROS production and localised cell death (Figure S6, Supporting Information).

To further investigate the function of *VqLecRKV.4*, we generated *VqLecRKV.4‐*overexpression (OE‐*VqLecRKV.4*) and RNA interference (RNAi‐*VqLecRKV.4*) lines through *Agrobacterium*‐mediated transformation of pro‐embryonic masses derived from *V. vinifera* L. cv. ‘Thompson Seedless’ (Figures S7 and S8, Supporting Information). Five independent OE grapevine lines (Figure 1 OE#61,OE#68; Figure S9 OE#1, OE#70 and OE#146) and five RNAi lines (Figure 1 RNAi#10, RNAi#23; Figure S9 RNAi#7, RNAi#16 and RNAi#18) were selected for *E. necator* inoculation (Supporting Information). At 11 days post inoculation (dpi), the OE‐*VqLecRKV.4* lines exhibited significantly reduced disease symptoms, with fewer lesions and lower spore concentrations, whereas the RNAi lines displayed more lesions and higher spore concentrations (Figure 1c,d, Figure S9a,b). Additionally, the overexpressing lines exhibited leaf yellowing and clear cell death at the infection sites, a phenomenon not observed in the wild type (WT) or RNAi lines (Figure 1c,e, Figure S9a,c). Consistently, at 1 dpi, hyphal length per colony was shorter in OE leaves but longer in RNAi leaves compared to WT (Figure 1f, Figure S9d). These observations suggest the occurrence of a hypersensitive response (HR), a defence mechanism that prevents further nutrient acquisition by pathogens in plant‐biotrophic pathogen interactions, as previously documented (Jones et al. 2016; Raff 1998). Moreover, the OE‐*VqLecRKV.4* lines accumulated higher levels H_2_O_2_ after *E. necator* inoculation, whereas WT and RNAi lines showed minimal accumulation (Figure 1f,g, Figure S9d,e). Consistent with these findings, the activity of SOD was significantly elevated in OE‐*VqLecRKV.4* lines but reduced in the RNAi lines compared to WT (Figure 1h,i, Figure S9f,g). The generation of reactive oxygen species (ROS) in plants is predominantly mediated by RBOH enzymes. Upon recognition of pathogen effector proteins by plant disease resistance proteins, EDS1 is recruited and activated, while the expression of PR5 can be induced by various signalling pathways. RT‐qPCR analysis revealed that in *VqLecRKV.4*‐overexpressing lines, the transcriptional levels of *VvrbohC2*, *VvEDS1* and *VvPR5* were all elevated compared to those in wild‐type and interference lines (Figure S10). These results collectively suggest that *VqLecRKV.4* is a critical positive regulator of grapevine resistance to PM, effectively limiting pathogen growth potentially through the modulation of ROS‐mediated defence mechanisms.

To further investigate the function of *VqLecRKV.4*, we generated *VqLecRKV.4‐*overexpression (OE‐*VqLecRKV.4*) and RNA interference (RNAi‐*VqLecRKV.4*) lines through *Agrobacterium*‐mediated transformation of pro‐embryonic masses derived from *V. vinifera* L. cv. ‘Thompson Seedless’ (Figures S7 and S8, Supporting Information). Five independent OE grapevine lines (Figure 1 OE#61,OE#68; Figure S9 OE#1, OE#70 and OE#146) and five RNAi lines (Figure 1 RNAi#10, RNAi#23; Figure S9 RNAi#7, RNAi#16 and RNAi#18) were selected for *E. necator* inoculation (Supporting Information). At 11 days post inoculation (dpi), the OE‐*VqLecRKV.4* lines exhibited significantly reduced disease symptoms, with fewer lesions and lower spore concentrations, whereas the RNAi lines displayed more lesions and higher spore concentrations (Figure 1c,d, Figure S9a,b). Additionally, the overexpressing lines exhibited leaf yellowing and clear cell death at the infection sites, a phenomenon not observed in the wild type (WT) or RNAi lines (Figure 1c,e, Figure S9a,c). Consistently, at 1 dpi, hyphal length per colony was shorter in OE leaves but longer in RNAi leaves compared to WT (Figure 1f, Figure S9d). These observations suggest the occurrence of a hypersensitive response (HR), a defence mechanism that prevents further nutrient acquisition by pathogens in plant‐biotrophic pathogen interactions, as previously documented (Jones et al. 2016; Raff 1998). Moreover, the OE‐*VqLecRKV.4* lines accumulated higher levels H_2_O_2_ after *E. necator* inoculation, whereas WT and RNAi lines showed minimal accumulation (Figure 1f,g, Figure S9d,e). Consistent with these findings, the activity of SOD was significantly elevated in OE‐*VqLecRKV.4* lines but reduced in the RNAi lines compared to WT (Figure 1h,i, Figure S9f,g). The generation of reactive oxygen species (ROS) in plants is predominantly mediated by RBOH enzymes. Upon recognition of pathogen effector proteins by plant disease resistance proteins, EDS1 is recruited and activated, while the expression of PR5 can be induced by various signalling pathways. RT‐qPCR analysis revealed that in *VqLecRKV.4*‐overexpressing lines, the transcriptional levels of *VvrbohC2*, *VvEDS1* and *VvPR5* were all elevated compared to those in wild‐type and interference lines (Figure S10). These results collectively suggest that *VqLecRKV.4* is a critical positive regulator of grapevine resistance to PM, effectively limiting pathogen growth potentially through the modulation of ROS‐mediated defence mechanisms.

### 2.2 VqLecRKV.4 Interacts With VqCu/ZnSOD1

**FIGURE 2:**
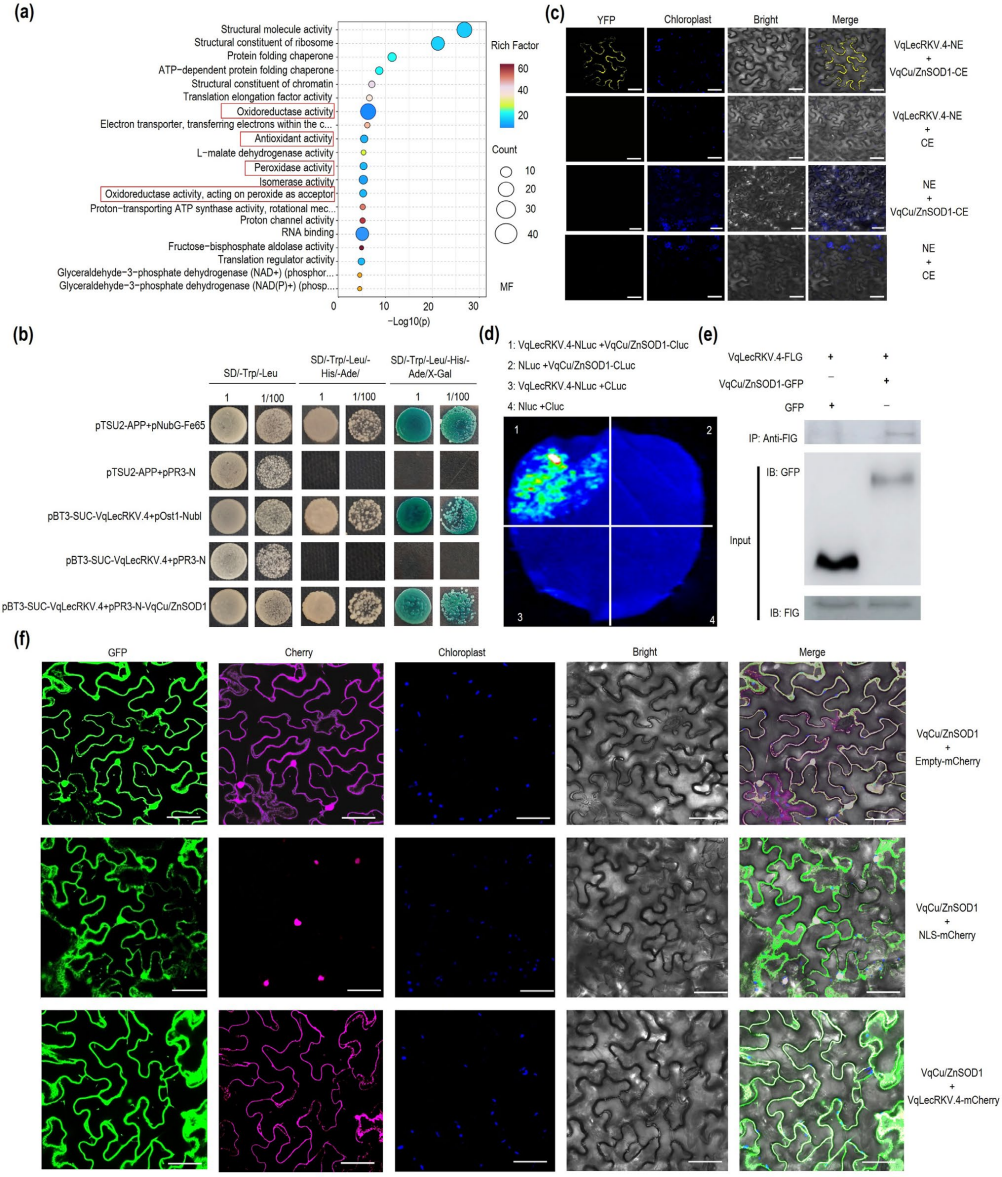
VqLecRKV.4 interacts with VqCu/ZnSOD1. (a) Enriched GO terms for proximal proteins of VqLecRKV.4 based on TurboID‐mediated labeling. The red box indicates the molecular function enrichment related to oxidoreductase activity. (b) Analysis of the interaction between VqLecRKV.4 and VqCu/ZnSOD1 through the DUAL membrane system. Co‐transformed NMY51 yeast cells were cultured on SD/−Trp‐Leu (DDO), SD/−Trp‐Leu‐His‐Ade (QDO) and QDO medium supplemented with X‐Gal. (c) BiFC assay for the interaction between VqLecRKV.4 and VqCu/ZnSOD1. From left to right: YFP, Chloroplast auto‐fluorescence, Bright field, and Merged field. Scale bars = 50 μm. (d) Verification of the interaction between VqLecRKV.4 and VqCu/ZnSOD1 by Split‐Luc assay. (e) Co‐IP assay for the interaction between VqLecRKV.4 and VqCu/ZnSOD1. Input: Western blot of VqLecRKV.4‐FLG, VqCu/ZnSOD1‐GFP, GFP; IP:FLG: Co‐IP of VqLecRKV.4‐FLG and VqCu/ZnSOD1‐GFP, VqLecRKV.4‐FLG/GFP; +: Contain; −: Free. (f) Subcellular localisation of VqCu/ZnSOD1. VqCu/ZnSOD1 was transiently co‐expressed with CHERRY or VqLecRKV.4‐cherry in the leaves of *N. benthamiana*. The combined images were constructed by green fluorescence channel (first panels), cherry fluorescence channel (second panels), chloroplast autofluorescence channel (third panels) and bright field image channel (fourth panels). Scale bar = 50 μm.

To investigate the mechanism by which *VqLecRKV.4* enhances resistance to PM, TurboID‐mediated proximity labelling was carried out to identify proteins proximal to VqLecRKV.4. Following the method described by (Chien, Reyes, et al. 2024), proteins were enriched with biotin. Mass spectrometry analysis identified 343 proteins in the *VqLecRKV.4* overexpressing group. Gene Ontology (GO) functional classification and statistical analysis revealed that a subset of oxidoreductase was significantly enriched (Figure 2a). Based on GO and KEGG pathway analysis, six candidate proteins were selected for further investigation (Figure 2a and Figure S11, Supporting Information). Subsequent screening using the DUAL membrane system demonstrated an interaction between VqLecRKV.4 and VqCu/ZnSOD1 in yeast cells (Figure 2b), while no interaction was observed with the other candidate proteins (Figure S12, Supporting Information). Further validation through BiFC, split‐LUC and CoIP assays confirmed the interaction between VqLecRKV.4 and VqCu/ZnSOD1 (Figure 2c–e). These findings demonstrate that VqLecRKV.4 interacts with VqCu/ZnSOD1 both in vitro and in planta.

Protein function is closely linked to its subcellular localisation. In our experiment, VqCu/ZnSOD1 was localised to both the cytoplasm and nucleus. However, when co‐expressed with VqLecRKV.4, both proteins were exclusively localised to the cytoplasm (Figure 2f), suggesting that VqLecRKV.4 may influence the localisation of the VqCu/ZnSOD1 protein through cellular interactions.

### 2.3 VqLecRKV.4 Regulates VqCu/ZnSOD1 to Enhance Grapevine Resistance

**FIGURE 3:**
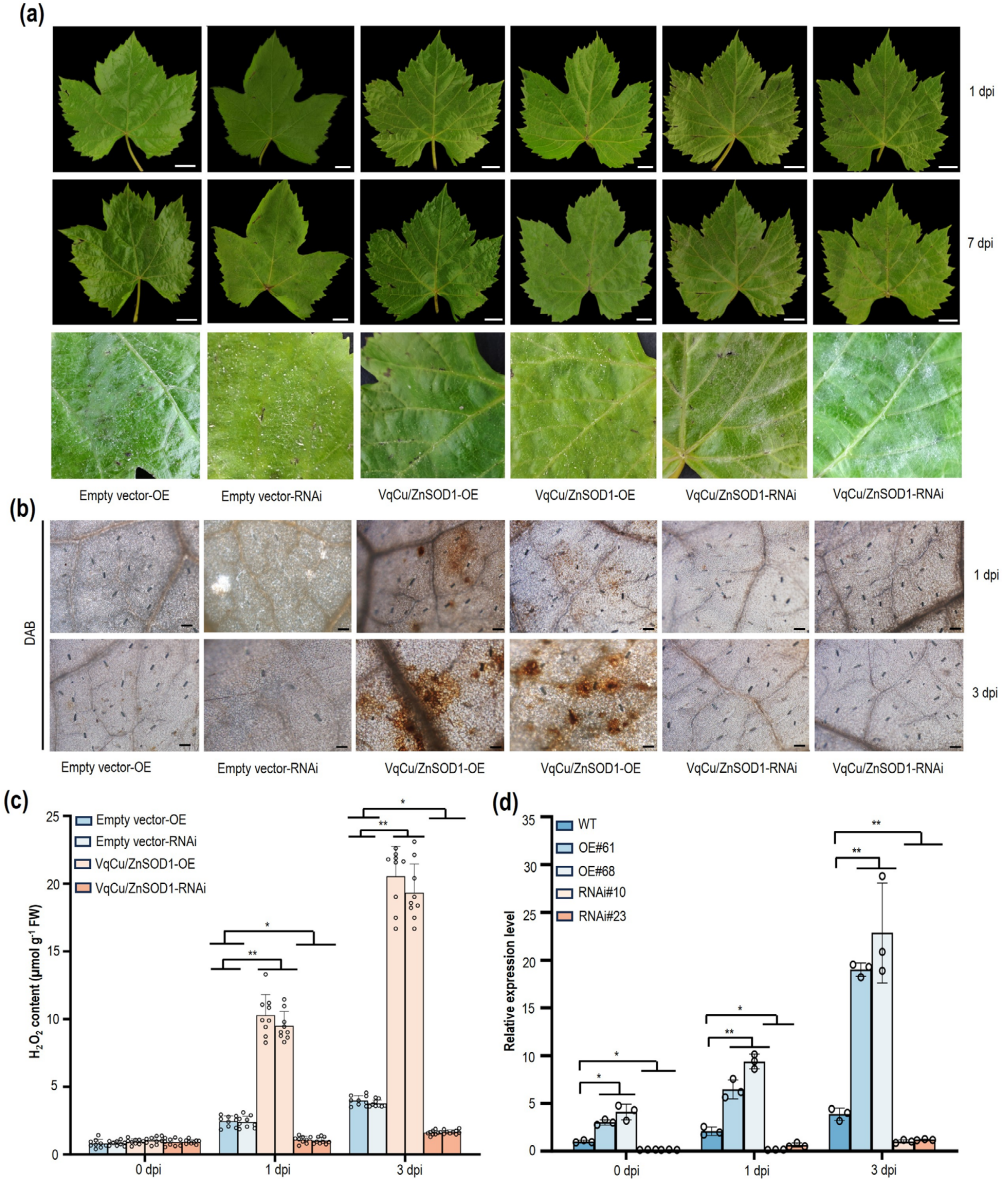
Functional analysis of *VqCu/ZnSOD1* in response to powdery mildew in *V. vinifera* . (a) The phenotype of Empty vector‐OE, Empty vector‐RNAi, OE‐*VqCu/ZnSOD1* and *VqCu/ZnSOD1‐*RNAi at 1 and 7 dpi with *E. necator*. Scale bars=1 cm. (b) 3,3‐diaminobenzidine (DAB) staining of H_2_O_2_ production in *E. necator*‐infected Empty vector‐OE, Empty vector‐RNAi, OE‐*VqCu/ZnSOD1* and *VqCu/ZnSOD1‐*RNAi leaves at 1 and 3 dpi. Scale bars = 50 μm. (c) H_2_O_2_ content of Empty vector‐OE, Empty vector‐RNAi, OE‐*VqCu/ZnSOD1* and *VqCu/ZnSOD1‐*RNAi leaves at 1 and 3 dpi with *E. necator*. Values are the means ± SD of three biological replicates, each with three technical replicates (*n* = 9). Asterisks indicate statistical significance using a Tukey's multiple comparison test followed by one‐way ANOVA (*p < 0.05, **p < 0.01). (d) The expression patterns of *VqCu/ZnSOD1* in the WT, OE61 and OE68 lines, RNAi10 and RNAi13 lines after inoculation with *E. necator* at different time points. Values are the means ± SD of three biological replicates (*n* = 3). Asterisks indicate significant different groups based on a t test (*p < 0.05, **p < 0.01).

SODs play critical roles in plant disease defence and have been shown to enhance resistance to PM in barley and rice (Li et al. 2019; Xu et al. 2014). VqCu/ZnSOD1, which encodes 152 amino acids with a predicted protein size of 15 kDa, belongs to the Superoxide dismutase [Cu‐Zn] family (Figure S13, Supporting Information) (Hu et al. 2019; Jiménez et al. 2024). To investigate how VqCu/ZnSOD1 regulates grapevine resistance to PM, we transiently overexpressed and RNA‐interfered *VqCu/ZnSOD1* in ‘Thompson Seedless’ leaves and measured gene expression at different time points post‐inoculation (Figure S14, Supporting Information). At 7 dpi, *VqCu/ZnSOD1‐*overexpressing leaves exhibited fewer fungal hyphae compared to empty vector (OE and RNAi) and RNAi leaves (Figure 3a). Additionally, H_2_O_2_ accumulation at 3 dpi was more pronounced in OE‐*VqCu/ZnSOD1* leaves, while RNAi‐*VqCu/ZnSOD1* leaves showed minimal accumulation (Figure 3b,c). The findings suggest that VqCu/ZnSOD1 positively regulates H_2_O_2_ levels, thereby enhancing grapevine defence against PM.

To further understand how *VqLecRKV.4* influences VqCu/ZnSOD1 expression, we examined the expression of *VqCu/ZnSOD1* at different time points following *E. necator* inoculation in *VqLecRKV.4* OE and RNAi lines. From 0 to 3 dpi, expression in the OE lines continuously increased and remained significantly higher than in the WT, while RNAi lines showed significantly lower expression (Figure 3d). These results suggest that, in response to *E. necator* infection, *VqLecRKV.4* enhances *VqCu/ZnSOD1* expression in grapevine, contributing to its resistance to PM.

### 2.4 Phosphorylation Event in the ROS Signalling Pathway Mediated by VqLecRKV.4

**FIGURE 4:**
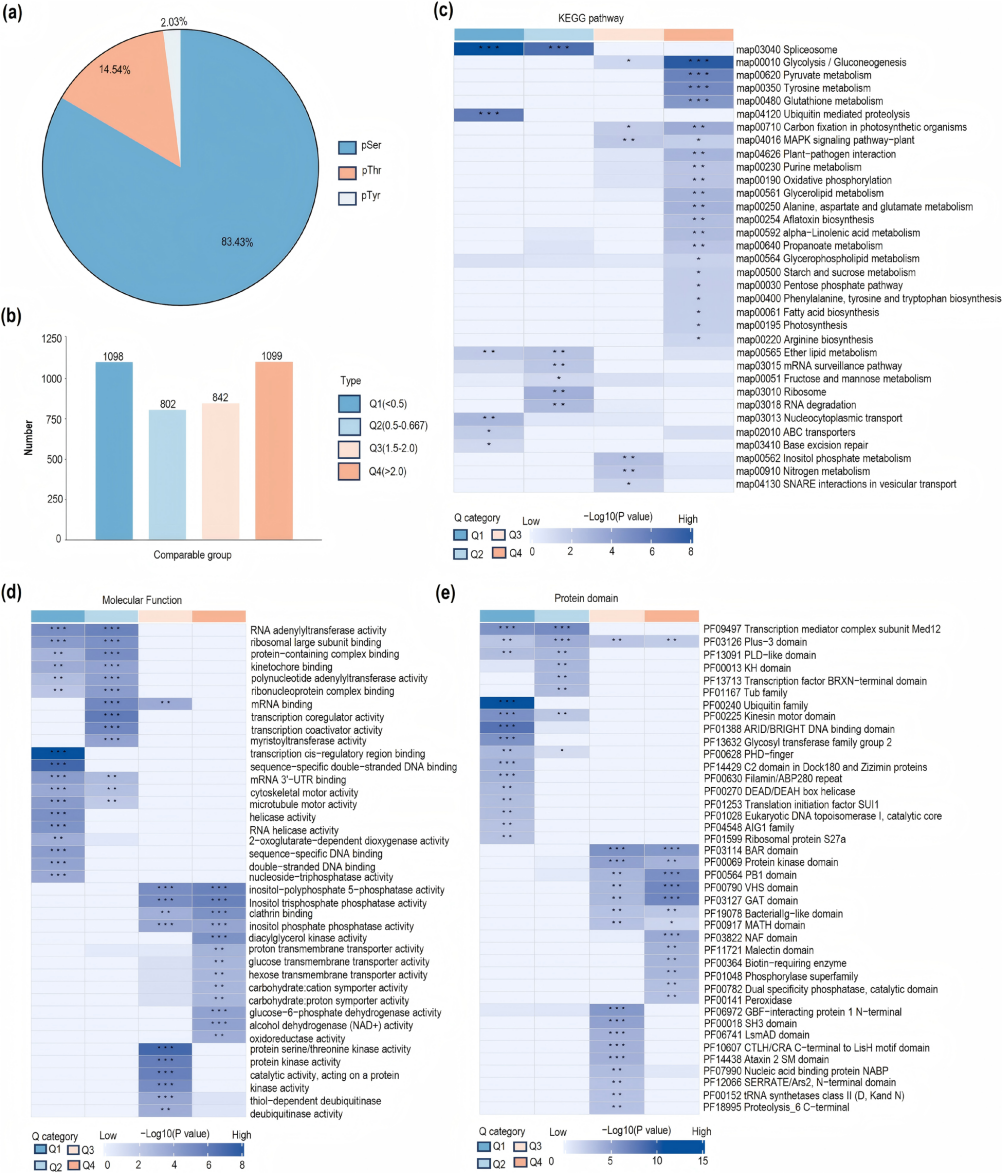
Enrichment analysis of VqLecRKV.4 phosphorylated proteins. (a) Frequency of phosphorylation at serine (S), threonine (T), and tyrosine (Y) residues in the 10 221 high confidence hits phosphopeptides. (b) Classification of proteins into four groups based on different fold changes, referred to as Q1 to Q4. (c, d, e) KEGG pathway analysis, molecular function analysis, and protein domains enrichment of Q1 to Q4 proteins in VqLecRKV.4 phosphorylated proteins.

RLKs recognise PAMPs in fungi and transmit signals through the phosphorylation of downstream substrates. In this study, mass spectrometry analysis using a DIA strategy was employed to investigate phosphorylated proteins in *VqLecRKV.4* overexpressing grapevine lines (Supporting Information). Six hours after *E. necator* inoculation, 10 221 peptides (corresponding to 6467 proteins) were detected with phosphorylation, of which 9308 had a phosphorylation likelihood exceeding 90% (Figure S15a,b, Supporting Information). Phosphorylation site analysis revealed that serine (83.43%) was the most frequently phosphorylated amino acid (Figure 4a). Among the 2455 phosphorylated proteins with altered expression, 1247 were upregulated and 1208 were downregulated (Figure S15c, Supporting Information). Subcellular localisation prediction indicated that these proteins were primarily located in the nucleus, chloroplasts, cytoplasm and cell membrane (Figure S15d, Supporting Information). Proteins were categorised into four groups based on differential expression levels, with a particular focus on the Q4 group (> 2.0) (Figure 4b).

ROS play a crucial role in plant disease resistance (Li et al. 2021; Liang et al. 2022; Wang et al. 2024). GO and KEGG analyses of differentially expressed post‐translationally modified proteins in the Q4 group revealed significant enrichment of molecular functions related to oxidoreductase activity, with the oxidative phosphorylation pathway also being notably enriched (Figure 4c,d). Protein domain analysis identified an enrichment of certain peroxidases, suggesting that VqLecRKV.4‐mediated resistance to PM in grapevine involves oxidoreductase activity (Figure 4e). Among these, 14 oxidoreductases were associated with the ROS pathway, including the previously identified protein VqCu/ZnSOD1, which showed increased phosphorylation levels (Table S6, Supporting Information). These findings suggest that following *E. necator* inoculation, grapevine activate VqLecRKV.4 to mediate intracellular ROS signalling through phosphorylation.

### 2.5 VqBAK1 Regulates H_2_O_2_ to Prevent VqLecRKV.4‐Induced Cell Death in Grapevines During *E. necator* Infection

**FIGURE 5:**
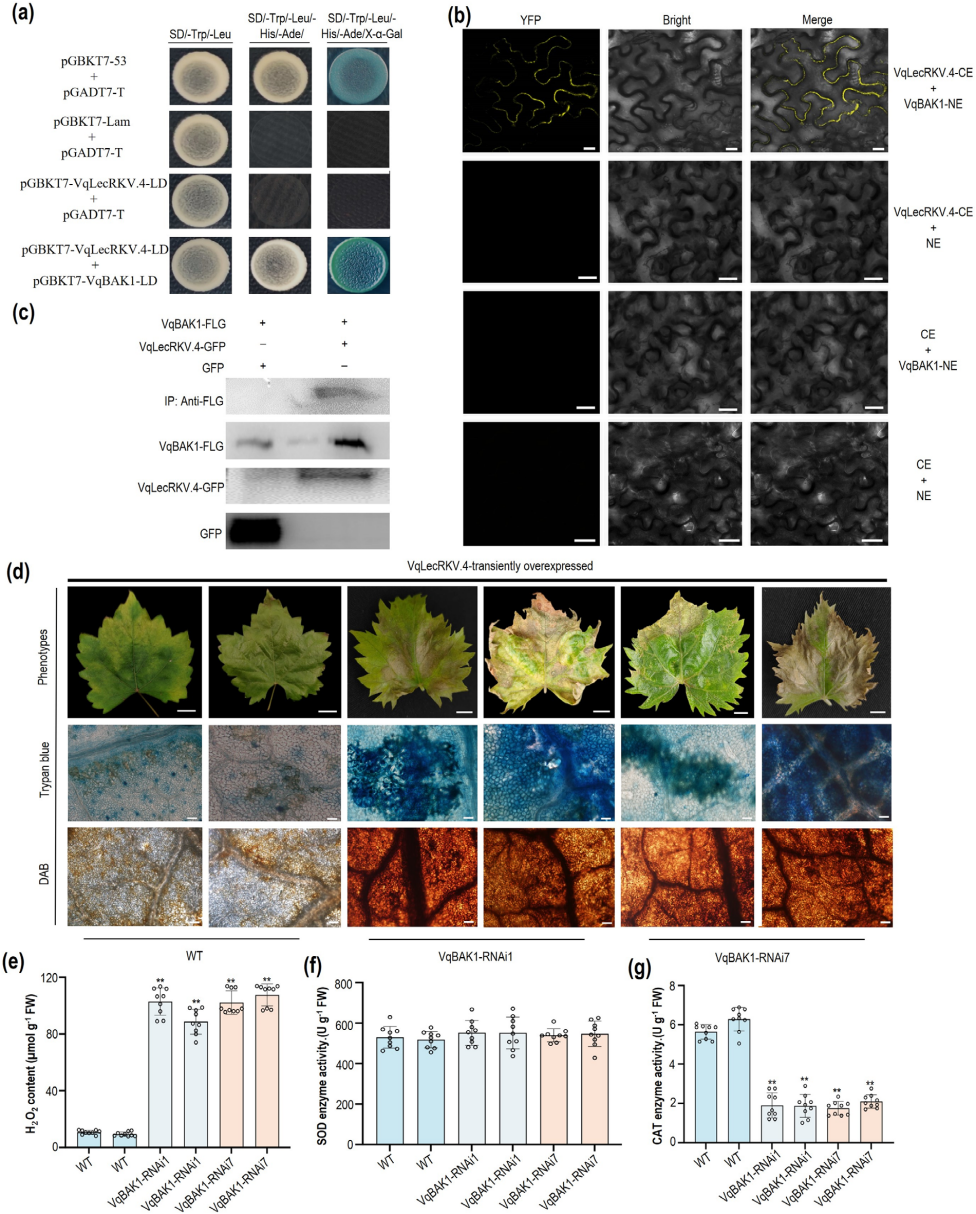
VqBAK1 controlled cell death in OE‐*VqLecRKV.4* grapevine induced by *E. necator* infection. (a) Interaction between the extracellular domain of the VqLecRKV.4 protein (VqLecRKV.4‐LD) and the extracellular domain of the VqBAK1 protein (VqBAK1‐LD) in yeast Y2H cells. (b) BiFC assay showing the interaction between VqLecRKV.4 and VqBAK1. From left to right: YFP fluorescence, Bright field, and merged field. Scale bars = 20 μm. (c) Co‐IP assay showing the interaction between VqLecRKV.4 and VqBAK1. Input: Western blot of VqBAK1‐FLG, VqLecRKV.4‐GFP and GFP; IP:FLG: Co‐IP of VqBAK1‐FLG and VqLecRKV.4‐GFP, GFP; +: Contain; −: Free. (d) The phenotype (Scale bars = 1 cm), trypan blue (Scale bars = 50 μm) and DAB (Scale bars = 50 μm) staining of leaves inoculated with *E. necator* at 7 dpi following transient transformation of *VqLecRKV.4* overexpression into WT and *VqBAK1* RNAi lines leaves. (e, f, g) Measurement of H_2_O_2_ content, SOD and CAT enzyme activity in leaves inoculated with *E. necator* at 7 dpi after transient transformation of *VqLecRKV.4* overexpression into WT leaves and *VqBAK1* RNAi lines leaves. Asterisks indicate a significant difference to *VqBAK1* overexpression lines. Values are the means ± SD of three biological replicates, each with three technical replicates (*n* = 9). Asterisks indicate statistical significance using a Tukey's multiple comparison test followed by one‐way ANOVA (*p < 0.05, **p < 0.01).

Plants face both biotic and abiotic stresses, and how they balance defence responses with normal growth to avoid overreaction is still under investigation. Overexpression of *VqLecRKV.4* triggers a ROS burst and induces the HR. However, cell death was not widely distributed at regions adjacent to the site of pathogen infection and the necrosis of leaf tissue was not observed (Figure 1c,e,f), suggesting that grapevines possess mechanisms to timely remove excessive H_2_O_2_ accumulation. Previous transcriptomic analysis identified another receptor‐like kinase, *VqBAK1*, which has been shown to regulate cell death (Jiao et al. 2015; Li et al. 2022). In this study, the interaction between VqLecRKV.4 and VqBAK1 was demonstrated using Y2H assays (Figure 5a) and further confirmed in plant tissues through BiFC and CoIP assays (Figure 5b,c).

To validate the function of *VqBAK1* in grapevine, RNA interference (RNAi‐*VqBAK1*) lines were generated (Figure S16a–c), and five independent RNAi lines (Figure 5 RNAi#1 and RNAi#7, Figure S17 RNAi#8, RNAi#14 and RNAi#15) were selected for further experiment. Transient expression of *VqLecRKV.4* in RNAi‐*VqBAK1* and WT lines showed yellowing and extensive necrosis in RNAi‐*VqBAK1* leaves (Figure 5d, Figure S17a). DAB and Trypan blue staining showed a significant burst of H_2_O_2_ and widespread cell death in RNAi‐*VqBAK1* leaves compared to WT leaves (Figure 5d,e, Figure S17a,b). Antioxidant enzyme activity assays indicated no change in SOD levels, while CAT activity was markedly reduced in RNAi‐*VqBAK1* leaves relative to WT leaves (Figure 5f,g, Figure S17c,d). These results suggest that *VqBAK1* regulates CAT activity to scavenge excessive H_2_O_2_ accumulation, thereby preventing cell death in grapevine leaves after inoculation with *E. necator*.

### 2.6 VqBAK1 Interacts With VqCAT2

**FIGURE 6:**
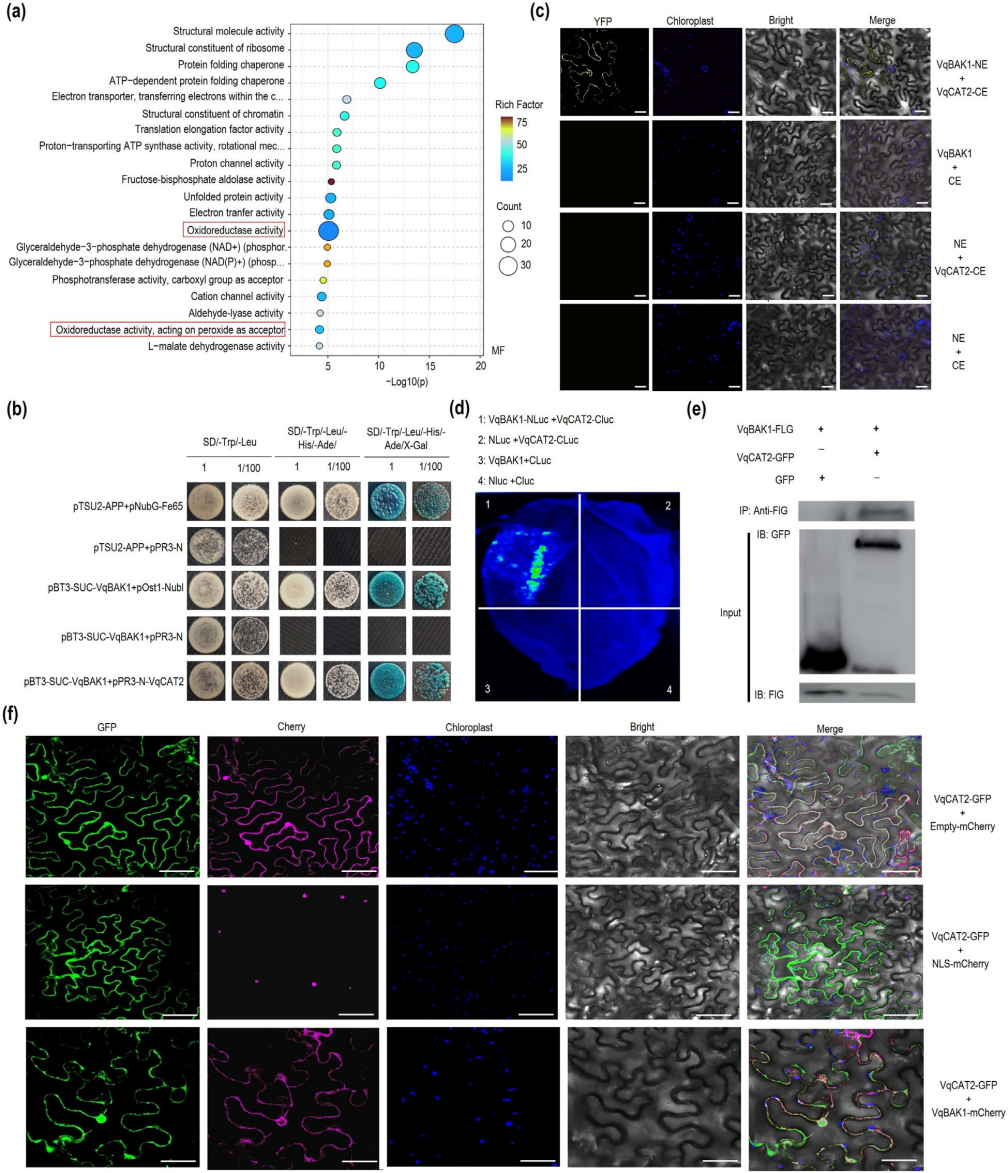
VqBAK1 interacts with VqCAT2. (a) Enriched GO terms for proximal proteins of VqBAK1 based on TurboID‐mediated labelling. The red box indicates the molecular function enrichment related to oxidoreductase activity. (b) Interaction between the VqBAK1 and VqCAT2 detected using the DUAL membrane system. NMY51 yeast co‐transformed with the relevant constructs were cultured on SD/−Trp‐Leu (DDO), SD/−Trp‐Leu‐His‐Ade (QDO) and QDO medium supplemented with X‐Gal. (c) BiFC assay for the interaction between VqBAK1 and VqCAT2. From left to right: YFP, Chloroplast auto‐fluorescence, Bright field, and merged field. Scale bars = 50 μm. (d) Verification of the VqBAK1 and VqCAT2 interaction with Split‐Luc. (e) Co‐IP assay for the interaction between VqBAK1 and VqCAT2. Input: Western blot of VqBAK1‐FLG、VqCAT2‐GFP、GFP; IP:FLG: Co‐IP of VqBAK1‐FLG and VqCAT2‐GFP, VqBAK1‐FLG/GFP; +: Contain; −: Free. (f) VqCAT2‐GFP was transiently co‐expressed with either CHERRY or VqBAK1‐cherry in *N. benthamiana* leaves. The combined images were constructed by green fluorescence channel (first panel), cherry fluorescence channel (second panel), chloroplast autofluorescence channel (third panel) and bright field image channel (fourth panel). Scale bars = 50 μm.

To further clarify the mechanism by which VqBAK1 controls cell death in grapevine, TurboID‐mediated proximity labelling was used to identify the interacting proteins. Based on these results, we selected the oxidoreductase CAT2 from the ROS pathway for further verification (Figure 6a). using the DUAL membrane system, an interaction between VqBAK1 with VqCAT2 was demonstrated in yeast cells (Figure 6b). This interaction was further validated by BiFC, split‐LUC, and Co‐IP assays (Figure 6c–e). These results confirm that VqBAK1 interacts with VqCAT2 both in vitro and in planta.

Additionally, we examined the co‐localisation of VqBAK1 and VqCAT2. The results indicated that VqCAT2 localised to both the cell membrane and the nucleus. When VqBAK1‐cherry was co‐expressed with VqCAT2‐GFP, co‐localisation was observed on both the cell and nuclear membranes (Figure 6f). These findings suggest that VqBAK1 may influence the functional activity of VqCAT2 through cellular interaction.

To investigate the function of *VqCAT2* in grapevine, we transiently overexpressed and RNA‐interfered *VqCAT2* in ‘Thompson Seedless’ leaves and measured its expression at various time points post‐inoculation (Figure S18). At 7 dpi, RNAi‐*VqCAT2* leaves exhibited necrosis compared to empty vector (OE and RNAi) and OE leaves (Figure S18a). Furthermore, H_2_O_2_ accumulation was more pronounced in RNAi‐*VqCAT2* leaves, while OE‐*VqCAT2* leaves showed minimal accumulation at 3 dpi (Figure S18b). We also examined the expression of *VqCAT2* in RNAi‐*VqBAK1* lines following *E. necator* inoculation. From 0 to 3 dpi, the expression of *VqCAT2* in the RNAi lines was continuously increased and remained significantly lower than in the WT (Figure S18c). The findings suggest that *VqCAT2* positively clears H_2_O_2_ levels after *E. necator* inoculation.

## 3 Discussion

G‐type LecRKs have been extensively identified and characterised in various plant species, including rice, *Arabidopsis*, and maize, where they play a crucial role in the plant disease resistance (Bao et al. 2023; Cheng et al. 2013; Hafeez et al. 2025; Kato et al. 2022; Li et al. 2024; Ranf et al. 2015; Zhou et al. 2023). However, research on G‐type LecRKs in grapevine resistance remains largely unexplored, with no systematic studies conducted to date. In this study, we present the first functional analysis of G‐type LecRKs in grapevine, demonstrating that silencing *VqLecRKV.4* significantly impairs grapevine resistance to PM, whereas its overexpression enhances resistance (Figure 1). These findings align with previous studies in other plant species. For example, the wheat intronless G‐type LecRK *Stb15* disease resistance gene has been confirmed to provide durable resistance against the leaf blotch pathogen *Zymoseptoria tritici* (Hafeez et al. 2025). Overexpression of *SDS2* in rice enhanced resistance to *Magnaporthe oryzae* (Fan et al. 2018), while knockout of the *OslecRK* in rice significantly suppressed downstream immune response genes, reducing resistance to *M. oryzae* and *Xanthomonas oryzae* (Cheng et al. 2013). Similarly, in *Arabidopsis*, mutations in the LORE compromised resistance to *Pseudomonas syringae* (Ranf et al. 2015), and loss‐of‐function mutants of the RDA2 showed reduced resistance to *Phytophthora infestans* (Kato et al. 2022). In maize, the *zmlecrk1* mutant displayed greater susceptible to *Pythium aphanidermatum* , *Bipolaris maydis* and *Rhizoctonia solani* (Li et al. 2024). Additionally, the *sbp1/sbp2* double mutants exhibited defects in defence gene expression and reduced resistance triggered by microbe‐associated molecular patterns (Bao et al. 2023). Interestingly, the role of G‐type LecRKs in plant immunity can vary, with some acting as negative regulators of resistance. For example, the *PWL1* in rice negatively regulated resistance to *Xanthomonas campestris* (Xu et al. 2023), and the *ERN1* gene mutant showed enhanced resistance to root‐knot nematodes (Zhou et al. 2023). In this study, we demonstrated that VqLecRKV.4 functions as a positive regulator of grapevine resistance to PM (Figure 1). These findings provide valuable genetic resources for grapevine disease‐resistance breeding and offer novel strategies and approaches for disease control in the grape industry.

ROS are important to plant immunity, driving programmed cell death (PCD) and the hypersensitive response (HR) to restrict pathogen spread, particularly for biotrophic pathogens (Balint‐Kurti 2019; Hofmann 2020; Lukan et al. 2023; McCombe et al. 2022; Rawat et al. 2023; Shu et al. 2024; Weralupitiya et al. 2024). G‐type LecRKs have been shown to regulate ROS accumulation and cell death, functioning as either negative or positive regulators. For instance, both *pwl1* and *ern1* mutants lead to increased H_2_O_2_ levels and cell death, indicating their role as negative regulators of ROS accumulation (Xu et al. 2023; Zhou et al. 2023). On the other hand, NbERK1 positively regulates the assembly of the chitin‐induced NbCERK1‐NbLYK4 complex, enhancing the transduction of chitin signalling and subsequently activating downstream immune responses, including the production of ROS (Lei et al. 2023). In rice, PID2 interacts with the E3 ubiquitin ligase OsPUB15, positively regulating both ROS accumulation and cell death (Wang, Li, et al. 2023). In this study, we demonstrated that *VqLecRKV.4* positively regulates grapevine resistance to powdery mildew by accumulating higher levels H_2_O_2_ (Figure 1). SOD plays a pivotal influence on enhancing disease resistance by modulating H_2_O_2_ levels in plant cells (Li et al. 2019; Zhang et al. 2024). Here, we confirmed the interaction between VqLecRKV.4 and ROS‐related enzymes VqCu/ZnSOD1 both in vitro and in planta, and upregulating VqCu/ZnSOD1 expression increases the accumulation of H_2_O_2_ after infection by *E. necator* in grapevine leaves (Figures 2 and 3). Moreover, we found that VqLecRKV.4 positively regulates resistance to PM in grapevine by increasing phosphorylation levels of ROS‐related enzymes, including VqCu/ZnSOD1 (Figure 4). Collectively, these results suggest that VqLecRKV.4 positively regulates ROS accumulation and the HR response by increasing phosphorylation levels of VqCu/ZnSOD1 in response to powdery mildew. However, the specific mechanisms by which VqLecRKV.4 phosphorylates VqCu/ZnSOD1 require further investigation. Interestingly, both overexpression (OE) and RNAi of *VqLecRKV.4* resulted in growth inhibition, as evidenced by reduced growth rates and smaller leaves compared to wild‐type plants (Figure S8e,g). The growth restriction in OE lines is likely attributable to sustained ROS accumulation and localised cell death (Czarnocka and Karpiński 2018; Huot et al. 2014). Conversely, growth impairment in RNAi lines may stem from attenuated basal ROS signalling and a disrupted redox balance essential for normal development (Foreman et al. 2003; Mittler 2017; Monshausen et al. 2007). perturbation of *LecRK* in other species yields similar pleiotropic effects on growth. For instance, overexpression of the rice *OsLecRK‐S.7* enhances disease resistance but significantly suppresses plant growth (Peng et al. 2025), while silencing of the wheat *TaLecRK‐IV.1* causes dwarfism without substantially compromising disease resistance (Saidou and Zhang 2022). Collectively, these findings indicate that precise regulation of VqLecRKV.4 activity is required to balance effective defence activation with normal growth, raising the question of how its activity is restrained to prevent excessive immune responses.

As research into G‐type LecRKs progresses, their interactions with BAK1 have gradually come to light, such as ZmLecRK1, AtG‐LecRK‐I.2 and AtSBP1/SBP2 (Bao et al. 2023; Chien, Chang, and Ting 2024; Li et al. 2024). In this study, we validated the interaction between VqLecRKV.4 and VqBAK1 in grapevine (Figure 5a–c). Moreover, BAK1, in coordination with other RLKs, can induce cell death. For instance, FLS2 forms a complex with BAK1 upon detecting flagellin, triggering downstream immune responses and ultimately leading to cell death (Chinchilla et al. 2007). Similarly, silencing ZmBAK1 significantly inhibits ZmLecRK1‐induced cell death (Bao et al. 2023). This mechanism has also been identified in other RLKs, such as EFR, PEPR1/PEPR2 and PsRLK6 (Chien, Chang, and Ting 2024; Han et al. 2024; Mühlenbeck et al. 2024; Pei et al. 2023; Zhang, Wang, et al. 2025). However, our study revealed that in VqBAK1‐RANi lines, overexpression of *VqLecRKV.4* resulted in widespread necrosis after pathogen infection in grapevine leaves (Figure 5d). This unexpected phenomenon highlights the complex role of BAK1 in plant immunity, particularly in driving cell death through intricate signalling pathways. For instance, silencing BAK1 can result in the hyperactivation of NLR proteins, which subsequently triggers cell death (Wu et al. 2020). In the *bak1/serk4* double mutant, the activation of the cysteine‐rich receptor‐like kinases (CRKs) is linked to cell death. Notably, the ectopic expression of CRK4 triggers STT3a/N‐glycosylation‐dependent cell death in both *A. thaliana* and *N. benthamiana* (de Oliveira et al. 2016). Additionally, in the absence of BAK1, the instability of Cyclic Nucleotide‐Gated Channel (CNGC) channels disrupts the intracellular environment, thereby activating necrosis signalling pathways (Yu et al. 2019).

The sustained activation of plant immune responses can lead to ROS over‐accumulation and the spread of cell death responses to regions adjacent to the site of pathogen infection (Castro et al. 2021; Chai et al. 2017; Czarnocka and Karpiński 2018; Lukan et al. 2020; Wang, Wei, et al. 2023). However, plants possess the ability to regulate their defence homeostasis to prevent the overactivation responses observed in animals. For instance, the NRG1C protein suppresses the activation of NRG1A/B by competitively binding to the EDS1‐SAG101 complex, thereby restricting HR at the site of pathogen infection (Huang et al. 2025). In *A. thaliana* , LSD1 curbs overactivation immune responses by modulating pathogen‐induced ROS metabolism and inhibiting the propagation of the EDS1‐PAD4‐ADR1 (EPA) defence signalling cascade (Lapin et al. 2020; Rustérucci et al. 2001). Similarly, OsROD1 regulates ROS signalling and suppresses EPA‐dependent autoimmunity to maintain immune homeostasis (Wu et al. 2024). Moreover, antioxidant enzymes serve as pivotal agents in ROS detoxification in plants (You et al. 2022; Zhang et al. 2016; Zou et al. 2015). Notably, BAK1 enhances the activity of these enzymes via specific signal transduction pathways, thereby bolstering the plant's antioxidant defence capacity (Fu et al. 2019; Zhang et al. 2020). In this study, we present the first functional analysis of *VqBAK1* in grapevine. We observed that in RNAi‐*VqBAK1* lines, overexpression of *VqLecRKV.4* led to excessive H_2_O_2_ accumulation in grapevine leaves after *E. necator* inoculation, while CAT activity was significantly diminished in the RNAi‐*VqBAK1* leaves (Figure 5d,e,g). Moreover, we confirmed that VqBAK1 interacts with VqCAT2 both in vitro and in planta (Figure 6). These results suggest that BAK1, by interacting with antioxidant enzymes, enhances their ability to scavenge H_2_O_2_ and prevent excessive cell death in *VqLecRKV.4* overexpressing grapevine leaves after inoculation. The bidirectional regulation of ROS by the VqLecRKV.4‐VqBAK1 module through VqCu/ZnSOD1 and VqCAT2 distinguishes it from previously reported LecRK‐BAK1 complexes in other plant species.

**FIGURE 7:**
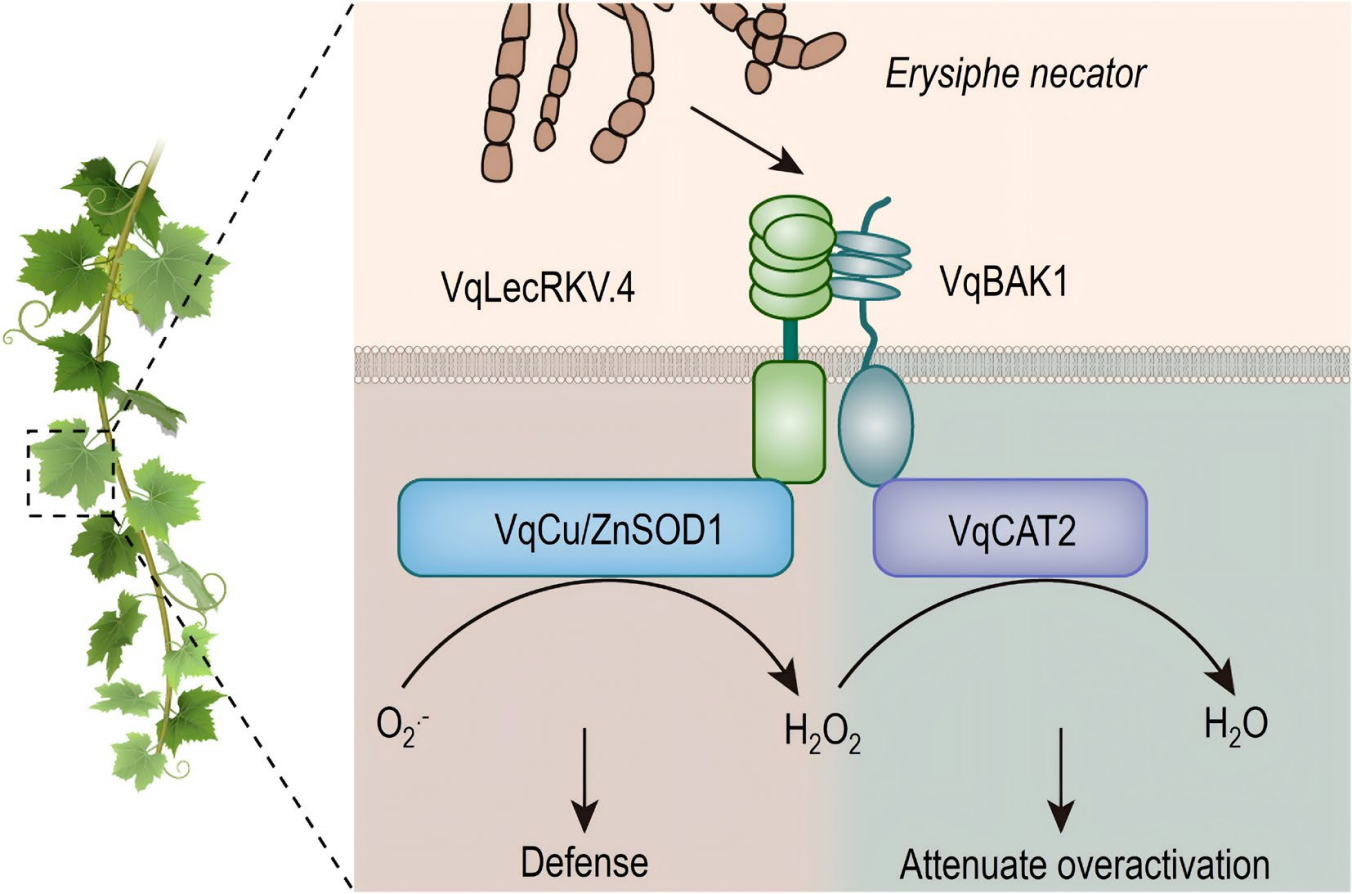
Model for VqLecRKV.4‐VqBAK1 model regulating grapevine resistance to powdery mildew (PM) by modulating ROS dynamic balance. VqLecRKV.4 induces increased VqCu/ZnSOD1 enzyme activity, leading to H_2_O_2_ accumulation, which causes cellular ROS accumulation and inhibits powdery mildew growth. Meanwhile, VqLecRKV.4 interacts with the co‐receptor VqBAK1, resulting in an increase in VqCAT2 enzyme activity to remove excess intracellular H_2_O_2_ to prevent over‐activation of immune responses in grapevine leaves.

In summary, we identified *VqLecRKV.4* as an important resistance gene against grapevine PM. It interacts with the VqBAK1 to form an immune complex that regulates grapevine resistance to PM. VqLecRKV.4 may positively enhance ROS accumulation and the HR by activating the activity of VqCu/ZnSOD1. Concurrently, VqBAK1 controls the excessive accumulation of H_2_O_2_ in overexpressed *VqLecRKV.4* grapevine leaves after inoculation, thereby preventing the spread of cell death responses to regions adjacent to the site of pathogen infection (Figure 7). This VqLecRKV.4‐VqBAK1‐Antioxidant enzyme module provides a more comprehensive understanding of the molecular mechanisms underlying grapevine resistance to PM. Additionally, it offers valuable strategies and genetic resources for developing grape varieties with enhanced disease resistance.

## 4 Materials and Methods

### 4.1 Plant Materials

The materials including grape (*Vitis quinquangularis* ‘Shang‐24’, *V. riparis* ‘Hean‐2’, *V. pseudoreticulata* ‘Baihe35‐1’, *V. davidii* ‘Tangwei’, *V. romanetii* ‘Pingli‐2’, *V. amurensis* ‘Tonghua’, *V. pseudoreticulata* ‘Hunan‐1’, *V. yeshanensis* ‘Yanshan‐1’, *V. hancockii* ‘Lingye’, *V. amurensis* ‘Shuangyou’, *V. vinifera* ‘Thompson Seedless’ and ‘Red Globe’) were cultivated in the grape germplasm resources orchard at the Northwest Agriculture and Forestry University in Yangling, Shaanxi, China. All transgenic grape materials, the *Arabidopsis thaliana* Columbia (Col‐0) and *Nicotiana benthamiana* were cultured at 20°C–21°C under conditions of 70% relative humidity in our laboratory. Grape powdery mildew (*Erysiphe necator*) and *Arabidopsis thaliana* powdery mildew (*Golovinomyces cichoracearum* UCSC1) were used in this study (Li et al. 2022; Wang, Wang, et al. 2023).

### 4.2 Generation of VqLecRKV.4 and VqBAK1 Transgenic Plants

The generation of transgenic grapevines was performed as previously described by Zhou et al. (2014) with modifications. Pro‐embryonic masses (PEMs) of *V. vinifera* L. cv. ‘Thompson Seedless’ were used as explants for *Agrobacterium tumefaciens* ‐mediated transformation. The full‐length coding sequences of *VqLecRKV.4* and *VqBAK1* were cloned into the pCAMBIA2300 vector (pCAMBIA2300‐GFP‐*VqLecRKV.4*, pCAMBIA2300‐GFP‐*VqBAK1*) to generate overexpression constructs. For RNAi constructs, non‐conserved sequences of 200‐300 bp each to construct pART27‐RNAi‐*VqLecRKV.4* and pART27‐RNAi‐*VqBAK1* vectors (see Table S1 for primers).

*Agrobacterium* strain GV3101 containing the fusion expression vectors was transformed into pro‐embryonic masses for 15 min, then co‐cultured on solid 1/2MS medium in darkness for 2 days. After co‐cultivation, PEMs were cultured on selection medium (4.43 g/L MS, 60 g/L sucrose, 3 g/L Phytagel, 1.5 g/L activated carbon, 200 mg/L Cef, 200 mg/L Carb, 75 mg/L Kan) and incubated under dark conditions at room temperature for 4–5 months, which promoted the emergence of new resistant embryos. Mature resistant embryos were transferred to X3 medium (4.43 g/L MS, 60 g/L sucrose, 3 g/L Phytagel, 1.5 g/L activated carbon, 1 mg/L IBA, 0.2 mg/L 6‐BA) for rooting, grown under light conditions for 4–8 weeks, and robust transgenic strains were transplanted into nursery seedlings (Zhang, Zhang, et al. 2025; Zhou et al. 2014).

DNA was extracted from the leaves of the transgenic plants to be tested and detection was carried out by agarose gel electrophoresis; preliminary identification of the transgenic situation was based on the results of electrophoresis. RNA extraction kits were used to extract RNA from grape leaves, which was then reverse‐transcribed to cDNA and quantitative primers were designed for quantitative experiments to evaluate the transgenic status of the grape plants by comparing with the expression level of wild‐type plants; the southern hybridisation was used to determine the copy number of transgenic lines, following the protocol provided with the Roche kit (11 585 614 910, Roche, Shanghai, China). Leaf proteins from the resistant lines were extracted, and SDS‐PAGE was formed using 10% resolving gel and 5% stacking gel, and PVDF membranes were used with a Bio‐Rad transfer unit. The fusion proteins were detected using an anti‐GFP antibody (Yisheng, Shanghai, China). The method of Clough and Bent (1998; Clough and Bent 1998) was referenced to acquire OE‐*VqLecRKV.4 Arabidopsis* lines, with detection methods similar to those used for grape transgenic lines.

### 4.3 TurboID‐Mediated Proximity Labelling

The 35 s promoter was cloned from the 2300‐GFP vector in our laboratory. *VqLecRKV.4* and *VqBAK1* were cloned from the cDNA of ‘Shang‐24’. The R4pGWB601–35 s‐*VqLecRKV.4*/*VqBAK1*‐TurboID‐YFP‐NES vector and the R4pGWB601–35‐TurboID‐YFP‐NES vector were constructed through multiplex PCR and transformed into *Agrobacterium* GV3101. Transient transformation was performed on ‘Shang‐24’ grape leaves using the method referenced by previously described (Wang, Wang, et al. 2023). After incubating in the dark for one day, the grape leaves were cut into 1 × 1 cm squares and placed in a 50 mL centrifuge tube. They were then treated with 50 μM biotin for over 120 min for biotin labelling. The leaves were then washed three times with pre‐chilled sterile water to stop labelling and remove excess biotin. The leaves were quickly dried and frozen in liquid nitrogen for total plant protein extraction, and the biotin‐labelled proteins were captured and purified using streptavidin magnetic beads, followed by liquid chromatography‐mass spectrometry (LC–MS) analysis (Chien, Reyes, et al. 2024). The mass spectrometry database search software used in this study was MaxQuant 2.4.14.0. All protein was annotated to the Gene Ontology (GO) identifiers using interproscan (version: 5.52–86.0) with default parameters. The ’topGO’ package was used to complete GO functional classification and statistics analysis.

### 4.4 Histochemical Staining

Genetically modified grape lines and wild‐type leaves were inoculated with grape PM, and leaves were selected for trypan blue and DAB (Solarbio, Beijing, China) staining and observed under a microscope (BX51, Tokyo, Japan) at 0, 1 and 3 days' post‐inoculation (dpi). The infected leaves of *Arabidopsis* were collected for trypan blue and DAB staining at 8 dpi after infection with *Golovinomyces cichoracearum* (UCSC1) infection.

### 4.5 Physiological Biochemical Index Measurement

The hydrogen peroxide (H_2_O_2_) content, Superoxide dismutase (SOD) activity and catalase (CAT) activity measurement in grape were used with a H_2_O_2_ assay kit (BC3590), SOD assay kit (BC1560) and CAT assay kit (BC0200, Solarbio, Beijing, China) following the manufacturer's instructions.

### 4.6 DUAL Membrane System

The DUAL membrane system is a yeast‐based screening assay designed to identify and characterise protein–protein interactions among integral membrane proteins, membrane‐associated proteins and soluble proteins in their natural environment. Based on the cDNA sequences of *VqLecRKV.4* and *VqBAK1*, after the removal of the signal peptide (SP) and the stop codon sequence, they were incorporated into the membrane yeast two‐hybrid bait vector pBT3‐SUC, creating the recombinant plasmid pBT3SUC‐*VqLecRKV.4*/*VqBAK1*. The candidate genes described in this study (*VqCu/ZnSOD1*, *VqCAT2*, etc.) are cloned from *V. quinquangularis* ‘Shang‐24’. Concurrently, candidate proteins (listed in Table S5, Supporting Information) were constructed into the pPR3‐N vector. Various control combinations were utilised: the pTSU2‐APP Control Vector and pNubG‐Fe65 Control Vector (positive control), the pPR3‐N Control Vector and pTSU2‐APP Control Vector (negative control), the pBT3‐SUC‐*VqLecRKV.4*/*VqBAK1* and pOst1‐Nubl Control Vector (functional test), the pBT3‐SUC‐*VqLecRKV.4*/*VqBAK1* and pPR3‐N Control Vector (autoactivation test), and the pBT3‐SUC‐*VqLecRKV.4*/*VqBAK1* with the pPR3N‐candidate protein plasmids. These were co‐transformed into the yeast strain NMY51. Positive clones were selected on SD/−Leu/−Trp (DDO) and SD/−Ade/‐His/−Leu/−Trp (QDO) media, and confirmed by blue colour development on QDO with 20 mg/L X‐Gal to validate interactions. The primers and vectors used are detailed in Table S1.

### 4.7 Y2H

Referencing the Yeastmaker Yeast Transformation System 2 User Manual, the extracellular ligand domain (LD) of VqLecRKV.4 was cloned into the pGBKT7 vector. Tests for yeast self‐activation and toxicity demonstrated that VqLecRKV.4‐LD‐BD lacked transcriptional self‐activation activity and was non‐toxic. The extracellular ligand domain (LD) of VqBAK1 was cloned into the pGADT7‐AD vector, and both VqLecRKV.4‐LD‐BD and VqBAK1‐LD‐AD were co‐transformed into Y2H Gold cells. The transformation products underwent serial dilution at a 10^−1^ gradient and were spotted on SD/−Ade/−His/−Leu/−Trp medium with 40 μg/mL X‐α‐Gal and 200 ng/mL AbA (‐QDO/X/A) to confirm interactions by the appearance of a blue colour. The pGADT7‐T+pGBKT7‐53 combination functioned as a positive control, while pGADT7‐T+pGBKT7‐Lam and co‐transformed empty vectors served as negative controls.

### 4.8 BiFC

The CDSs of *VqLecRKV.4* and *VqBAK1*, which lack stop codons and SP sequences, were cloned into the pSPYNE vector. Similarly, the CDSs of *VqCu/ZnSOD1* and *VqCAT2*, which also lack stop codons, were cloned into the pSPYCE vector. The fusion vectors were then separately co‐transformed into *N. benthamiana* as previously described. After incubating in the dark for 12 h, the leaves were cultured under normal conditions for an additional 2 days. The YFP fluorescence signals were detected using a confocal laser scanning microscope (LEICA TCS SP8, Germany) with 514 nm of excitation wavelength.

### 4.9 Split‐Luc

The CDSs of *VqLecRKV.4* and *VqBAK1*, which lack stop codons and SP sequences, were cloned into pCB1300‐Nluc, and the full‐length CDSs of *VqCu/ZnSOD1* and *VqCAT2*, which lack stop codons, were cloned into the pCB1300‐Cluc vector. The fusion vectors were then separately co‐transformed into *N. benthamiana* as previously described. The healthy leaves were sprayed with 1 mM D‐luciferin and incubated in the dark for 5 min, and LUC images were captured using a CCD imager (Tanon‐5200).

### 4.10 Co‐Immunoprecipitation Assay

The fusion expression vectors pCAMBIA2300‐*VqCu/ZnSOD1*/*VqCAT2*‐FLG and pCAMBIA2300‐*VqLecRKV.4*/*VqBAK1*‐GFP were constructed and subsequently transformed into *Agrobacterium* strain GV3101 for infiltration into *N. benthamiana*. Total proteins were extracted from healthy leaves, and protein expression was verified to ensure robust production of each target protein prior to proceeding with the immunoprecipitation (IP) experiment. The detailed procedure for the co‐immunoprecipitation (Co‐IP) assay was as follows: Once input experiment confirmed the correct expression and size of the proteins, 1 mL of the stored protein solution was incubated with 4 μL GFP tag primary antibody (HT801‐01, TransGen Biotech) at 4°C for 3 h. Then 50 μL of immunoprecipitation protein A/G magnetic beads (Protein A/G plus agarose: sc‐2003) were added and the mixture was incubated at 4°C for an additional 12 h. The supernatant was removed after washing the beads three to four times with pre‐cooled protein extraction buffer. To eliminate interference from light chains, the Anti‐Mouse IgG HCS antibody was replaced, and the subsequent steps were performed according to standard Co‐IP protocols.

### 4.11 Transient Gene Expression Analysis in Grapevine Leaves

Fusion expression vectors pCAMBIA2300‐*VqCu/ZnSOD1* and pART27‐*VqCu/ZnSOD1* were constructed. The fusion vectors were injected into grapevine leaves. Detailed procedures for the transient transformation experiment were as previously described (Wang, Wang, et al. 2023).

## Author Contributions

X.W. and Y.L. designed this study. Y.L. and R.L. conducted most of the experiments and data analysis. Z.L., W.Y., R.L. and C.J. participated in some experiments. Z.L. assisted in analysing the experimental data. X.W. provided guidance to the study. Y.L., Y.Z. and X.W. wrote and revised the manuscript. Y.L. and R.L. contributed equally to this work.

## Funding

This work was supported by National Natural Science Foundation of China (32272693, 31872071), China Postdoctoral Science Foundation (2023M732896), Natural Science Basic Research Program of Shaanxi Province (2025JC‐QYCX‐023), Program for Innovative Research Team of Grape Germplasm Resources and Breeding (2013KCT‐25).

## Conflicts of Interest

The authors declare no conflicts of interest.

## Supporting information


**Figure S1:** Position of G‐type *LecRK* genes in the *Vitis vinifera* grapevine chromosome.
**Figure S2:** Phylogenetic tree analysis of full‐length amino acid sequences of G‐type *LecRK* genes, using one L‐LecRK and one C‐LecRK sequence as outgroups.
**Figure S3:** Conserved motifs analysis of grapevine G‐type LecRKs.
**Figure S4:** Information of the *VqLecRKV.4* genes.
**Figure S5:** Subcellular localisation of the VqLecRKV.4‐GFP fusion protein.
**Figure S6:** Overexpressing *VqLecRKV.4* enhances resistance to powdery mildew in *A. thaliana* .
**Figure S7:** Genetic transformation of *VqLecRKV.4* into *V. vinifera* L. cv. ‘Thompson Seedless’.
**Figure S8:** Identification of *VqLecRKV.4* transgenic lines.
**Figure S9:** Overexpressing *VqLecRKV.4* enhances resistance to powdery mildew in *V. vinifera*.
**Figure S10:** Relative expression levels of genes (*VvrbohC2*, *VvEDS1*, *VvPR5*) were evaluated by qRT‐PCR in the leaves of *VqLecRKV.4*‐overexpressing (#61, #68), *VqLecRKV.4*‐RNAi (#10, #23) and wild‐type plants after *E. necator* inoculation.
**Figure S11:** KEGG pathway analysis of proximal proteins associated with VqLecRKV.4 based on TurboID‐mediated labelling.
**Figure S12:** The DUAL membrane system assay showing that VqLecRKV.4 does not interact with the candidate prey proteins.
**Figure S13:** Sequence characteristics of VqCu/ZnSOD1.
**Figure S14:** The expression of VqCu/ZnSOD1 in transiently transformed grapevine leaves at various time points following powdery mildew infection.
**Figure S15:** Bioinformatic analysis of VqLecRKV.4 phosphorylated proteins.
**Figure S16:** Genetic transformation of RNAi‐*VqBAK1* into *V. vinifera* L. cv. ‘Thompson Seedless’.
**Figure S17:** VqBAK1 controlled cell death in OE‐*VqLecRKV.4* grapevine induced by *E. necator* infection.
**Figure 18** The functional analysis of *VqCAT2* in response to powdery mildew in *V. vinifera* .
**Table S1:** The primers used in this study.
**Table S2:** Characteristics of grapevine G‐type *LecRK* genes.
**Table S3:** Classification and proposed nomenclature of grapevine G‐type LecRKs.
**Table S4:** Domains of grapevine G‐type LecRK proteins.
**Table S5:** The information of other proteins.
**Table S6:** The information of phosphoprotemoics oxidoreductases.
**Result S1**. Identification of grapevine G‐type *LecRK* gene family.
**Result S2** Phylogenetic analysis and domains identification of grapevine G‐type LecRKs.
**Result S3** Overexpressing *VqLecRKV.4* enhances resistance to powdery mildew in *A. thaliana* .

## Data Availability

Sequence data can be found in the NCBI website (https://www.ncbi.nlm.nih.gov/) and the Ensembl Plants (http://plants.ensembl.org/Vitis_vinifera/Info/Index) using the accession numbers in Tables S2, S5 and S6. The mass spectrometry proteomics data have been deposited to the ProteomeXchange Consortium via the iProX partner repository with the dataset identifier PXD062666 (https://proteomecentral.proteomexchange.org).
