# Supplementary material for: VqLecRKV.4 and VqBAK1 Modulate Grapevine Resistance to Powdery Mildew by Regulating Dynamic Balance of ROS
Source: Plant Biotechnol J. 2026 Feb 18;24(6):3691–707. doi: 10.1111/pbi.70595 (PMC13205748; doi:10.1111/pbi.70595)
Supplement: Supplementary file 1 — Figure S1: Position of G‐type LecRK genes in the Vitis vinifera grapevine chromosome. Figure S2: Phylogenetic tree analysis of full‐length amino acid sequences of G‐type LecRK genes, using one L‐LecRK and one C‐LecRK sequence as outgroups. Figure S3: Conserved motifs analysis of grapevine G‐type LecRKs. Figure S4: Information of the VqLecRKV.4 genes. Figure S5: Subcellular localisation of the VqLecRKV.4‐GFP fusion protein. Figure S6: Overexpressing VqLecRKV.4 enhances resistance to powdery mildew in A. thaliana . Figure S7: Genetic transformation of VqLecRKV.4 into V. vinifera L. cv. ‘Thompson Seedless’. Figure S8: Identification of VqLecRKV.4 transgenic lines. Figure S9: Overexpressing VqLecRKV.4 enhances resistance to powdery mildew in V. vinifera. Figure S10: Relative expression levels of genes (VvrbohC2, VvEDS1, VvPR5) were evaluated by qRT‐PCR in the leaves of VqLecRKV.4‐overexpressing (#61, #68), VqLecRKV.4‐RNAi (#10, #23) and wild‐type plants after E. necator inoculation. Figure S11: KEGG pathway analysis of proximal proteins associated with VqLecRKV.4 based on TurboID‐mediated labelling. Figure S12: The DUAL membrane system assay showing that VqLecRKV.4 does not interact with the candidate prey proteins. Figure S13: Sequence characteristics of VqCu/ZnSOD1. Figure S14: The expression of VqCu/ZnSOD1 in transiently transformed grapevine leaves at various time points following powdery mildew infection. Figure S15: Bioinformatic analysis of VqLecRKV.4 phosphorylated proteins. Figure S16: Genetic transformation of RNAi‐VqBAK1 into V. vinifera L. cv. ‘Thompson Seedless’. Figure S17: VqBAK1 controlled cell death in OE‐VqLecRKV.4 grapevine induced by E. necator infection. Figure 18 The functional analysis of VqCAT2 in response to powdery mildew in V. vinifera . Table S1: The primers used in this study. Table S2: Characteristics of grapevine G‐type LecRK genes. Table S3: Classification and proposed nomenclature of grapevine G‐type LecRKs. Table S4: Domains of grapevin [file PBI-24-3691-s001.doc]

Supporting Information

**VqLecRKV.4 and VqBAK1 modulate grapevine resistance to powdery mildew by regulating dynamic balance of ROS**

Yajuan Li^1,2†^, Ruilin Li^1,2†^, Zhuoyu Liu^1,2^, Kewei You^1,2^, Rongxin Li^1,2^, Chen Jiao^1,3^ , Zhenjiang Wei^1,2^, Zhi Li^1,2^, Yijie Zhao^1,2*^, Xiping Wang^1,2*^

^1^State Key Laboratory for Crop Stress Resistance and High-Efficiency Production, College of Horticulture, Northwest A&F University, Yangling, Shaanxi, China;

^2^Key Laboratory of Horticultural Plant Biology and Germplasm Innovation in Northwest China, Ministry of Agriculture, Northwest A&F University, Yangling, Shaanxi, China;

^3^Key Laboratory of Molecular Biology of Crop Pathogens and Insects, Institute of Biotechnology, Zhejiang University, Hangzhou, 310058, China

*Correspondence ( Tel 86-29-87082129; Fax 86-29-87082613; email [wangxiping@nwsuaf.edu.cn](mailto:wangxiping@nwsuaf.edu.cn) _(X.W.)_; Tel 18306070195; Fax 86-29-87082613; email [yijie.zhao@nwsuaf.edu.cn](mailto:yijie.zhao@nwsuaf.edu.cn) (Y.Z.)

^†^These authors contributed equally to this work.

**Supporting Results**

**Result S1 Identification of grapevine G-type LecRK gene family**

To establish a robust phylogenetic framework for the G-type *LecRK* family, we identified its members using the latest reference genome of *Vitis vinifera* (PN40024.T2T (v5) assembly)(Shi et al., 2023). The amino acid sequence of the B-lectin domain (PF01453) was utilized as a query for a BLASTp search against the *V. vinifera* proteome. Subsequently, all candidate proteins were rigorously verified for the presence of both a lectin domain and a kinase domain using the MEME database (https://meme-suite.org/tools/meme). Through this process, a total of 94 non-redundant G-type *LecRK* genes were identified (Table S2). Their chromosomal locations are depicted in Figure S1. This *V. vinifera*-centered analysis provides a reference framework for the classification of *V.* *quinquangularis*-derived *LecRK* genes investigated in this study.

**Result S2 Phylogenetic analysis and domains identification of grapevine G-type LecRKs**

In order to understand the evolution of G-type LecRK family members in grapevine, a phylogenetic tree was constructed using neighbor-joining (NJ) method, encompassing 94 G-type LecRKs, 1 L-type LecRK and 1 C-type. The 94 G-type LecRK family members were classified into ten distinct groups, designated I to X. Groups I, III, V and X each comprised 13 G-type LecRKs, collectively representing 55% of the total G-type LecRKs. Groups II, IV, VI, VII and IX contained a combined total of 39 G-type LecRKs, accounting for 42% of the total G-type LecRKs. Notably, group VIII included only 3 G-type LecRKs, making up the remaining 3% (Figure S2). Following the nomenclature system of *Arabidopsis* G-type LecRKs (Teixeira et al., 2018), the G-type *VqLecRK* genes were named based on their gene number and chromosomal location (Table S3).

Twenty-one conserved motifs were identified within the G-type VqLecRK family using MEME to analyze motif composition and diversity (https://meme-suite.org/meme/tools/meme) (Bailey et al., 2009). Notably, all VqLecRKs exhibited a fundamental B-lectin motif, consistent with previous result (Van Damme et al., 2007), as well as a transmembrane (TM) domain and a kinase motif. Additionally, 66 of these proteins possessed S-locus glycoprotein (SLG) motifs, 43 exhibited epidermal growth factor (EGF) motifs, 77 contained apple-like domains found in plasminogen (PAN) motifs, and 73 included single peptide (SP) motifs (Figure S3 and Table S4).

**Result S3 Overexpressing *VqLecRKV.4* enhances resistance to powdery mildew in *A. thaliana***

In order to study the specific function of the *VqLecRKV.4* gene, we constructed a plant overexpression vector containing *VqLecRKV.4* and transferred it into *A. thaliana* via the *Agrobacterium* floral dip method. Seedlings that successfully received the overexpression vector exhibited normal growth on hygromycin-selective medium, whereas wild-type seedlings did not grow (Figure S6a). Those seedlings that grew welly on the selective medium were transplanted into a greenhouse. When the plants developed four to six true leaves, their leaves were collected for DNA and RNA extraction. The *VqLecRKV.4* gene expression was confirmed in the transgenic *A. thaliana* lines of the T1 generation (Figure S6b).

In this study, *Golovinomyces cichoracearum* (UMSG1), which can infect *Arabidopsis thaliana*, was artificially inoculated to assess potential differences in powdery mildew resistance between the wild-type Col-0 and T3 generation transgenic lines overexpressing *VqLecRKV.4* (OE1 and OE2). At 8 d post-inoculation, transgenic lines (OE1 and OE2) exhibited markedly reduced susceptibility compared to wild-type Col-0 (Figure S6c). As shown in Figure S6c, localized yellowing appeared on the leaves of OE1 and OE2, a symptom absent in wild-type plants. Furthermore, spore concentrations on the leaves of OE1 and OE2 were less than those on Col-0 plants (Figure S6d). To compare the responses of wild-type (Col-0) and transgenic lines (OE1 and OE2) to powdery mildew, cell death and reactive oxygen species were observed at 8 days post-inoculation. Few dead cells were observed in wild-type leaves, whereas large clusters of dead cells were evident in both OE1 and OE2 transgenic lines. DAB staining revealed hydrogen peroxide (H_2_O_2_) accumulation following inoculation: only minimal H_2_O_2_ was detected in wild-type leaves, while substantially higher and more spatially concentrated accumulation was observed in OE1 and OE2 leaves (Figure S6e).

**Supporting methods**

**Bioinformatics**

To identify G-type *LecRK* genes in grapevine, we utilized the B-lectin (PF01453) amino acid sequence of LOC100262073, derived from previous transcriptome data, as our reference sequence. We conducted searches for representative protein sequences using this domain in both the NCBI database (https://www.ncbi.nlm.nih.gov/) and the Grapedia website (https://grapedia.org/genomes/). All potential G-type LecRK protein sequences were then validated using MEME (https://meme-suite.org/tools/meme) to ensure the presence and integrity of conserved motifs. This reference genome was utilized solely to provide phylogenetic and genomic context for the LecRK family, thereby facilitating the classification of genes identified from wild *Vitis* species.

A total of 94 G-type LecRK protein sequences were downloaded, with the L-type LecRK (LOC100854743) and C-type LecRK (LOC100245331) sequences designated as outgroups. The phylogenetic tree of the G-type LecRKs was constructed using MEGA7.0 software, applying the Neighbor-Joining method with a bootstrap test repeated 1,000 times. Based on both the G-type LecRK protein sequence data and comparative analysis of the phylogenetic tree, the 94 G-type LecRKs were grouped and classified accordingly. These genes were named in accordance with the nomenclature similar to that of *Arabidopsis* G-type LecRKs.

**Grapevine leaves treatment with powdery mildew**

The experiment utilized grapevine powdery mildew (*Erysiphe necator*) as the subject. Each experiment was conducted three times to ensure reliability. Healthy grapevine leaves were selected as inoculation material with *E. necator*, applied through the pressing method as described by Li et al (Li et al., 2022). Leaves were collected at 0, 6, 12, 24, 48 and 96 hours post-inoculation. The control group comprised grape leaves sprayed with sterile water, collected at the same time points as the treatment group At each time point for all treatments, six leaves were selected from three distinct grapevine plants. All harvested leaves were immediately frozen at −80°C for further analysis.

**RNA extraction and Real-Time Quantitative PCR (RT-qPCR)**

Total RNA was extracted from grapevine leaves using the plant RNA extraction kit from Omega Company. The purity and concentration of the RNA were evaluated through 1% agarose gel electrophoresis. Subsequently, reverse transcription was performed using the *TransScript*® RT Uni All-in-One First-Strand cDNA Synthesis SuperMix (Yugong Biolabs Inc., Jiangsu, China).

Semi-quantitative reverse-transcription (RT) PCR results were visualized as a red-green heat map generated by HemI1.0 software. Real-time quantitative PCR was conducted on an IQ5 real-time PCR machine (Bio-Rad, Hercules, CA, USA) using *TransScript*TM Green qPCR SuperMix (Yugong Biolabs Inc.). Each reaction had a final volume of 20 μL. The grapevine *Actin1* gene (NC_012010) was used as an internal control. All experimental procedures were conducted according to previously published protocols(Zhu et al., 2019) Gene-specific primers were designed using Primer Premier 5.0 (Table S1). Three independent experimental replicates were performed for each cDNA sample.

**Subcellular localization**

Using cDNA from the leaves of the Chinese wild *V. quinquangularis* 'Shang-24' as a template, the complete sequences of the *VqLecRKV.4*, *VqBAK1*, *VqCu/ZnSOD1* and *VqCAT2* gene fragments were amplified via PCR. *VqLecRKV.4* and *VqBAK1* were utilized in constructing the pCAMBIA2300-GFP and 5635-pSuper1300-cherry vectors, while *VqCu/ZnSOD1* and *VqCAT2* were used to construct the pCAMBIA2300-GFP vector. All fusion vectors were designed for subcellular localization. The primers employed for vector construction are listed in Table S1. The fusion vectors were then separately co-transformed into *N. benthamiana* as previously described. Fluorescence in *N. benthamiana* was examined using a laser scanning confocal microscope on donor excitation wavelength (488 nm) and acceptor excitation wavelength (552 nm), and the images were processed using LAS X software (Leica, Germany). All experiments were conducted in triplicate.

**Quantitative phosphoproteomics of grapevine leaves**

Leaves from the wild type and OE-*VqLecRKV.4* lines were collected at 6 hours after infection with powdery mildew. Proteins were redissolved in 8 M urea and their concentrations were measured using a BCA kit (Beyotime Biotechnology，Shanghai，China) following the manufacturer’s instructions. To precipitate the proteins, samples were slowly added to reach a final concentration of 20% (m/v) TCA, followed by vortex mixing and incubation for 2 hours at 4°C. Protein precipitates were harvested by centrifugation at 4,500 g for 5 minutes at 4°C. The precipitated proteins were washed 3 times with pre-cooled acetone and dried for 1 minute. The protein samples were then redissolved in 200 mM TEAB and ultrasonically dispersed. Digestion was initiated by adding trypsin at a 1:50 ratio of trypsin-to-protein mass for overnight incubation.The samples were reduced with 5 mM dithiothreitol for 30 minutes at 56°C and alkylated with 11 mM iodoacetamide for 15 minutes at room temperature in darkness. Finally, the peptides were desalted using a Strata X SPE column.

Peptide mixtures were initially incubated with IMAC microsphere suspension under agitation in loading buffer (50% acetonitrile/0.5% acetic acid). To remove non-specifically adsorbed peptides, the IMAC microspheres were sequentially washed with 50% acetonitrile/0.5% acetic acid followed by 30% acetonitrile/0.1% trifluoroacetic acid. For phosphopeptide elution, an elution buffer containing 10% NH_4_OH was added and the enriched phosphopeptides were subsequently released by agitation. The resulting supernatant, containing the phosphopeptides, was collected and lyophilized prior to LC-MS/MS analysis.

The process of Gene Ontology (GO) annotation utilizes the eggnog-mapper software to extract GO IDs from identified proteins based on the EggNOG database. Subsequently, functional classification and annotation analyses are conducted on these proteins according to cellular components, molecular functions, and biological processes. The KEGG pathway analysis encompasses categories such as metabolism, genetic information processing, environmental information processing, cellular processes, human diseases and drug development. Protein pathways annotation is performed using the KEGG pathway database, with proteins identified- through BLAST comparison (blastp, evalue ≤ 1e-4), for each sequence, annotation is assigned based on the top-scoring comparison result. Protein structural domain annotation was carried out for the identified proteins using the Pfam database in conjunction with the PfamScan tool.

**Grape powdery mildew inoculation and disease statistics**

Grape powdery mildew (*Erysiphe necator*) was maintained on leaves of ‘Thompson Seedless’ in this laboratory's greenhouse. Both transgenic and WT grapevines were cultivated in the greenhouse for 6-8 months before being used for powdery mildew inoculation. For inoculation, the leaf-contact method was employed: infected leaves exhibiting disease lesions were evenly pressed against five healthy leaves of each experimental vine, with the process repeated 3 to 4 times to ensure uniform and consistent distribution of the powdery mildew fungus. Subsequent to inoculation, phenotypic observations of the treated leaves were recorded, and the spore count (mg) on different plant lines was statistically analyzed, according to the spore-counting method described by Zang et al (Zhang et al., 2025). After inoculation, grapevine leaves were stained with trypan blue (to visualize spores and mycelium) and DAB (for hydrogen peroxide detection). The staining protocols were performed following the method of Wang et al (Wang et al., 2023). The contents of hydrogen peroxide (H_2_O_2_), as well as the activities of SOD and CAT enzyme, were measured using commercial assay kits and quantified by spectrophotometry in strict accordance with the manufacturer’s instructions.

**References**

**Supporting figures**

**
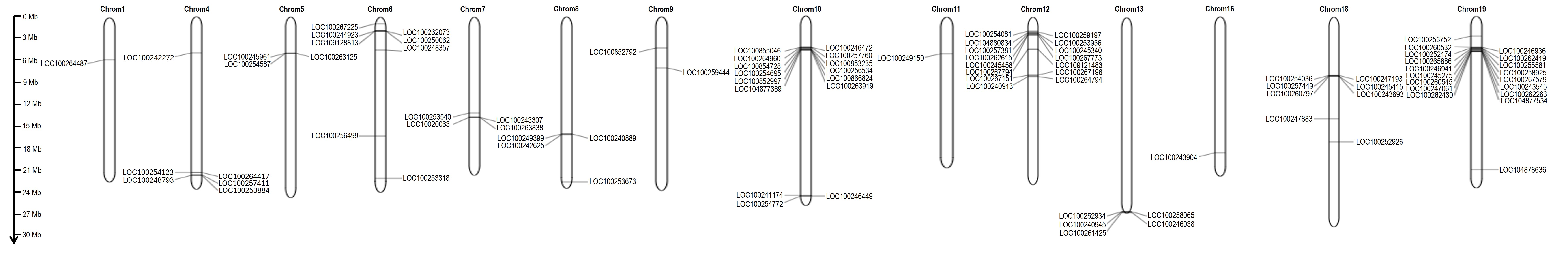
**

**Figure S1** Position of G-type *LecRK* genes in the *Vitis vinifera* grapevine chromosome.

**
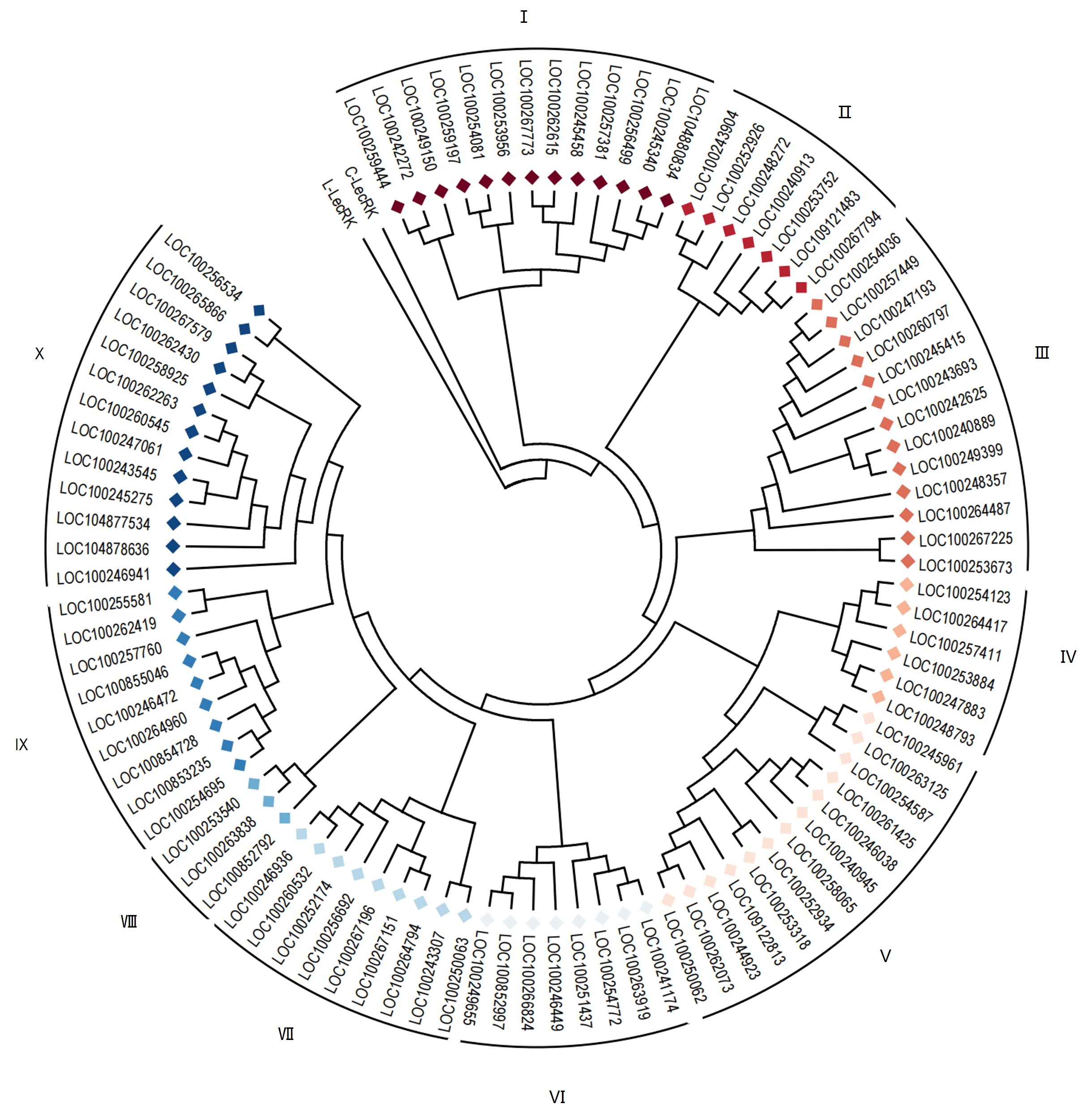
**

**Figure S2** Phylogenetic tree analysis of full-length amino acid sequences of G-type *LecRK* genes, using one L-LecRK and one C-LecRK sequence as outgroups. The phylogenetic tree was constructed by neighbor-joining method with 1000 bootstrap replicates. The labeled lines surrounding the tree indicate clade names as defined in the text.


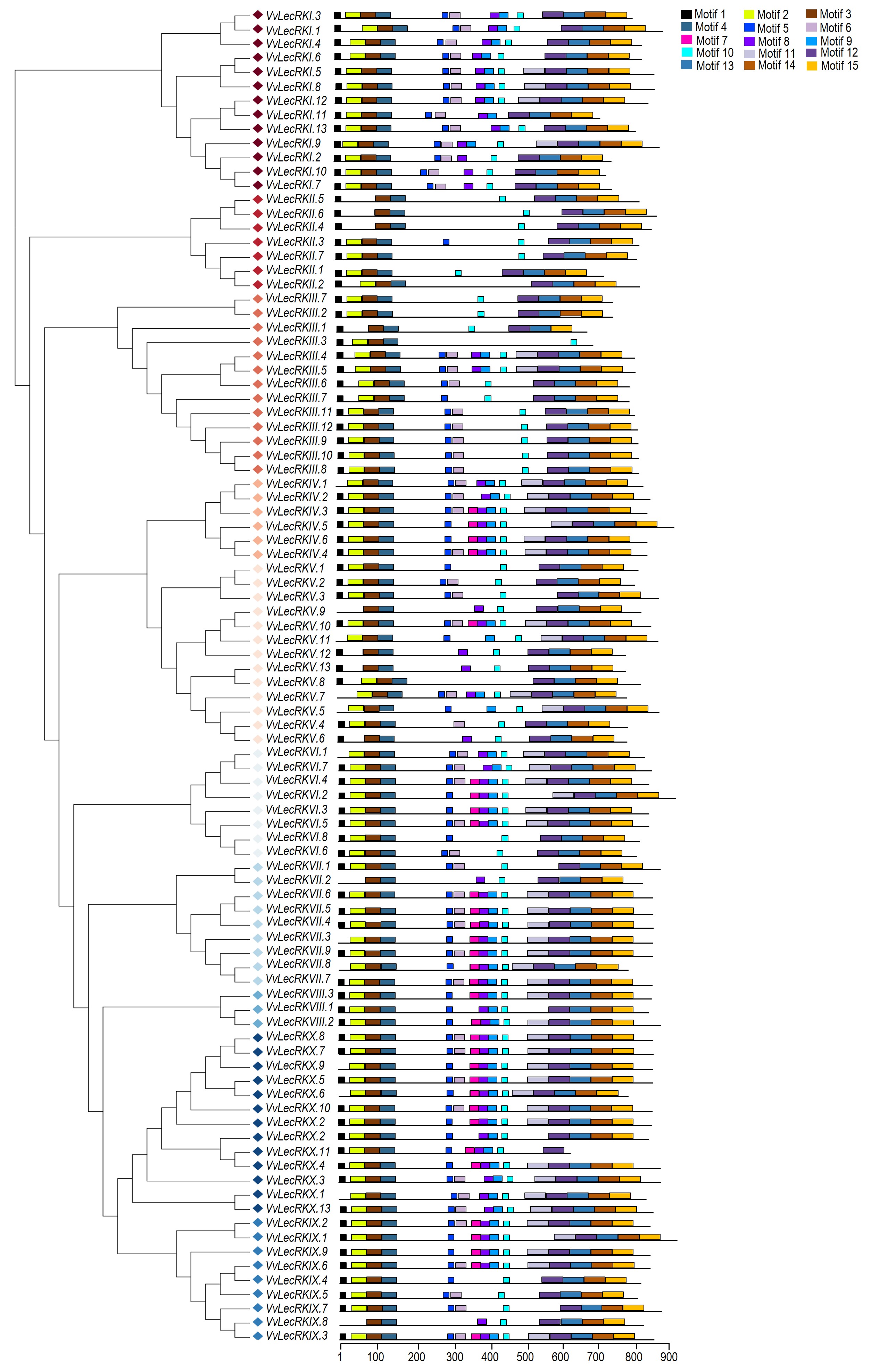


**Figure S3** Conserved motifs analysis of grapevine G-type LecRKs. Different color boxes represent different motifs.


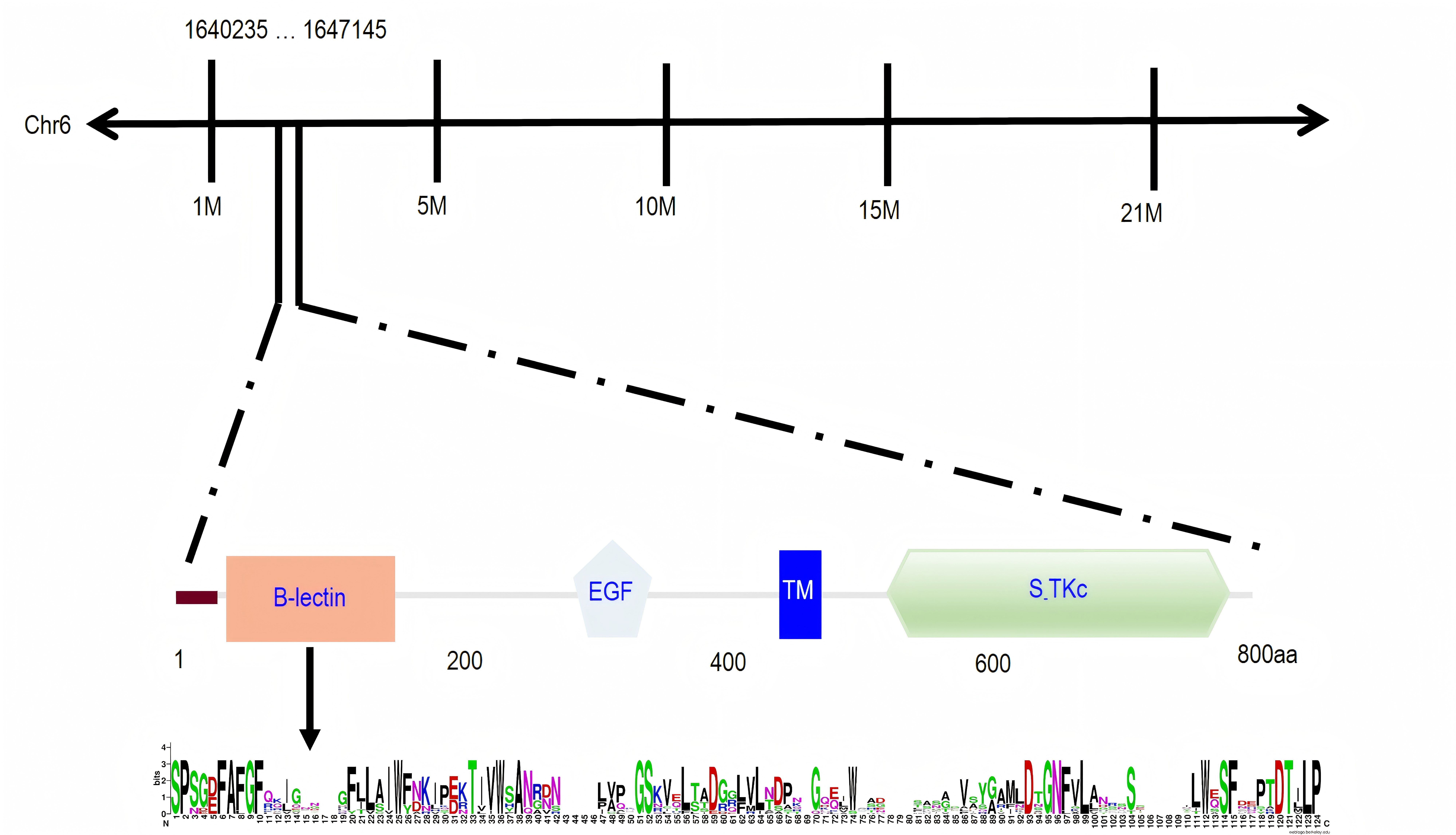


**Figure S4** Information of of the *VqLecRKV.4* genes. The chromosomal localization and the domains analysis of VqLecRKV.4.


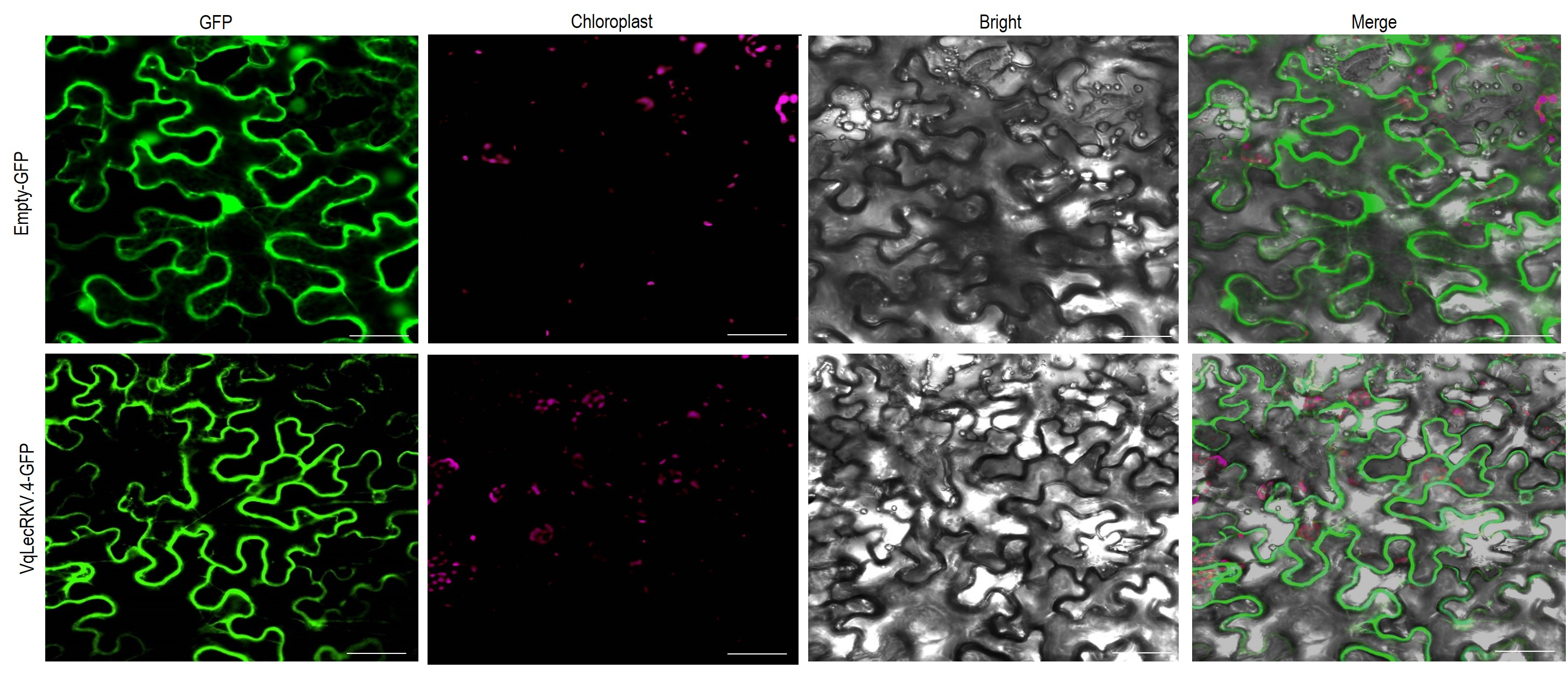


**Figure S5** Subcellular localization of the VqLecRKV.4-GFP fusion protein. Scale bars = 100 µm.


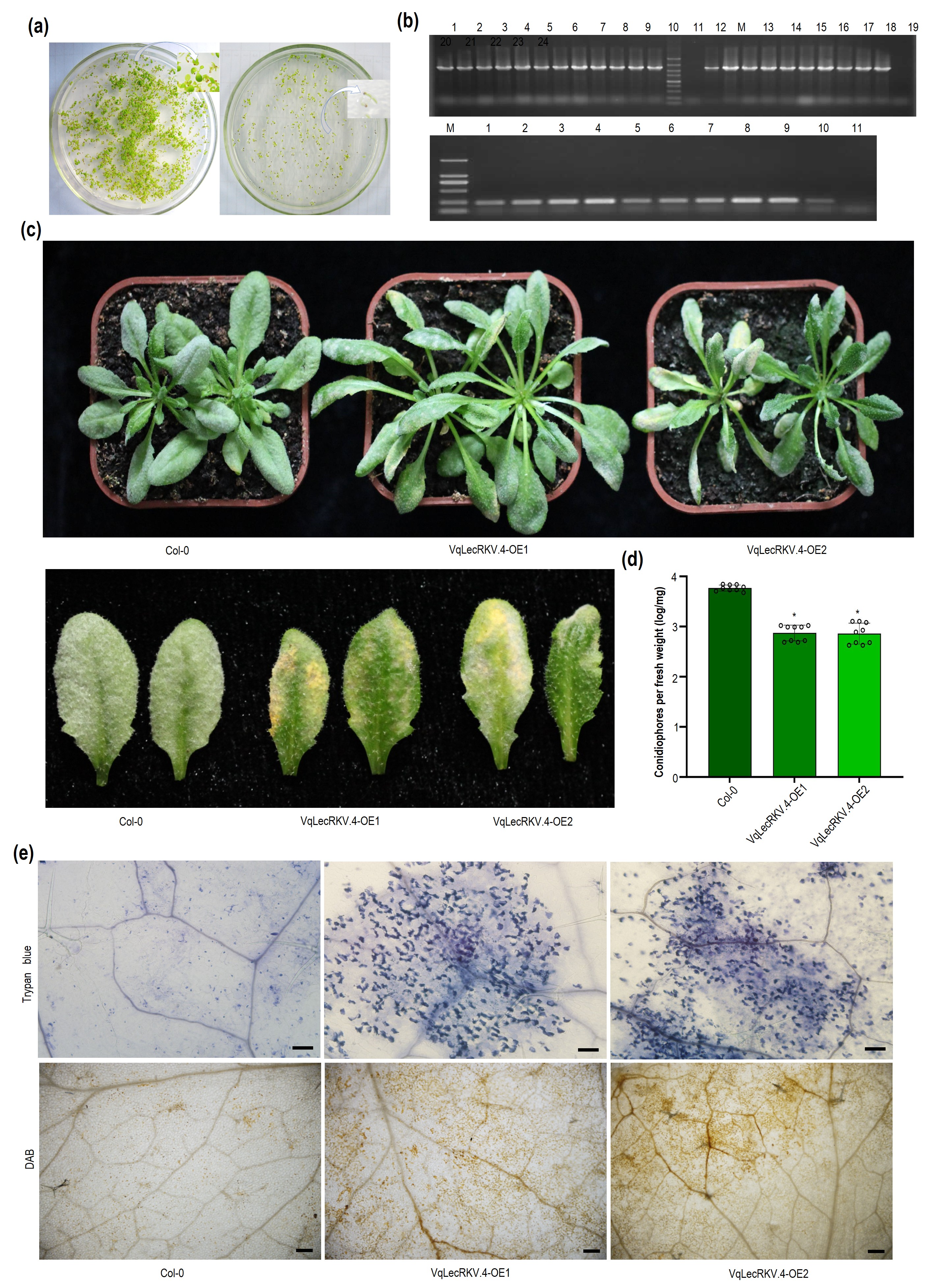


**Figure S6** Overexpressing *VqLecRKV.4* enhances resistance to powdery mildew in *A. thaliana*. (a) Screening of hygromycin-resistant transgenic *Arabidopsis* seedlings in the T1 generation. (b) Identification of *VqLecRKV.4* in the T1 generation using both DNA and mRNA analysis. Detection of *VqLecRKV.4* at the DNA level in the T1 generation. M: DNA Marker; lanes 1-12 and 14-23: *VqLecRKV.4* transgenic lines; lanes 13 and 24: *Arabidopsis* wild type. Analysis of *VqLecRKV.4* expression in T1 generations. M: DNA Marker; lanes 1-10: *VqLecRKV.4* transgenic lines; lane 11: *Arabidopsis* wild type. (c) Col-0 and overexpression lines at 8 dpi with *G.cichoracearum*. (d) Quantitative analysis of spore numbers pery milligram of fresh leaves at 8 dpi with *G.cichoracearum*. Values represent the means ± SD of three biological replicates, each with three technical replicates (n = 9). Asterisks indicate significant differences relative to the Col-0 wild-type control based on a t test (*P < 0.05). E) Dead cells and H_2_O_2_ production in *Arabidopsis* leaves at 8 dpi with *G.cichoracearum*. Scale bars = 100 μm.


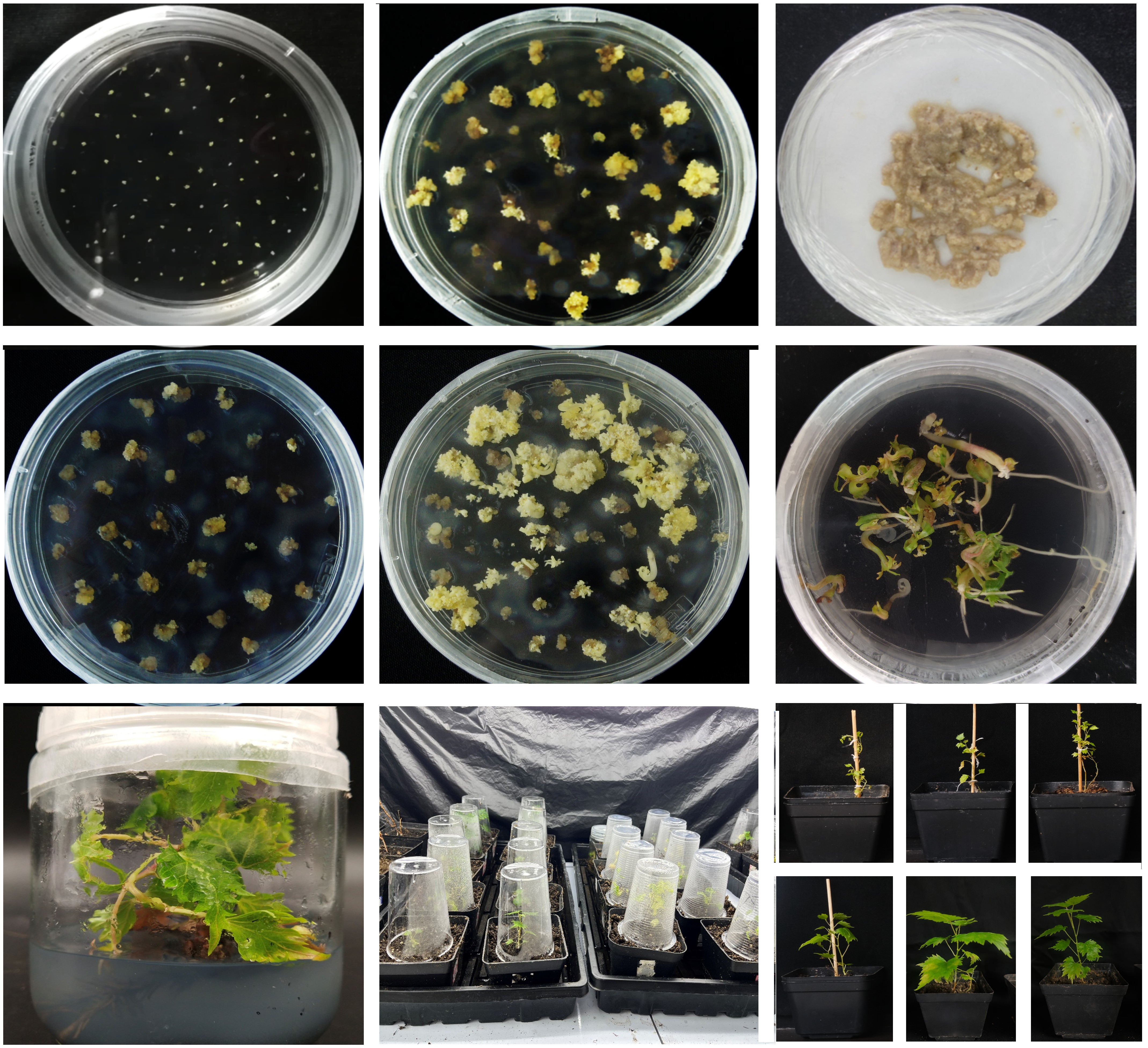


**Figure S7** Genetic transformation of *VqLecRKV.4* into *V. vinifera* L. cv. ‘Thompson Seedless’.

**
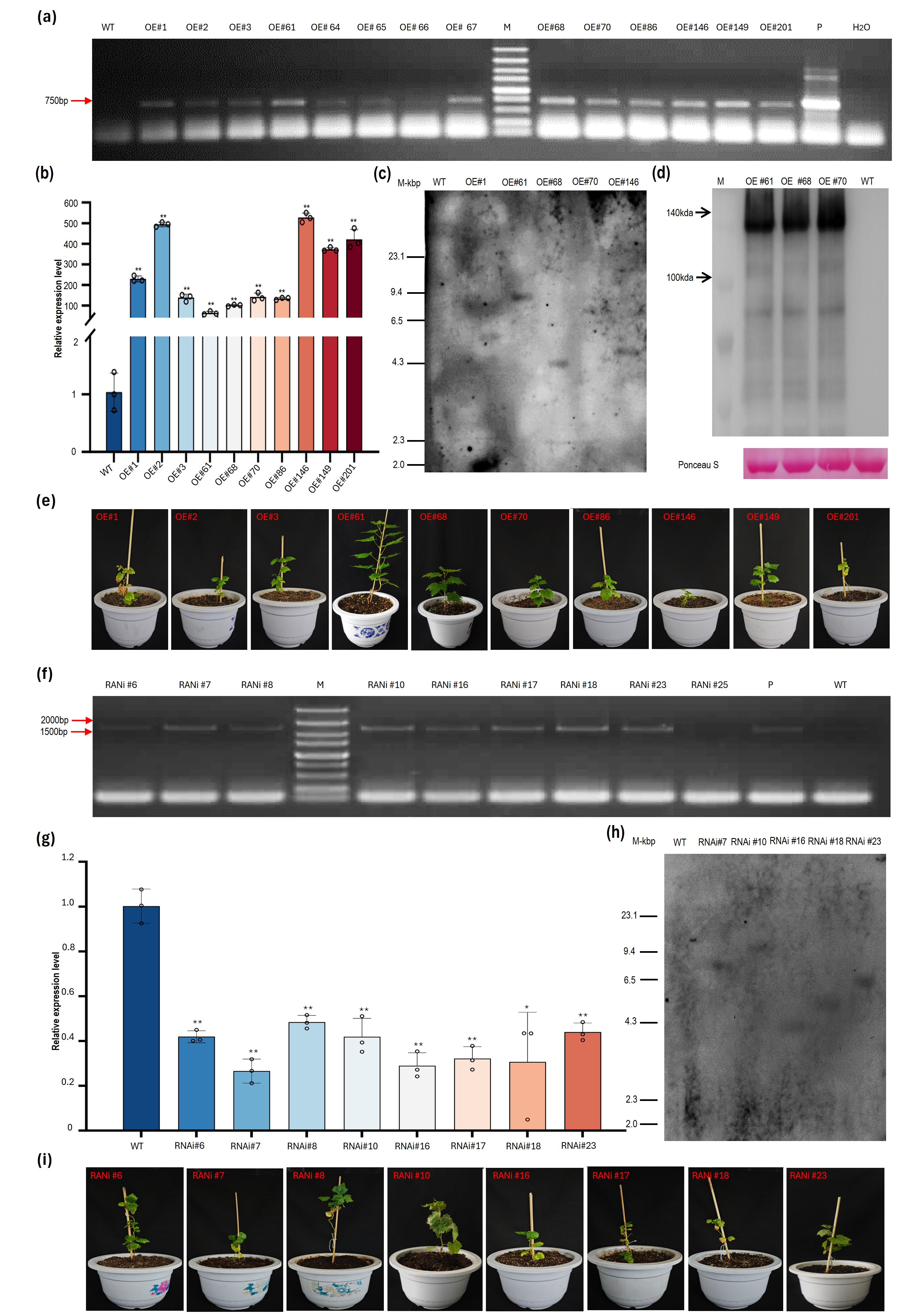
**

**Figure S8** Identification of *VqLecRKV.4* transgenic lines. (a) PCR analysis confirming the presence of OE-*VqLecRKV.4* in positive transgenic lines. (b) Assessment of *VqLecRKV.4* gene expression levels by real-time quantitative fluorescence PCR. Data are presented as means ± SD of three biological replicates (n = 3).(c) Southern blot analysis of positive OE-*VqLecRKV.4* transgenic lines. (d) Western blot analysis of positive OE-*VqLecRKV.4* transgenic lines. (e) Representative OE-*VqLecRKV.4* transgenic lines. (f) PCR analysis for the positive transgenic lines of RNAi-*VqLecRKV.4*. (f) Quantification of *VqLecRKV.4* gene expression in RNAi-*VqLecRKV.4* lines via real-time quantitative fluorescence PCR. Values are the means ± SD of three biological replicates (n = 3). (g) Southern blot analysis of positive RNAi-*VqLecRKV.4* transgenic lines. (h) Representative RNAi-*VqLecRKV.4* transgenic lines.


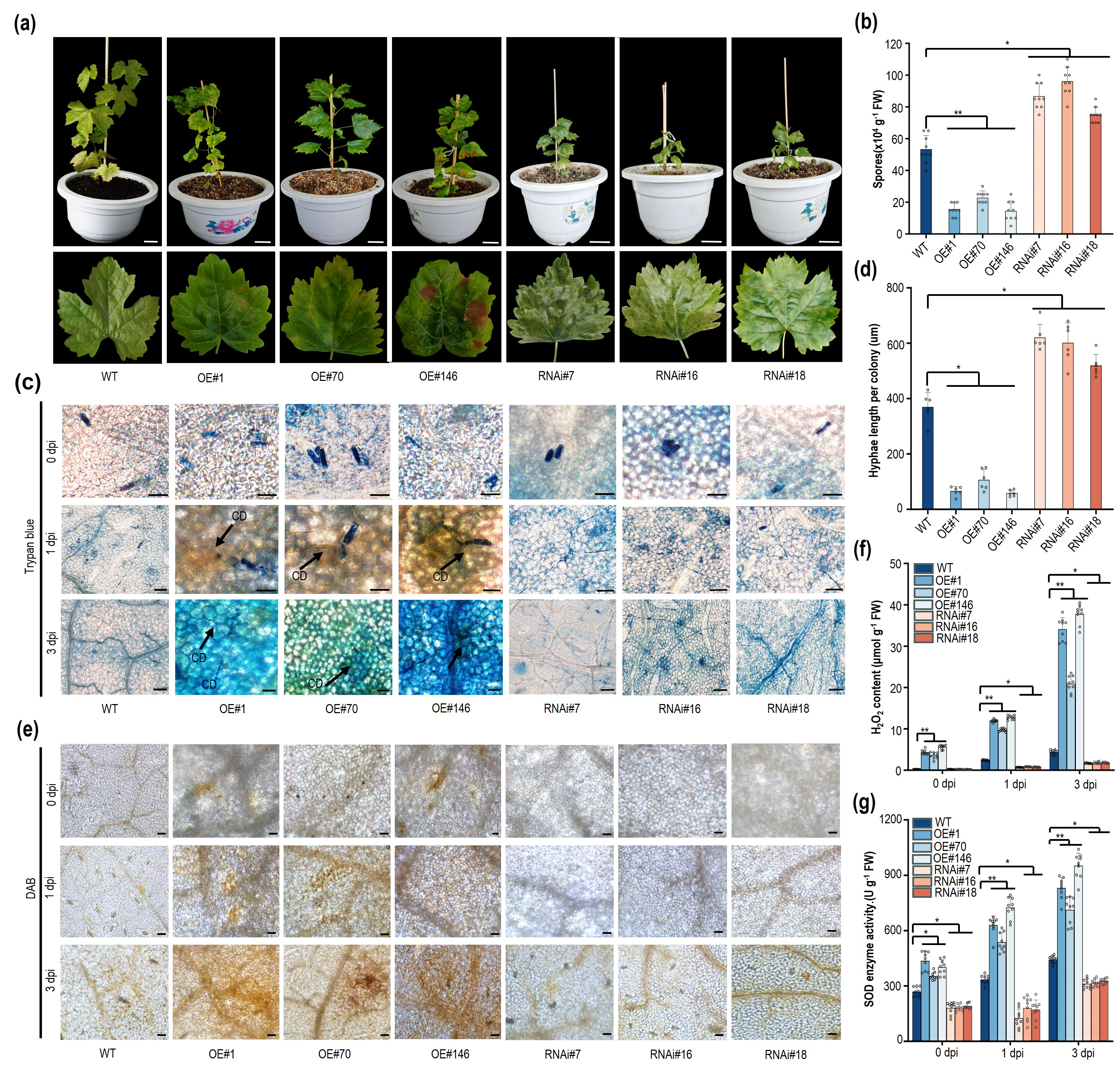


**Figure S9** Overexpressing *VqLecRKV.4* enhances resistance to powdery mildew in *V. vinifera.* (a) The phenotypes of wild type (WT), overexpressing *VqLecRKV.4* lines (OE1, OE70 and OE148), and RNAi-*VqLecRKV.4* lines (RNAi7, RNAi16 and RNAi18) at 11 days post- inoculation (dpi) with *E. necator.* (b) Quantitative analysis of spore numbers per milligram of fresh leaves at 11dpi with *E. necator.* Values are the means ± SD of three biological replicates, each with three technical replicates (n = 9).(c) Trypan blue stained observation of WT and transgenic *VqLecRKV.4* lines after *E. necator* infection; CD: cell death; Scale bars = 20 µm. (d) Average hyphal length per colony of *E. necator* on plant leaves at 1 dpi. Values are the means ± SD of three biological replicates, each with two technical replicates (n = 6). (e) 3,3-diaminobenzidine (DAB) staining for H_2_O_2_ production after *E. necator*-infected WT and transgenic *VqLecRKV.4* lines. Scale bars=50 μm. (f) H_2_O_2_ content of WT and transgenic *VqLecRKV.4* lines with *E. necator*. Values are the means ± SD of three biological replicates, each with three technical replicates (n = 9). (g) SOD enzyme activity in leaves of in *E. necator*-infected WT and transgenic *VqLecRKV.4* lines.Values are the means ± SD of three biological replicates, each with three technical replicates (n = 9). (b, d, f, g) Asterisks indicate statistical significance using a Tukey's multiple comparison test followed by one-way ANOVA (**P* < 0.05, ***P* < 0.01).


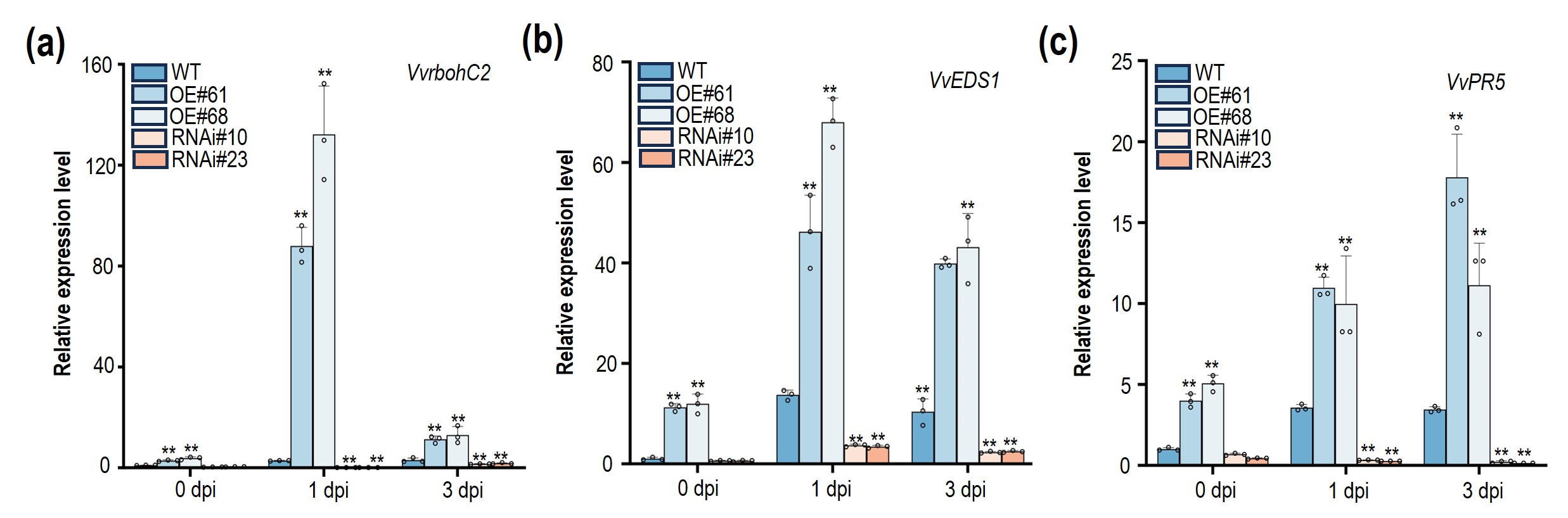


**Figure S10** Relative expression levels of genes (*VvrbohC2*, *VvEDS1*, *VvPR5*) were evaluated by qRT-PCR in the leaves of *VqLecRKV.4*-overexpressing (#61, #68), *VqLecRKV.4*-RNAi (#10, #23), and wild-type plants after *E. necator* inoculation.


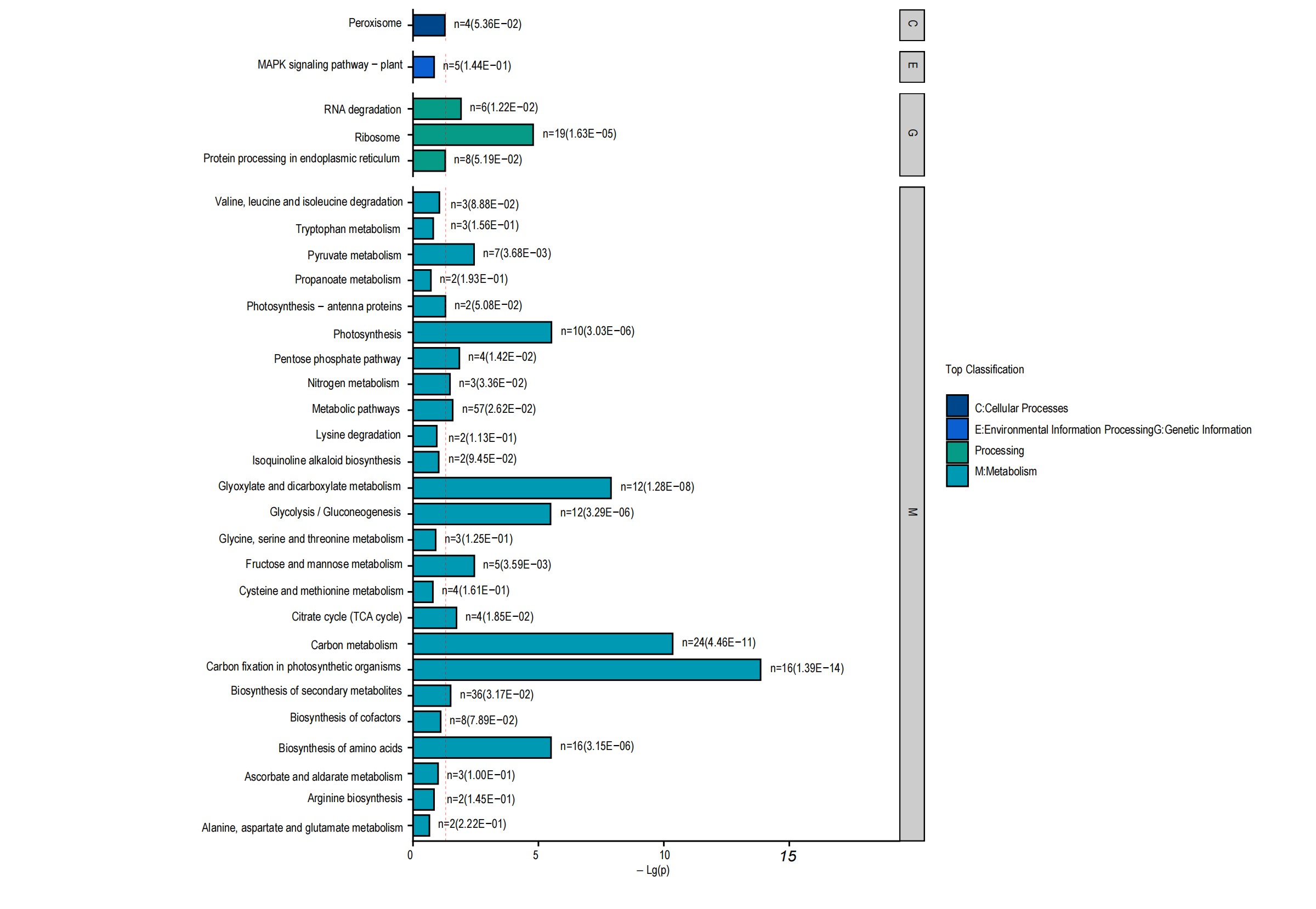


**Figure S11** KEGG pathway analysis of proximal proteins associated with VqLecRKV.4 based on TurboID-mediated labeling.

**
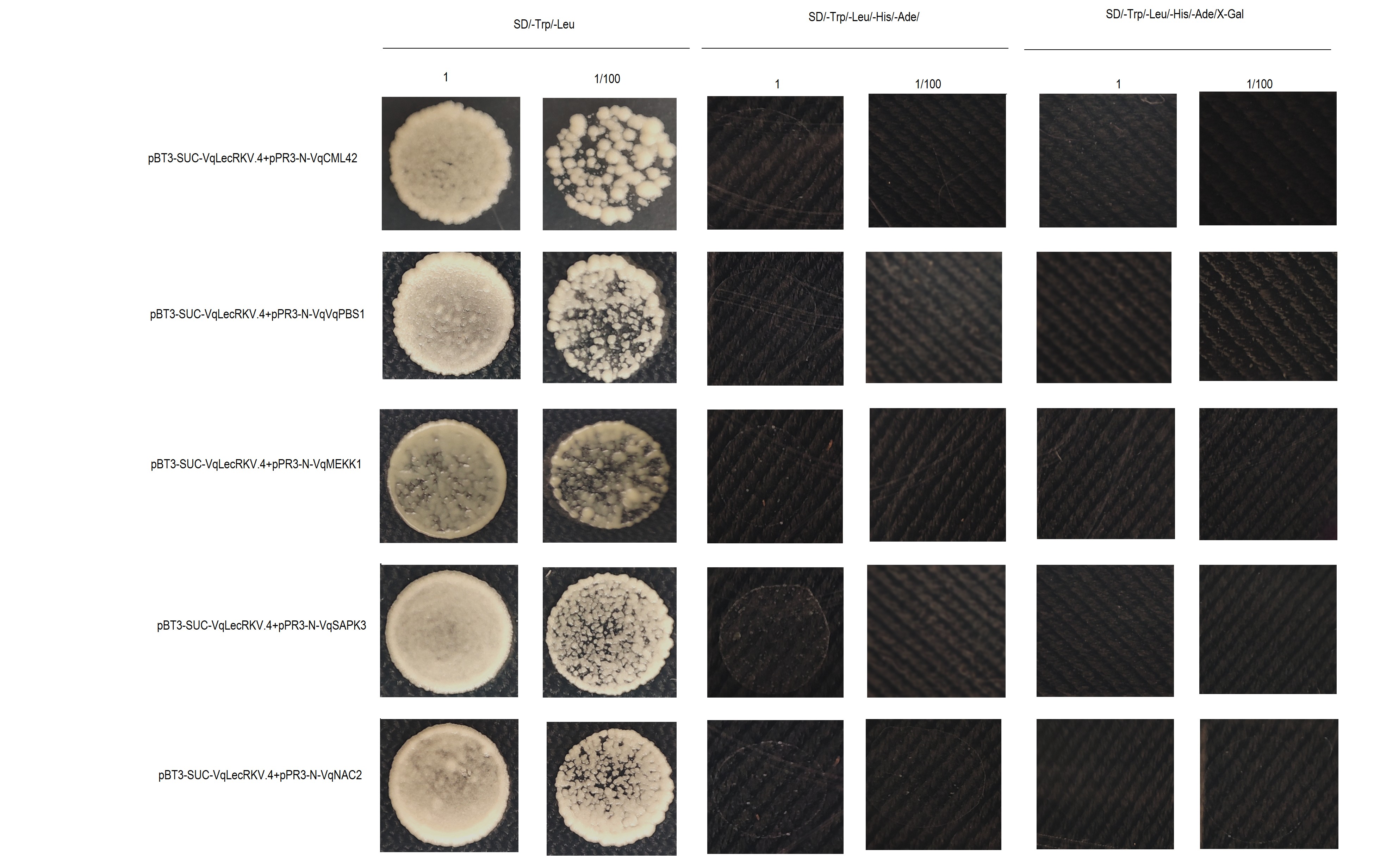
**

**Figure S12** The DUAL membrane system assay showing that VqLecRKV.4 does not interact with candidate prey proteins. pBT3-SUC-VqLecRKV.4 was used as baits, and pPR3-N was used as prey, and they were co-transformed into NMY51 yeast cells.


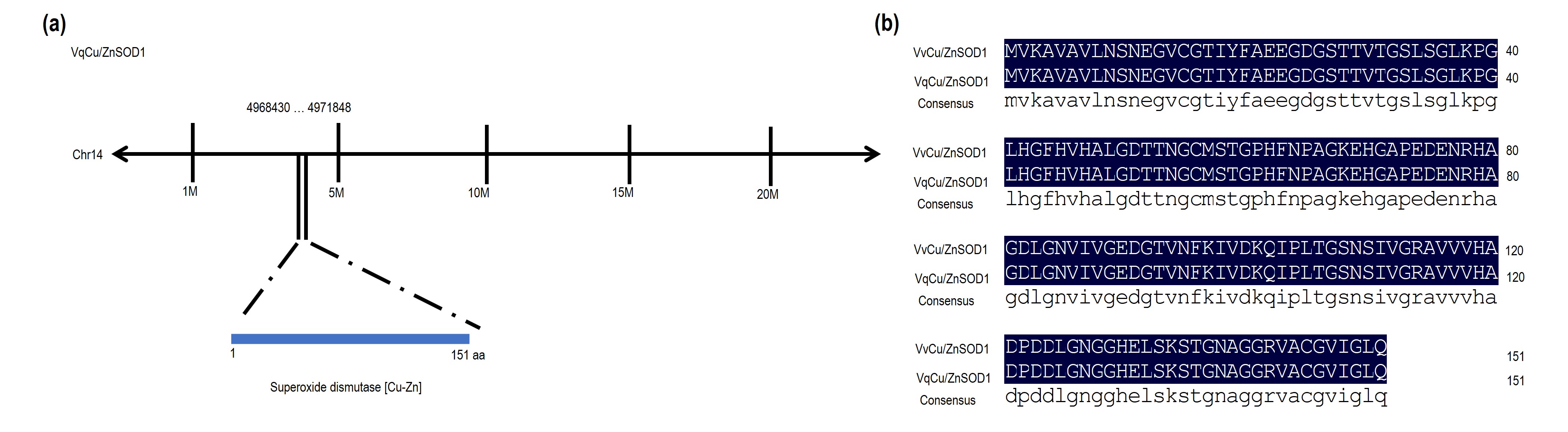


**Figure S13** Sequence characteristics of VqCu/ZnSOD1. (a) The chromosomal location of VqCu/ZnSOD1. (b) Protein sequence alignment between *V.* *quinquangularis* VqCu/ZnSOD1 and *V. vinifera* VqCu/ZnSOD1; differences are highlighted in blue.


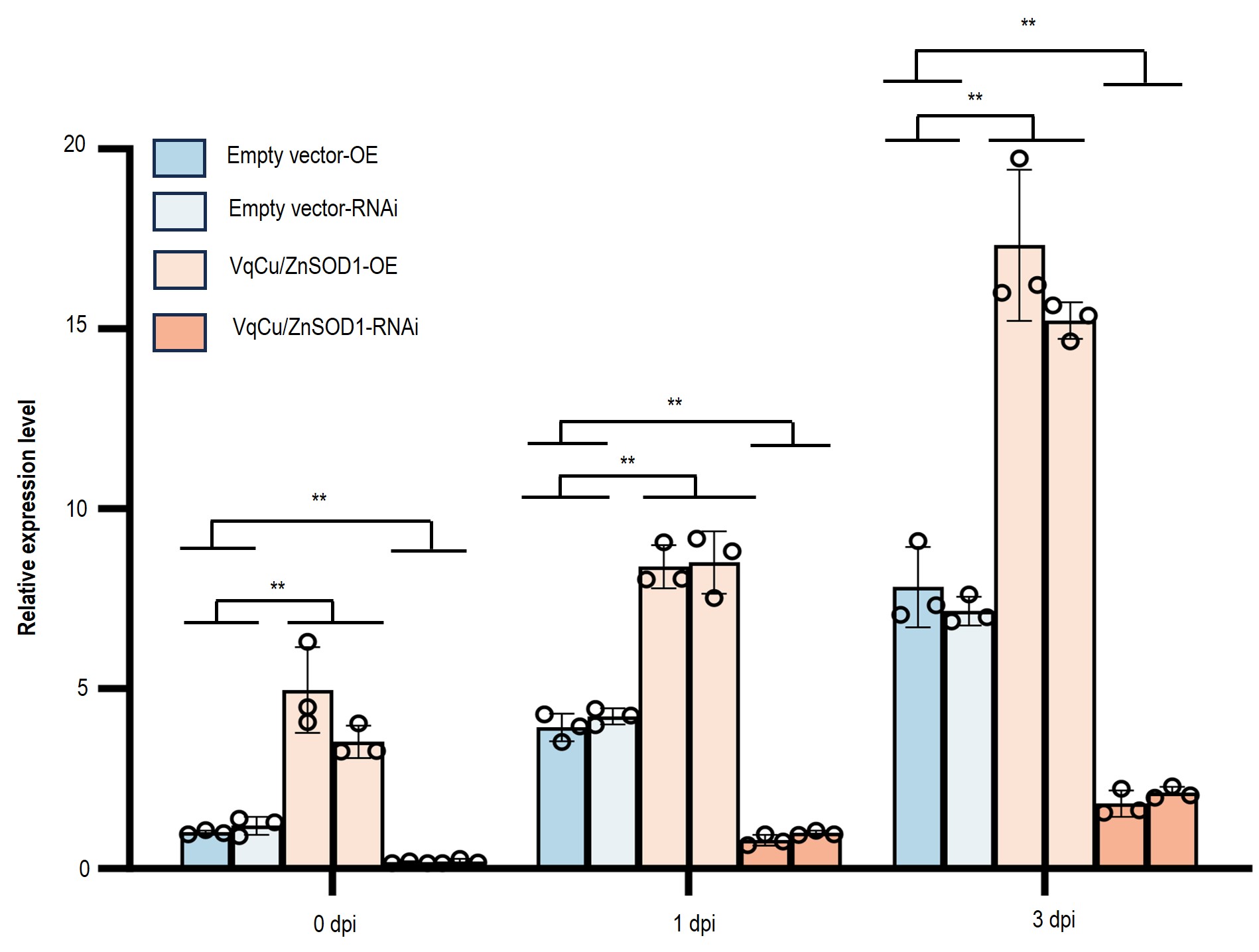


**Figure S14 T**he expression of VqCu/ZnSOD1 in transiently transformed grapevine leaves at various time points following powdery mildew infection. Values are the means ± SD of three biological replicates (n = 3). Asterisks indicate statistical significance using based on a t test (***P* < 0.01).


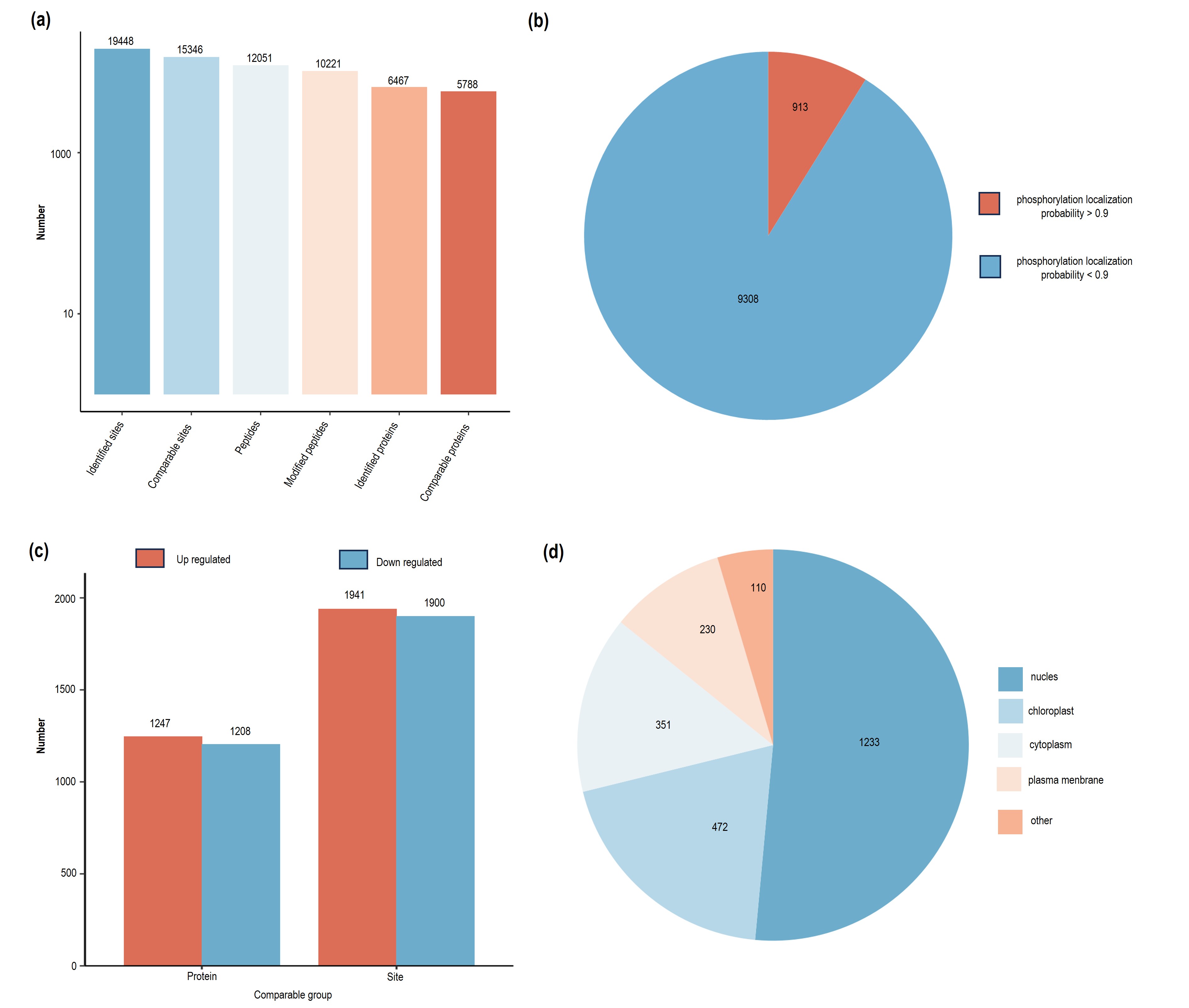


**Figure S15** Bioinformatics analysis of VqLecRKV.4 phosphorylated proteins. (a) Overview of modification site identification. (b) A total of 10,221 phosphopeptides were identified, among which 9,308 were classified as high-confidence phosphopeptides (phosphorylation localization probability > 0.9), and 913 were considered to have low phosphorylation localization probability (phosphorylation localization probability < 0.9). (c) Statistical chart illustrating the distribution of differential modification sites. (d) The numbers of differential modifications within various subcellular structures, with each subcellular compartment denoted by distinct colors in the chart.


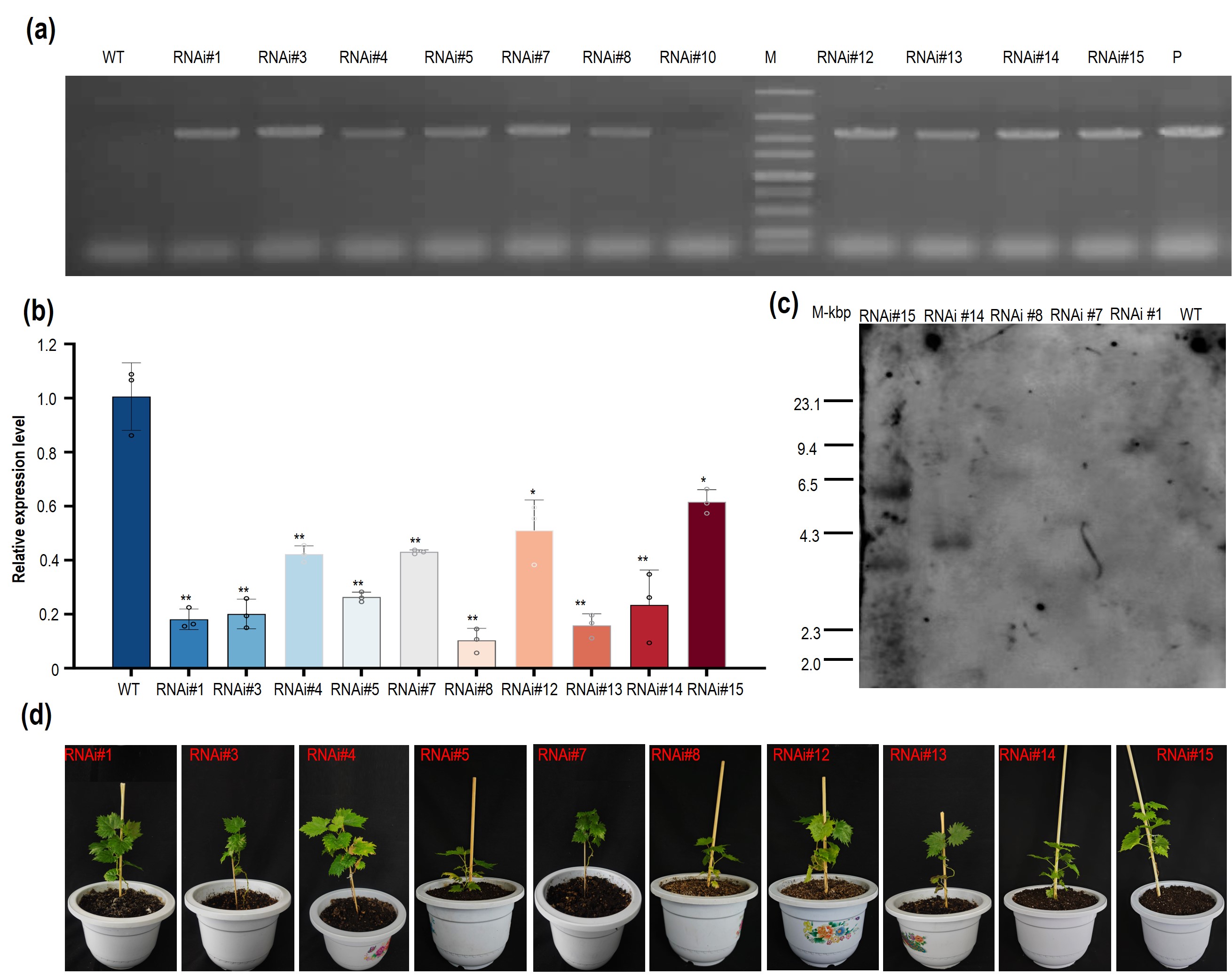


**Figure S16** Genetic transformation of RNAi-*VqBAK1* into *V. vinifera* L. cv. ‘Thompson Seedless’. (a) PCR analysis for the positive transgenic lines of *VqBAK1-RNAi*. (b) Detection of *VqBAK1* gene relative expression by real-time fluorescence quantitative PCR. Values are the means ± SD of three biological replicates (n = 3).(c) Southern blot analysis of the positive transgenic lines of *VqBAK1-*RNAi. (d) *VqBAK1-*RNAi transgenic lines.


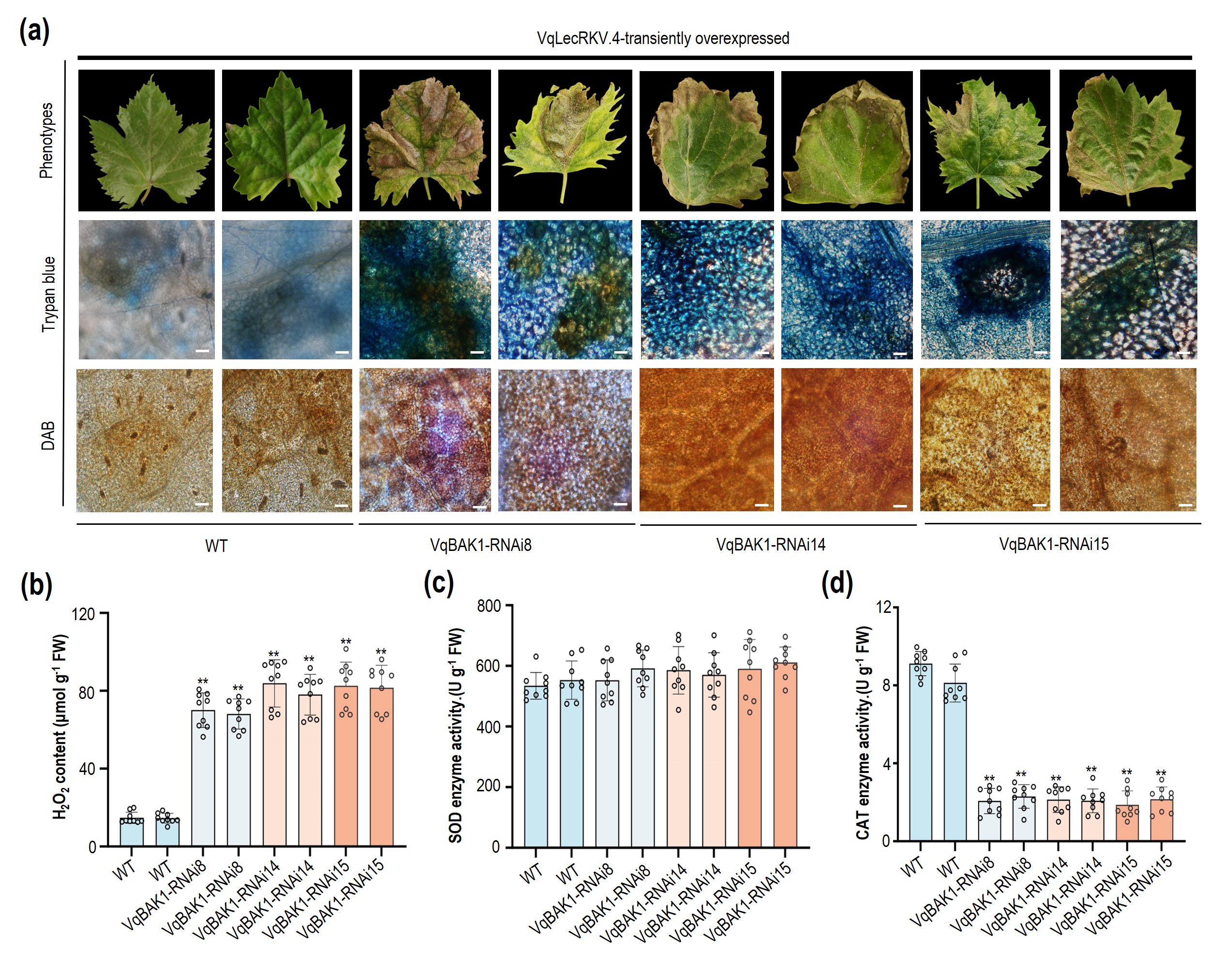


**Figure S17** VqBAK1 controlled cell death in OE-*VqLecRKV.4* grapevine induced by *E. necator* infection. (a) The phenotype (Scale bars = 1 cm), trypan blue (Scale bars = 50 μm) and DAB (Scale bars = 50 μm) staining of leaves inoculated with *E. necator* at 7 dpi following transient transformation of *VqLecRKV.4* overexpression into WT and *VqBAK1* RNAi lines leaves. (b, c, d) Measurement of H_2_O_2_ content, SOD and CAT enzyme activity in leaves inoculated with *E. necator* at 7 dpi after transient transformation of *VqLecRKV.4* overexpression into the leaves of WT and *VqBAK1* RNAi lines. Asterisks indicate a significant difference to *VqBAK1* overexpression lines. Values are the means ± SD of three biological replicates, each with three technical replicates (n = 9).Asterisks indicate statistical significance using a Tukey's multiple comparison test followed by one-way ANOVA (**P* < 0.05, ***P* < 0.01).


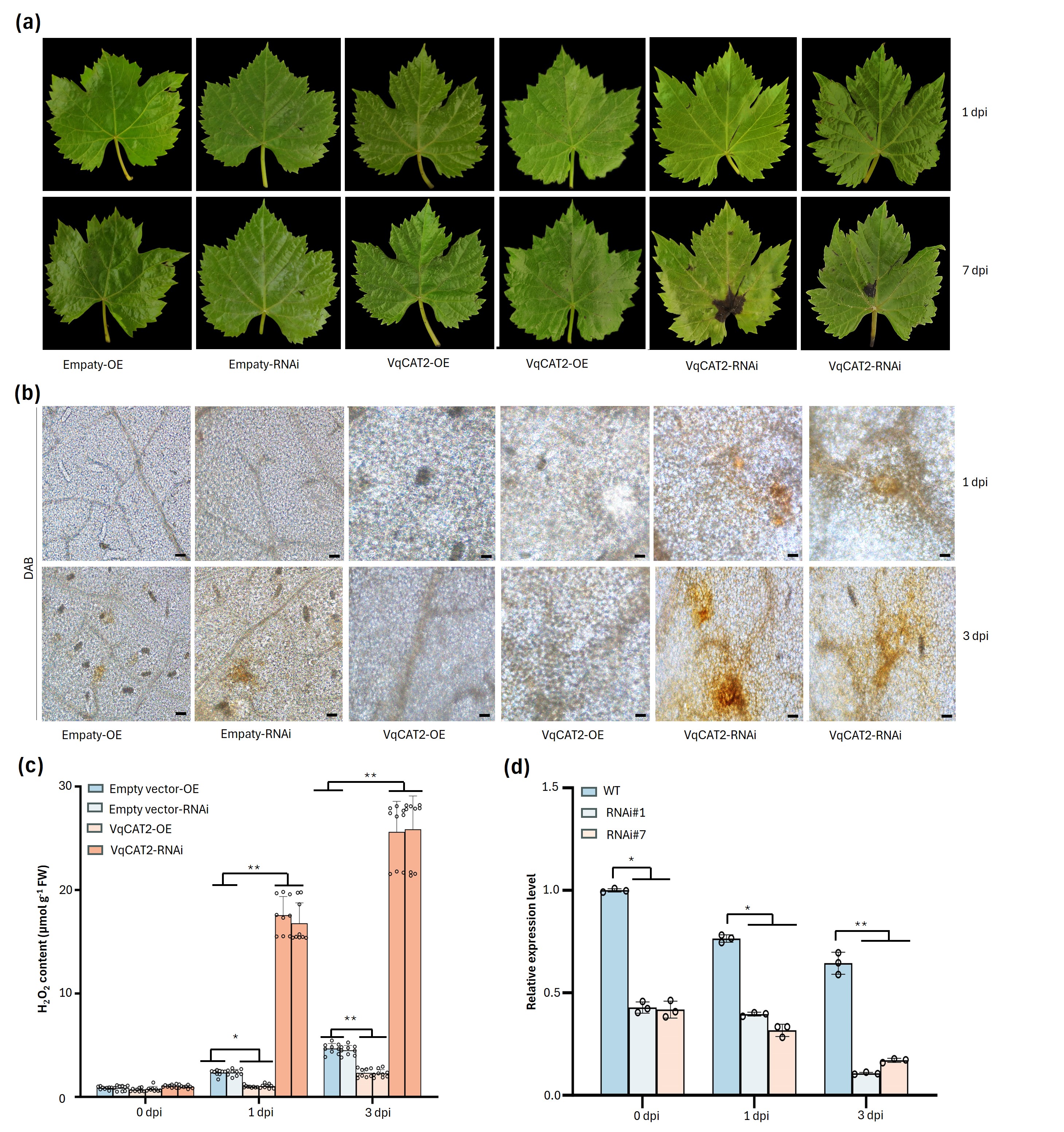


**Figure 18** The functional analysis of *VqCAT2* in response to powdery mildew in *V. vinifera*. (a) The phenotype of Empty vector-OE, Empty vector-RNAi, overexpressing *VqCAT2* and *VqCAT2-*RNAi at 1 and 7 dpi with *E. necator.* Scale bars = 1 cm. (b) 3,3-diaminobenzidine (DAB) staining of H_2_O_2_ production in *E. necator*-infected Empty vector-OE, Empty vector-RNAi, overexpressing *VqCAT2* and *VqCAT2-*RNAi leaves at 1 and 3 dpi. Scale bars = 50 μm. (c) H_2_O_2_ content of Empty vector-OE, Empty vector-RNAi, overexpressing *VqCAT2* and *VqCAT2-*RNAi leaves at 1 and 3 dpi with *E. necator*. Values are the means ± SD of three biological replicates, each with three technical replicates (n = 9). Asterisks indicate statistical significance using a Tukey's multiple comparison test followed by one-way ANOVA (**P* < 0.05, ***P* < 0.01). (d) The expression patterns of *VqCAT2* in WT, RNAi1 and RNAi7 lines after inoculation with *E. necator* at different time points.Values are the means ± SD of three biological replicates (n = 3). Asterisks indicate a significant different groups based on a t test (**P* <0.05, ***P* <0.01).

Table S1 The primers used in this study.

| Primer names | Sequence (5'-3') | Purpose |
| --- | --- | --- |
| VqLecRKI.1-qF1 | CAGATACCAGGGGACTCTTGCAG | qRT-PCR |
| VqLecRKI.1-qR1 | CGGTATCTGGAAGAGAGGGTGAG | qRT-PCR |
| VqLecRKI.6-qF1 | AGTCTTCTTATGCTCAGAAGC | qRT-PCR |
| VqLecRKI.6-qR1 | CTTAATGAATGTCGTCCACCCC | qRT-PCR |
| VqLecRKI.7-qF1 | GCCAGCTTAGCCACAGATAACTGG | qRT-PCR |
| VqLecRKI.7-qR1 | GCTCTCAAATGACCATCCGGCC | qRT-PCR |
| VqLecRKI.8-qF1 | GCCCAAGATGCCTCTTATCCAG | qRT-PCR |
| VqLecRKI.8-qR1 | GTAGAAGCCACATACAAAACC | qRT-PCR |
| VqLecRKI.9-qF1 | GTTCTGAAATCACTTCCATTTC | qRT-PCR |
| VqLecRKI.9-qR1 | GTACGGTGGAGTTCACATATG | qRT-PCR |
| VqLecRKII.1-qF1 | CCCTCTCTGATCCATCTTCTTCC | qRT-PCR |
| VqLecRKII.1-qR1 | CCCTTGCCATGCTTACTGTCCC | qRT-PCR |
| VqLecRKII.4-qF1 | GAATATGACGGCGCATCGATCG | qRT-PCR |
| VqLecRKII.4-qR1 | CGCCGTCGCCGTGTACCAGAAC | qRT-PCR |
| VqLecRKII.5-qF1 | CCAGATGCTGCAGCAGCCTAC | qRT-PCR |
| VqLecRKII.5-qR1 | GGCCTAACTGTCTGATTCAC | qRT-PCR |
| VqLecRKII.6-qF1 | GGGAATTTGAGAATTTATAGC | qRT-PCR |
| VqLecRKII.6-qR1 | CCTACAGGCCGATATCCCAACG | qRT-PCR |
| VqLecRKII.7-qF1 | GTTAATGGAAACCTTGTCGTCC | qRT-PCR |
| VqLecRKII.7-qR1 | GCAAGAAGGGCCCTGGTGCAGG | qRT-PCR |
| VqLecRKIII.1-qF1 | GACACCCACTTCTCCAAATCTC | qRT-PCR |
| VqLecRKIII.1-qR1 | GAAGAATAGTATCGGTCGGAG | qRT-PCR |
| VqLecRKIII.6-qF1 | CTTGTTCTGAAGAACCCACATGG | qRT-PCR |
| VqLecRKIII.6-qR1 | CTAGAACAGCAATTCTGCTACTG | qRT-PCR |
| VqLecRKIII.7-qF1 | GGGTTGCTGTTTCACAAATTTC | qRT-PCR |
| VqLecRKIII.7-qR1 | CAAACAGAACCAGTTCTCCAC | qRT-PCR |
| VqLecRKIII.10-qF1 | GGGCGGGACGCTTCAAGCATTTAC | qRT-PCR |
| VqLecRKIII.10-qR1 | GGTCAATCTTCTTTGGACTCTC | qRT-PCR |
| VqLecRKIV.2-qF1 | GCTTCTATGCTCGATTCTGG | qRT-PCR |
| VqLecRKIV.2-qR1 | GTCCAATAAGGGTCCTCCGAAAC | qRT-PCR |
| VqLecRKIV.4-qF1 | CTGGATTCGGGAAATTTCGTG | qRT-PCR |
| VqLecRKIV.4-qR1 | GCATAATAGGCATATGTTTCC | qRT-PCR |
| VqLecRKIV.5-qF1 | CCGTTCATGGGCTTGTCTTG | qRT-PCR |
| VqLecRKIV.5-qR1 | GCTGCATCTTCAACTGGAAC | qRT-PCR |
| VqLecRKV.3-qF1 | GGAAGAGGAGCATCAGGGTC | qRT-PCR |
| VqLecRKV.3-qR1 | GCCGGACGAGATTTCTGTGA | qRT-PCR |
| VqLecRKV.4-qF1 | CCTTTATCCGCATTCATACCTG | qRT-PCR |
| VqLecRKV.4-qR1 | CTGTCTGCATCCCTTCACTTCA | qRT-PCR |
| VqLecRKV.5-qF1 | GAGTGATGGGGACCAAAGGG | qRT-PCR |
| VqLecRKV.5-qR1 | TTCATCCTCCACCTCTGGCT | qRT-PCR |
| VqLecRKV.9-qF1 | TGACATTACAGCCTCCCAGG | qRT-PCR |
| VqLecRKV.9-qR1 | AGGAGCCGGGTTATCTCCAT | qRT-PCR |
| VqLecRKV.10-qF1 | GAATTGCCCAGCTGCCAAAC | qRT-PCR |
| VqLecRKV.10-qR1 | AGCAGTTGAGTACTCGAAGACA | qRT-PCR |
| VqLecRKVI.5-qF1 | CCTCAATGATTCTTCCGGTG | qRT-PCR |
| VqLecRKVI.5-qR1 | GTGTTAGTGCTAATTTTCATC | qRT-PCR |
| VqLecRKVII.2-qF1 | CAGCTACTCTACTTGATTCTGG | qRT-PCR |
| VqLecRKVII.2-qR1 | CCAGTAGGTGCCCCCTCGGC | qRT-PCR |
| VqLecRKVII.3-qF1 | CTGTGGGCCTTTTGGAATCTG | qRT-PCR |
| VqLecRKVII.3-qR1 | CCCATACTGTACACTTCTCC | qRT-PCR |
| VqLecRKVII.7-qF1 | CCGTGGTTCTTACTGTCAGCAC | qRT-PCR |
| VqLecRKVII.7-qR1 | CTCTACTCTTCCATGATACC | qRT-PCR |
| VqLecRKVII.9-qF1 | GAGACTATCCATTCACAAATCC | qRT-PCR |
| VqLecRKVII.9-qR1 | GTCATATCCAAGTTTCATTCCGG | qRT-PCR |
| VqLecRKVIII.3-qF1 | CACCATAGATGATGACGATG | qRT-PCR |
| VqLecRKVIII.3-qR1 | CACAAAACCTGCCCCCATCC | qRT-PCR |
| VqLecRKIX.4-qF1 | CCACTGACACCATAACTCCTAACC | qRT-PCR |
| VqLecRKIX.4-qR1 | GGTCTCTGTTAAGAACCCATAC | qRT-PCR |
| VqLecRKX.5-qF1 | CGATACTTGGGGATATGGTAC | qRT-PCR |
| VqLecRKX.5-qR1 | CATAGGAAGTTTTCTGGGTC | qRT-PCR |
| VqLecRKX.6-qF1 | CAATTGGGTTTCTTCAGTCC | qRT-PCR |
| VqLecRKX.6-qR1 | GATCCTGCGCAGAACGTGATG | qRT-PCR |
| VqLecRKX.7-qF1 | CACTTCAGCTGGTGGGAGC | qRT-PCR |
| VqLecRKX.7-qR1 | CAAAGAATTCCATTAGTATC | qRT-PCR |
| VqLecRKX.8-qF1 | CCAATAGAGAAAGTCCATTAAC | qRT-PCR |
| VqLecRKX.8-qR1 | CCATCCAAACTTCATGCCTG | qRT-PCR |
| VqLecRKX.12-qF1 | CCAGTTGATTCCAATAATCG | qRT-PCR |
| VqLecRKX.12-qR1 | CATGCCTGGTAGAACAGTATC | qRT-PCR |
| VqActin1-qF1 | GATTCTGGTGATGGTGTGAGT | qRT-PCR |
| VqActin1-qR1 | GACAATTTCCCGTTCAGCAGT | qRT-PCR |
| VvEF1-α | CGCCTGTCAATCTTGGTCAGTAT | qRT-PCR |
| VvEF1-α | AATGGCTATGCCCCTGTTCTG | qRT-PCR |
| VqLecRKV.4-F1 | GGGGTACCATGGCAACTACACTGG | subcellular localization vector |
| VqLecRKV.4-R1 | GCTCTAGATCAAACTGAAAGGG | subcellular localization vector |
| VqLecRKV.4-F2 | GCGTCGACATGGCAACTACACTGG | plant expression vector |
| VqLecRKV.4-R2 | GGGGTACCTCAAACTGAAAGGGTAA | plant expression vector |
| VqLecRKV.4-F3 | ACACAATGAAGTGAAGGGATGC | RNAi vector |
| VqLecRKV.4-R3 | GGTCATTAGTAGAATCAAACCTC | RNAi vector |
| VqLecRKV.4-F4 | CAAAATATCTGCAATGGCCATTACGGCCCAAATTTCTCGAAACTTTAC | pBT3-SUC vector |
| VqLecRKV.4-R4 | CGAATTCCTGCAGATGGCCGAGGCGGCCCCAACTGAAAGGGTAAATGAGG | pBT3-SUC vector |
| VqLecRKV.4-F5 | CCCTCGAGATGGATGCTCAAATTTCTCG | BiFC vector |
| VqLecRKV.4-R5 | GGGGTACCGGACTCATGCTTGTCC | BiFC vector |
| VqLecRKV.4-F6 | GGGGACAAGTTTGTACAAAAAAGCAGGCTGCATGGATGCTCAAATTTCTCG | Split luc vector |
| VqLecRKV.4-R6 | GGGGACCACTTTGTACAAGAAAGCTGGGTCGGACTCATGCTTGTCC | Split luc vector |
| VqLecRKV.4-F7 | GGGTCGACATTTAAATACTAGTATGGCAACTACACTGGC | 1300-mcherry vector |
| VqLecRKV.4-R7 | CCTCCTCGCCCTTGCTCACCATGGTACCAACTGAAAGGGTAAATGAGG | 1300-mcherry vector |
| VqLecRKV.4-F8 | CGGGGGACTCTAGAGGATCCATGCAAATTTCTCGAAACTTTAC | 2300-flag vector |
| VqLecRKV.4-R8 | TGGTCCTTGTAATCGGTACCAACTGAAAGGGTAAATGAGG | 2300-flag vector |
| qPCR-rbohC2-F | GGCAGACGGATGGTTCTGCTGG | qRT-PCR |
| qPCR-rbohC2-R | CTGTGATTCTACCGTCGGCG | qRT-PCR |
| qPCR-EDS1-F | TTGATGTTATCCCTCGTATTATGCTT | qRT-PCR |
| qPCR-EDS1-R | TGAGATCTGGGATTGAAGAAATTG | qRT-PCR |
| qPCR-PR5-F | CTGCAATAACCCATGCACCG | qRT-PCR |
| qPCR-PR5-R | TTGCATGAGCACATTCAGTACAC | qRT-PCR |
| VqLecRKV.4-F9 | CTGTCAAACACTGATAGTTTAAACTGAAGGCGGGAAACG | Turbo ID vector |
| VqLecRKV.4-R9 | GAAGCCAGTGTAGTTGCCATCATGCGGCCGCGGAGCCTGC | Turbo ID vector |
| VqLecRKV.4-F10 | ATGGCAACTACACTGGCTTC | Turbo ID vector |
| VqLecRKV.4-R10 | GGAACCTCCGCCGCTTCCACCGCCTCAACTGAAAGGGTAAATGAGG | Turbo ID vector |
| VqLecRKV.4-F11 | GGAGGCGGTGGAAGCGGCGGAGGTTCCGGCAAGCCCATCCCCAACCC | Turbo ID vector |
| VqLecRKV.4-R11 | GAGGGGGTACCGAGCTGGGGCAATTCCCGATCTAGTAACA | Turbo ID vector |
| VqCu/ZnSOD1-F1 | GTCGGCCGCCTCGGCCTCTCGAGAATTCGATGGTGAAGGCTGTTG | pPR3-N vector |
| VqCu/ZnSOD1-R1 | CTAATTACATGACTCGAGGTCGACTTAGCCTTGCAATCCGATAAC | pPR3-N vector |
| VqCu/ZnSOD1-F2 | TGGCGCGCCACTAGTGGATCCATGGTGAAGGCTGTTG | BiFC vector |
| VqCu/ZnSOD1-R2 | CTCCATCCCGGGAGCGGTACCGCCTTGCAATCCGATAAC | BiFC vector |
| VqCu/ZnSOD1-F3 | ACGGGGGACGAGCTCGGTACCATGGTGAAGGCTGTTG | GFP vector |
| VqCu/ZnSOD1-R3 | GGTGTCGACTCTAGAGGATCCGCCTTGCAATCCGATAAC | GFP vector |
| VqCu/ZnSOD1-F4 | ACGCGTCCCGGGGCGGTACCATGGTGAAGGCTGTTG | Split luc vector |
| VqCu/ZnSOD1-R4 | TACGAACGAAAGCTCTGCAGGCCTTGCAATCCGATAAC | Split luc vector |
| VqCu/ZnSOD1-F5 | GCTGTTCTTAACAGTAATGAGG | qRT-PCR |
| VqCu/ZnSOD1-R5 | CACCAGCATGACGATTCTCG | qRT-PCR |
| NAC2-F | GTCGGCCGCCTCGGCCTCTCGAGGAATTCATGATGAGCGGAGATCAGTTG | pPR3-N vector |
| NAC2-R | CTAATTACATGACTCGAGGACGTCTCAAAATGACTTGTTCGAGAAC | pPR3-N vector |
| SAPK3-F | GTCGGCCGCCTCGGCCTCTCGAGGAATTCATGGAGGAGAGGTACGATG | pPR3-N vector |
| SAPK3-R | CTAATTACATGACTCGAGGACGTCTCACACAGGGGCCATGAAC | pPR3-N vector |
| MEKK1-F | GTCGGCCGCCTCGGCCTCTCGAGGAATTCATGGACTCAAAGCACAAGC | pPR3-N vector |
| MEKK1-R | CTAATTACATGACTCGAGGACGTCTTAAGGTCGTATTCCATTC | pPR3-N vector |
| CML42-F | GTCGGCCGCCTCGGCCTCTCGAGGAATTCATGGAATCAGGTGGAGCCG | pPR3-N vector |
| CML42-R | CTAATTACATGACTCGAGGACGTCTCAAGAGCTCCGAACAACTAC | pPR3-N vector |
| PBS1-F | GTCGGCCGCCTCGGCCTCTCGAGGAATTCATGGATGGGTTGTTTTCCTTGTTTCGA | pPR3-N vector |
| PBS1-R | CTAATTACATGACTCGAGGACGTCTCAACCCGTTAGTGCCATCAAAACTGCCTTGAGCAC | pPR3-N vector |
| VqBAK1-qF1 | GCAGGGTGATTACATTGAGGT | qRT-PCR |
| VqBAK1-qR1 | TGGTTGTACTCTTGGCGGATG | qRT-PCR |
| VqBAK1-F1 | TAAATGCTGTGGAACCGCAGGGTGTGCTGACTA | plant expression vector |
| VqBAK1-R1 | CGGGGATCCTCTAGAGATATCAACCGCCACCTCTCCGCA | plant expression vector |
| VqBAK1-LD-F1 | CCGGAATTCATGGACCCGGGGATCTTC | BD vector |
| VqBAK1-LD-R1 | GCGTCGAC TCATCTCGGACCAGACAATTC | BD vector |
| VqBAK1-F2 | CGGGATCCATGTCCGAAGGTGATGCTTTG | BiFC vector |
| VqBAK1-R2 | CCCTCGAGGGCTCCAGTGGCACTGTTTCC | BiFC vector |
| VqBAK1-F3 | CGGGGGACTCTAGAGGATCCATGGACCCGGGGATCTTCGG | 2300-FLAG vector |
| VqBAK1-R3 | TGGTCCTTGTAATCGGTACCTCTCGGACCAGACAATTC | 2300-FLAG vector |
| VqBAK1-F4 | TCTATTTTATGTAATGGCCATTACGGCCAATTCCGAAGGTGATGC | pBT3-SUC vector |
| VqBAK1-R4 | CGAATTCCTGCAGATGGCCGAGGCGGCCCCTCTCGGACCAGACAATTC | pBT3-SUC vector |
| VqBAK1-F5 | GGGTCGACATTTAAATACTAGTATGGACCCGGGGATCTTCGG | 1300-mcherry vector |
| VqBAK1-R5 | CCTCCTCGCCCTTGCTCACCATGGTACCTCTCGGACCAGACAATTC | 1300-mcherry vector |
| VqBAK1-F5 | CTGTCAAACACTGATAGTTTAAACTGAAGGCGGGAAACG | Turbo ID vector |
| VqBAK1-R5 | CCGAAGATCCCCGGGTCCATCATGCGGCCGCGGAGCCTGC | Turbo ID vector |
| VqBAK1-F5 | ATGGACCCGGGGATCTTCGG | Turbo ID vector |
| VqBAK1-R5 | GGAACCTCCGCCGCTTCCACCGCCTCCTCTCGGACCAGACAATTCAT | Turbo ID vector |
| VqBAK1-F5 | GGAGGCGGTGGAAGCGGCGGAGGTTCCGGCAAGCCCATCCCCAACCC | Turbo ID vector |
| VqBAK1-R5 | GAGGGGGTACCGAGCTGGGGCAATTCCCGATCTAGTAACA | Turbo ID vector |
| VqBAK1-F6 | CTGCGTGAACTACAAGTTGCAACGGA | RNAi vector |
| VqBAK1-R6 | CGGTGGAGTCAATCCAATTAGAG | RNAi vector |
| VqCAT2-F1 | TAAATGCTGTGGAAATGGATCCTTACAAGTATCG | plant expression vector |
| VqCAT2-R1 | CGGGGATCCTCTAGAGATATCATACTTAGGCTTGACATTAAG | plant expression vector |
| VqCAT2-F2 | ACGCGTCCCGGGGCGGTACCATGGATCCTTACAAGTATCG | Split luc vector |
| VqCAT2-R2 | TACGAACGAAAGCTCTGCAGATACTTAGGCTTGACATTAAG | Split luc vector |
| VqCAT2-F3 | TGGCGCGCCACTAGTGGATCCATGGATCCTTACAAGTATCG | BiFC vector |
| VqCAT2-R3 | TCCATCCCGGGAGCGGTACCATACTTAGGCTTGACATTAAG | BiFC vector |
| VqCAT2-F4 | GTCGGCCGCCTCGGCCTCTCGAGAATTCGATGGATCCTTACAAGTATCG | pPR3-N vector |
| VqCAT2-R4 | CTAATTACATGACTCGAGGTCGACTCAATACTTAGGCTTGAC | pPR3-N vector |

Table S2 Characteristics of grapevine G-type LecRK genes.

| Gene ID | Gene ID in Grapedia | Accession no. | Start site | End site | Chr | Full length(bp) | CDS(bp) | ORF(aa) |
| --- | --- | --- | --- | --- | --- | --- | --- | --- |
| LOC100264487 | Vitvi05_01chr01g07890 | XP_002281523.1 | 5800349 | 5803369 | 1 | 3031 | 2367 | 788 |
| LOC100242272 | Vitvi05_01chr04g06770 | XP_002282684.1 | 4950992 | 4955069 | 4 | 4078 | 2448 | 815 |
| LOC100254123 | Vitvi05_01chr04g23280 | XP_002276322.2 | 23630400 | 23632941 | 4 | 2542 | 1899 | 632 |
| LOC100264417 | Vitvi05_01chr04g23310 | XP_002276274.2 | 23679193 | 23681863 | 4 | 2671 | 2334 | 777 |
| LOC100257411 | Vitvi05_01chr04g23530 | XP_002275811.2 | 23919509 | 23922093 | 4 | 2585 | 2379 | 792 |
| LOC100248793 | Vitvi05_01chr04g23640 | XP_002275615.1 | 24044832 | 24047254 | 4 | 2423 | 2307 | 768 |
| LOC100253884 | Vitvi05_01chr04g23650 | XP_002275592.1 | 24054551 | 24057090 | 4 | 2540 | 2319 | 772 |
| LOC100245961 | Vitvi05_01chr05g07200 | XP_002278028.1 | 4966530 | 4968887 | 5 | 2358 | 2358 | 785 |
| LOC100263125 | Vitvi05_01chr05g07240 | XP_002278071.1 | 4993831 | 4996179 | 5 | 2349 | 2349 | 782 |
| LOC100254587 | Vitvi05_01chr05g07280 | XP_002278198.1 | 5040420 | 5042783 | 5 | 2364 | 2364 | 787 |
| LOC100267225 | Vitvi05_01chr06g01040 | XP_002283563.1 | 806232 | 808664 | 6 | 2433 | 2433 | 810 |
| LOC100262073 | Vitvi05_01chr06g02140 | XP_002283233.1 | 1640235 | 1647145 | 6 | 6911 | 2421 | 806 |
| LOC100244923 | Vitvi05_01chr06g02160 | XP_019076118.1 | 1647644 | 1654281 | 6 | 6638 | 2385 | 794 |
| LOC100250062 | Vitvi05_01chr06g02170 | XP_002283213.1 | 1654626 | 1670187 | 6 | 2766 | 2403 | 800 |
| LOC109122813 | Vitvi05_01chr06g02240 | XP_019076212.1 | 1670958 | 1688125 | 6 | 2596 | 2388 | 795 |
| LOC100248357 | Vitvi05_01chr06g04950 | XP_002282125.1 | 4049106 | 4052552 | 6 | 3447 | 2394 | 797 |
| LOC100256499 | Vitvi05_01chr06g16140 | XP_003632253.1 | 17664245 | 17667038 | 6 | 2794 | 2499 | 832 |
| LOC100253318 | Vitvi05_01chr06g21200 | XP_002269411.1 | 22764022 | 22766733 | 6 | 2712 | 2433 | 810 |
| LOC100243307 | Vitvi05_01chr07g14350 | XP_019076653.1 | 14358752 | 14373431 | 7 | 4578 | 2076 | 691 |
| LOC100253540 | Vitvi05_01chr07g14380 | XP_010652593.1 | 14388520 | 14395320 | 7 | 3256 | 2370 | 789 |
| LOC100263838 | Vitvi05_01chr07g14450 | XP_010652595.1 | 14445050 | 14449528 | 7 | 3776 | 2370 | 789 |
| LOC100250063 | Vitvi05_01chr07g14760 | XP_010652590.1 | 14987166 | 14990490 | 7 | 2659 | 2208 | 735 |
| LOC100249399 | Vitvi05_01chr08g14640 | XP_003632709.1 | 15277500 | 15279911 | 8 | 2412 | 2412 | 803 |
| LOC100240889 | Vitvi05_01chr08g14700 | XP_002272276.2 | 15323218 | 15334223 | 8 | 11006 | 2430 | 809 |
| LOC100242625 | Vitvi05_01chr08g14720 | XP_002272467.1 | 15343367 | 15348842 | 8 | 5476 | 2430 | 809 |
| LOC100253673 | Vitvi05_01chr08g23790 | XP_002277767.1 | 21586797 | 21593183 | 8 | 6387 | 2277 | 758 |
| LOC100852792 | Vitvi05_01chr09g05380 | XP_003632791.2 | 4127847 | 4133231 | 9 | 5385 | 2079 | 692 |
| LOC100259444 | Vitvi05_01chr09g08450 | XP_002265931.1 | 6731971 | 6735116 | 9 | 3146 | 2487 | 828 |
| LOC100855046 | Vitvi05_01chr10g06060 | XP_019073964.1 | 4267646 | 4276205 | 10 | 2648 | 2733 | 910 |
| LOC100246472 | Vitvi05_01chr10g06080 | XP_010646910.1 | 4288347 | 4291836 | 10 | 2705 | 2517 | 838 |
| LOC100264960 | Vitvi05_01chr10g06090 | XP_010646911.1 | 4305794 | 4309671 | 10 | 2956 | 2502 | 833 |
| LOC100257760 | Vitvi05_01chr10g06160 | XP_019074082.1 | 4328577 | 4332309 | 10 | 3733 | 2505 | 834 |
| LOC100854728 | Vitvi05_01chr10g06170 | XP_010647032.1 | 4390323 | 4393695 | 10 | 3373 | 2505 | 834 |
| LOC100853235 | Vitvi05_01chr10g06150 | XP_010647107.1 | 4489680 | 4492281 | 10 | 2602 | 2517 | 838 |
| LOC100254695 | Vitvi05_01chr10g06280 | XP_010646735.1 | 4507182 | 4511293 | 10 | 3323 | 2502 | 833 |
| LOC100256534 | Vitvi05_01chr10g06390 | XP_010646880.1 | 4585828 | 4601267 | 10 | 5150 | 2544 | 847 |
| LOC100852997 | Vitvi05_01chr10g06430 | XP_010646876.1 | 4637471 | 4640981 | 10 | 2784 | 2481 | 826 |
| LOC100266824 | Vitvi05_01chr10g06440 | XP_010646877.1 | 4649898 | 4653358 | 10 | 2732 | 2481 | 826 |
| LOC100249655 | Vitvi05_01chr10g06460 | XP_059596180.1 | 4673865 | 4681089 | 10 | 5390 | 2562 | 853 |
| LOC104877369 | Vitvi05_01chr10g06550 | XP_059596184.1 | 4723009 | 4733842 | 10 | 3303 | 2589 | 862 |
| LOC100254772 | Vitvi05_01chr10g06500 | XP_010646861.2 | 24878471 | 24885667 | 10 | 7197 | 2475 | 824 |
| LOC100246449 | Vitvi05_01chr10g06510 | XP_019073936.1 | 24859280 | 24867663 | 10 | 8384 | 2316 | 771 |
| LOC100241174 | Vitvi05_01chr10g06520 | XP_010646862.1 | 24826985 | 24845829 | 10 | 3381 | 2508 | 835 |
| LOC100251437 | Vitvi05_01chr10g06560 | XP_019073933.1 | 24846234 | 24858144 | 10 | 2978 | 2499 | 832 |
| LOC100256692 | Vitvi05_01chr10g25770 | XP_019077972.1 | 26148592 | 26152351 | 10 | 2752 | 2514 | 837 |
| LOC100249150 | Vitvi05_01chr11g06750 | XP_002282203.3 | 4818734 | 4821328 | 11 | 2595 | 2559 | 852 |
| LOC100254081 | Vitvi05_01chr12g01920 | XP_010657163.1 | 1905862 | 1915894 | 12 | 10033 | 2391 | 796 |
| LOC100259197 | Vitvi05_01chr12g01940 | XP_002278212.1 | 1916439 | 1923051 | 12 | 6613 | 2595 | 864 |
| LOC104880834 | Vitvi05_01chr12g02140 | XP_010657074.1 | 2098119 | 2115241 | 12 | 17123 | 2523 | 840 |
| LOC100253956 | Vitvi05_01chr12g02170 | XP_019078894.1 | 2120069 | 2142597 | 12 | 22529 | 2613 | 870 |
| LOC100257381 | Vitvi05_01chr12g02220 | XP_003633280.1 | 2172268 | 2184432 | 12 | 12165 | 2547 | 848 |
| LOC100245340 | Vitvi05_01chr12g02250 | XP_010657072.1 | 2196521 | 2198950 | 12 | 2430 | 2430 | 809 |
| LOC100262615 | Vitvi05_01chr12g02380 | XP_002277451.2 | 2314816 | 2317599 | 12 | 2784 | 2784 | 927 |
| LOC100267773 | Vitvi05_01chr12g02390 | XP_002277429.1 | 2319511 | 2321664 | 12 | 2154 | 2154 | 717 |
| LOC100245458 | Vitvi05_01chr12g02410 | XP_002277406.2 | 2323491 | 2329057 | 12 | 5567 | 2550 | 849 |
| LOC109121483 | Vitvi05_01chr12g04790 | XP_019078851.1 | 4301093 | 4304776 | 12 | 3684 | 2394 | 797 |
| LOC100267794 | Vitvi05_01chr12g04810 | XP_002274435.3 | 4306806 | 4310077 | 12 | 3272 | 2487 | 828 |
| LOC100267196 | Vitvi05_01chr12g09430 | XP_010657377.1 | 7808538 | 7812910 | 12 | 4373 | 6084 | 2027 |
| LOC100267151 | Vitvi05_01chr12g09500 | XP_010657373.1 | 7856363 | 7859726 | 12 | 3364 | 3048 | 1015 |
| LOC100264794 | Vitvi05_01chr12g09600 | XP_019078990.1 | 8001373 | 8007640 | 12 | 6268 | 5220 | 1739 |
| LOC100240913 | Vitvi05_01chr12g09680 | XP_002277219.1 | 8077219 | 8080179 | 12 | 2961 | 2481 | 826 |
| LOC100252934 | Vitvi05_01chr13g27770 | XP_019079675.1 | 29203717 | 29206705 | 13 | 3263 | 2427 | 808 |
| LOC100258065 | Vitvi05_01chr13g27810 | XP_002269019.4 | 29215685 | 29218361 | 13 | 3251 | 2517 | 838 |
| LOC100240945 | Vitvi05_01chr13g27830 | XP_002268928.1 | 29220654 | 29223237 | 13 | 2584 | 2382 | 793 |
| LOC100246038 | Vitvi05_01chr13g27840 | XP_002268890.2 | 29226168 | 29228773 | 13 | 3121 | 2382 | 793 |
| LOC100261425 | Vitvi05_01chr13g27860 | XP_002268770.1 | 29241856 | 29244701 | 13 | 2846 | 2382 | 793 |
| LOC100248272 | Vitvi05_01chr15g18520 | XP_002276095.2 | 18639469 | 18647029 | 15 | 7561 | 2415 | 804 |
| LOC100243904 | Vitvi05_01chr16g17330 | XP_002275726.1 | 18826205 | 18834557 | 16 | 8353 | 2574 | 857 |
| LOC100254036 | Vitvi05_01chr18g10860 | XP_002283127.1 | 8135122 | 8137527 | 18 | 2406 | 2406 | 801 |
| LOC100247193 | Vitvi05_01chr18g10870 | XP_002283102.1 | 8151295 | 8153837 | 18 | 2543 | 2406 | 801 |
| LOC100257449 | Vitvi05_01chr18g10900 | XP_002283088.2 | 8166275 | 8168677 | 18 | 2403 | 2403 | 800 |
| LOC100245415 | Vitvi05_01chr18g10950 | XP_002283069.1 | 8190899 | 8193301 | 18 | 2403 | 2403 | 800 |
| LOC100260797 | Vitvi05_01chr18g10970 | XP_003634437.1 | 8215284 | 8218356 | 18 | 3073 | 2379 | 792 |
| LOC100243693 | Vitvi05_01chr18g10980 | XP_002283040.3 | 8220838 | 8227069 | 18 | 6232 | 2418 | 805 |
| LOC100247883 | Vitvi05_01chr18g19070 | XP_002264274.2 | 14207946 | 14210423 | 18 | 2478 | 2448 | 815 |
| LOC100252926 | Vitvi05_01chr18g20400 | XP_002267635.1 | 17424571 | 17428021 | 18 | 3451 | 2550 | 849 |
| LOC100253752 | Vitvi05_01chr19g03590 | XP_002282446.1 | 2699751 | 2702129 | 19 | 2379 | 2379 | 792 |
| LOC100246936 | Vitvi05_01chr19g05670 | XP_010644387.1 | 4263881 | 4267453 | 19 | 2578 | 2499 | 832 |
| LOC100260532 | Vitvi05_01chr19g05740 | XP_003634654.2 | 4307994 | 4320768 | 19 | 3846 | 2502 | 833 |
| LOC100252174 | Vitvi05_01chr19g05780 | XP_019072716.1 | 4404335 | 4407077 | 19 | 2743 | 2550 | 849 |
| LOC100255581 | Vitvi05_01chr19g05820 | XP_019072976.1 | 4446405 | 4450067 | 19 | 2533 | 2502 | 833 |
| LOC100262419 | Vitvi05_01chr19g05830 | XP_059591522.1 | 4398582 | 4400861 | 19 | 6335 | 1704 | 567 |
| LOC100265866 | Vitvi05_01chr19g05900 | XP_019072991.1 | 4507989 | 4510361 | 19 | 2373 | 2346 | 781 |
| LOC100258925 | Vitvi05_01chr19g05940 | XP_019072520.1 | 4537166 | 4555213 | 19 | 18048 | 2484 | 827 |
| LOC100246941 | Vitvi05_01chr19g05960 | XP_019072827.1 | 4555814 | 4572521 | 19 | 16708 | 2448 | 815 |
| LOC100267579 | Vitvi05_01chr19g06100 | XP_010644287.1 | 4668721 | 4693768 | 19 | 25048 | 2457 | 818 |
| LOC100245275 | Vitvi05_01chr19g06110 | XP_010644290.1 | 4697709 | 4701879 | 19 | 2930 | 2460 | 819 |
| LOC100243545 | Vitvi05_01chr19g06160 | XP_019072560.1 | 4743383 | 4749486 | 19 | 6104 | 5232 | 1743 |
| LOC100260545 | Vitvi05_01chr19g06280 | XP_010644306.1 | 4851075 | 4855125 | 19 | 4051 | 2457 | 818 |
| LOC100262263 | Vitvi05_01chr19g06290 | XP_010644304.1 | 4866197 | 4869047 | 19 | 2851 | 2460 | 819 |
| LOC104877534 | Vitvi05_01chr19g06310 | XP_010644298.1 | 4902349 | 4906739 | 19 | 8673 | 2445 | 814 |
| LOC100247061 | Vitvi05_01chr19g06340 | XP_010644302.1 | 4892173 | 4925135 | 19 | 39469 | 2322 | 773 |
| LOC100262430 | Vitvi05_01chr19g06370 | XP_019072562.1 | 4943927 | 4947171 | 19 | 3245 | 4980 | 1659 |
| LOC104878636 | Vitvi05_01chr19g05980 | XP_010647533.1 | 21436105 | 21464645 | 19 | 3523 | 2475 | 824 |
| Abbreviations: CDS, coding sequence; ORF, open reading frame; Chr, Chromosome numbers. | | | | | | | | |

Table S3 Classification and proposed nomenclature of grapevine G-type LecRKs.

| **Clade** | **Gene name** | **Gene ID** |
| --- | --- | --- |
| LecRK-I | *VvLecRKI.1* | LOC100242272 |
|  | *VvLecRKI.2* | LOC100256499 |
|  | *VvLecRKI.3* | LOC100259444 |
|  | *VvLecRKI.4* | LOC100249150 |
|  | *VvLecRKI.5* | LOC100254081 |
|  | *VvLecRKI.6* | LOC100259197 |
|  | *VvLecRKI.7* | LOC104880834 |
|  | *VvLecRKI.8* | LOC100253956 |
|  | *VvLecRKI.9* | LOC100257381 |
|  | *VvLecRKI.10* | LOC100245340 |
|  | *VvLecRKI.11* | LOC100262615 |
|  | *VvLecRKI.12* | LOC100267773 |
|  | *VvLecRKI.13* | LOC100245458 |
| LecRK-II | *VvLecRKII.1* | LOC109121483 |
|  | *VvLecRKII.2* | LOC100267794 |
|  | *VvLecRKII.3* | LOC100240913 |
|  | *VvLecRKII.4* | LOC100248272 |
|  | *VvLecRKII.5* | LOC100243904 |
|  | *VvLecRKII.6* | LOC100252926 |
|  | *VvLecRKII.7* | LOC100253752 |
| LecRK-III | *VvLecRKIII.1* | LOC100264487 |
|  | *VvLecRKIII.2* | LOC100267225 |
|  | *VvLecRKIII.3* | LOC100248357 |
|  | *VvLecRKIII.4* | LOC100249399 |
|  | *VvLecRKIII.5* | LOC100240889 |
|  | *VvLecRKIII.6* | LOC100242625 |
|  | *VvLecRKIII.7* | LOC100253673 |
|  | *VvLecRKIII.8* | LOC100254036 |
|  | *VvLecRKIII.9* | LOC100247193 |
|  | *VvLecRKIII.10* | LOC100257449 |
|  | *VvLecRKIII.11* | LOC100245415 |
|  | *VvLecRKIII.12* | LOC100260797 |
|  | *VvLecRKIII.13* | LOC100243693 |
| LecRK-IV | *VvLecRKIV.1* | LOC100254123 |
|  | *VvLecRKIV.2* | LOC100264417 |
|  | *VvLecRKIV.3* | LOC100257411 |
|  | *VvLecRKIV.4* | LOC100248793 |
|  | *VvLecRKIV.5* | LOC100253884 |
|  | *VvLecRKIV.6* | LOC100247883 |
| LecRK-V | *VvLecRKV.1* | LOC100245961 |
|  | *VvLecRKV.2* | LOC100263125 |
|  | *VvLecRKV.3* | LOC100254587 |
|  | *VvLecRKV.4* | LOC100262073 |
|  | *VvLecRKV.5* | LOC100244923 |
|  | *VvLecRKV.6* | LOC100250062 |
|  | *VvLecRKV.7* | LOC109122813 |
|  | *VvLecRKV.8* | LOC100253318 |
|  | *VvLecRKV.9* | LOC100261425 |
|  | *VvLecRKV.10* | LOC100246038 |
|  | *VvLecRKV.11* | LOC100240945 |
|  | *VvLecRKV.12* | LOC100258065 |
|  | *VvLecRKV.13* | LOC100252934 |
| LecRK-VI | *VvLecRKVI.1* | LOC100241174 |
|  | *VvLecRKVI.2* | LOC100251437 |
|  | *VvLecRKVI.3* | LOC100246449 |
|  | *VvLecRKVI.4* | LOC100254772 |
|  | *VvLecRKVI.5* | LOC100266824 |
|  | *VvLecRKVI.6* | LOC100249655 |
|  | *VvLecRKVI.7* | LOC100263919 |
|  | *VvLecRKVI.8* | LOC100852997 |
| LecRK-VII | *VvLecRKVII.1* | LOC100250063 |
|  | *VvLecRKVII.2* | LOC100243307 |
|  | *VvLecRKVII.3* | LOC100256692 |
|  | *VvLecRKVII.4* | LOC100267196 |
|  | *VvLecRKVII.5* | LOC100267151 |
|  | *VvLecRKVII.6* | LOC100264794 |
|  | *VvLecRKVII.7* | LOC100246936 |
|  | *VvLecRKVII.8* | LOC100260532 |
|  | *VvLecRKVII.9* | LOC100252174 |
| LecRK-VIII | *VvLecRKVIII.1* | LOC100263838 |
|  | *VvLecRKVIII.2* | LOC100253540 |
|  | *VvLecRKVIII.3* | LOC100852792 |
| LecRK-IX | *VvLecRKIX.1* | LOC100262419 |
|  | *VvLecRKIX.2* | LOC100255581 |
|  | *VvLecRKIX.3* | LOC100254695 |
|  | *VvLecRKIX.4* | LOC100246472 |
|  | *VvLecRKIX.5* | LOC100264960 |
|  | *VvLecRKIX.6* | LOC100855046 |
|  | *VvLecRKIX.7* | LOC100854728 |
|  | *VvLecRKIX.8* | LOC100853235 |
|  | *VvLecRKIX.9* | LOC100257760 |
| LecRK-X | *VvLecRKX.1* | LOC100265866 |
|  | *VvLecRKX.2* | LOC100258925 |
|  | *VvLecRKX.3* | LOC100246941 |
|  | *VvLecRKX.4* | LOC100267579 |
|  | *VvLecRKX.5* | LOC100243545 |
|  | *VvLecRKX.6* | LOC100245275 |
|  | *VvLecRKX.7* | LOC100260545 |
|  | *VvLecRKX.8* | LOC100262263 |
|  | *VvLecRKX.9* | LOC100247061 |
|  | *VvLecRKX.10* | LOC104877534 |
|  | *VvLecRKX.11* | LOC100262430 |
|  | *VvLecRKX.12* | LOC104878636 |
|  | *VvLecRKX.13* | LOC100256534 |

Table S4 Domains of grapevine G-type LecRK proteins.

| Protein Name | SLG | EGF | PAN | TM | Signal peptide | S_TKc |
| --- | --- | --- | --- | --- | --- | --- |
| VvLecRKI.1 | – | – | * | * | * | * |
| VvLecRKI.2 | – | – | * | * | * | * |
| VvLecRKI.3 | – | – | * | * | * | * |
| VvLecRKI.4 | – | – | * | * | – | * |
| VvLecRKI.5 | – | – | * | * | * | * |
| VvLecRKI.6 | – | – | – | * | * | * |
| VvLecRKI.7 | – | – | * | * | * | * |
| VvLecRKI.8 | – | – | – | * | * | * |
| VvLecRKI.9 | – | – | – | * | * | * |
| VvLecRKI.10 | – | – | * | * | – | * |
| VvLecRKI.11 | – | – | * | * | * | * |
| VvLecRKI.12 | – | – | * | * | * | * |
| VvLecRKI.13 | – | – | – | * | * | * |
| VvLecRKII.1 | * | – | * | * | * | * |
| VvLecRKII.2 | * | – | * | * | – | * |
| VvLecRKII.3 | * | – | * | * | * | * |
| VvLecRKII.4 | * | – | * | * | * | * |
| VvLecRKII.5 | * | * | – | * | * | * |
| VvLecRKII.6 | * | * | – | * | * | * |
| VvLecRKII.7 | * | – | * | * | – | * |
| VvLecRKIII.1 | * | * | * | * | * | * |
| VvLecRKIII.2 | * | – | * | * | – | * |
| VvLecRKIII.3 | * | * | * | * | * | * |
| VvLecRKIII.4 | * | * | * | * | * | * |
| VvLecRKIII.5 | * | * | * | * | * | * |
| VvLecRKIII.6 | * | – | * | * | * | * |
| VvLecRKIII.7 | * | – | * | * | * | * |
| VvLecRKIII.8 | * | * | * | * | * | * |
| VvLecRKIII.9 | * | * | * | * | * | * |
| VvLecRKIII.10 | * | * | * | * | * | * |
| VvLecRKIII.11 | * | * | * | * | * | * |
| VvLecRKIII.12 | * | * | * | * | * | * |
| VvLecRKIII.13 | * | – | * | * | * | * |
| VvLecRKIV.1 | * | * | * | * | * | * |
| VvLecRKIV.2 | * | * | * | * | – | * |
| VvLecRKIV.3 | * | – | * | * | * | * |
| VvLecRKIV.4 | – | * | * | * | * | * |
| VvLecRKIV.5 | * | * | – | * | * | * |
| VvLecRKIV.6 | * | * | – | * | – | * |
| VvLecRKV.1 | * | * | – | * | * | * |
| VvLecRKV.2 | * | * | – | * | * | * |
| VvLecRKV.3 | * | * | – | * | * | * |
| VvLecRKV.4 | – | * | * | * | * | * |
| VvLecRKV.5 | – | * | * | * | * | * |
| VvLecRKV.6 | – | – | * | * | * | * |
| VvLecRKV.7 | – | – | * | * | * | * |
| VvLecRKV.8 | – | * | – | * | * | * |
| VvLecRKV.9 | – | – | – | * | * | * |
| VvLecRKV.10 | – | – | – | * | * | * |
| VvLecRKV.11 | – | – | – | * | * | * |
| VvLecRKV.12 | – | – | – | * | – | * |
| VvLecRKV.13 | * | – | – | * | * | * |
| VvLecRKVI.1 | * | * | * | * | * | * |
| VvLecRKVI.2 | * | * | * | * | * | * |
| VvLecRKVI.3 | * | * | * | * | – | * |
| VvLecRKVI.4 | * | * | * | * | – | * |
| VvLecRKVI.5 | * | * | * | * | * | * |
| VvLecRKVI.6 | * | * | * | * | – | * |
| VvLecRKVI.7 | * | * | * | * | – | * |
| VvLecRKVI.8 | * | * | * | * | * | * |
| VvLecRKVII.1 | – | – | * | * | – | * |
| VvLecRKVII.2 | – | – | * | * | * | * |
| VvLecRKVII.3 | * | * | * | * | * | * |
| VvLecRKVII.4 | * | – | * | * | – | * |
| VvLecRKVII.5 | * | – | * | * | * | * |
| VvLecRKVII.6 | * | * | * | * | – | * |
| VvLecRKVII.7 | * | – | * | * | * | * |
| VvLecRKVII.8 | * | – | * | * | * | * |
| VvLecRKVII.9 | * | – | * | * | – | * |
| VvLecRKVIII.1 | – | – | * | * | * | * |
| VvLecRKVIII.2 | – | – | * | * | * | * |
| VvLecRKVIII.3 | – | – | * | * | * | * |
| VvLecRKIX.1 | * | – | * | * | * | * |
| VvLecRKIX.2 | * | – | * | * | * | * |
| VvLecRKIX.3 | * | * | * | * | * | * |
| VvLecRKIX.4 | * | – | * | * | * | * |
| VvLecRKIX.5 | * | * | * | * | * | * |
| VvLecRKIX.6 | * | – | * | * | * | * |
| VvLecRKIX.7 | * | – | * | * | * | * |
| VvLecRKIX.8 | * | * | * | * | * | * |
| VvLecRKIX.9 | * | * | * | * | * | * |
| VvLecRKX.1 | * | – | * | * | – | * |
| VvLecRKX.2 | * | – | * | * | * | * |
| VvLecRKX.3 | * | * | * | * | – | * |
| VvLecRKX.4 | * | * | * | * | * | * |
| VvLecRKX.5 | * | * | * | * | * | * |
| VvLecRKX.6 | * | * | * | * | * | * |
| VvLecRKX.7 | * | * | * | * | * | * |
| VvLecRKX.8 | * | – | * | * | – | * |
| VvLecRKX.9 | * | – | * | * | – | * |
| VvLecRKX.10 | * | – | * | * | * | * |
| VvLecRKX.11 | * | – | * | * | – | * |
| VvLecRKX.12 | * | * | * | * | * | * |
| VvLecRKX.13 | * | * | * | * | * | * |
| Abbreviations: SLG, S-locus glycoprotein; EGF, epidermal growth factor; PAN, Apple-like domains present in plasminogen; TM, transmembrane; S_TKc, Serine/Threonine protein kinases. | | | | | | |
| "*" denotes presence and “–” denotes absence of a domain | | | | | | |

Table S5 The information of other proteins.

| Gene names | Gene ID | Protein names |
| --- | --- | --- |
| VqCLM42 | LOC100251912 | Calcium-binding protein CML42 |
| VqPBS1 | LOC100254113 | Protein kinase domain-containing protein |
| VqMEKK1 | LOC100241228 | Mitogen-activated protein kinase kinase kinase |
| VqSAPK3 | LOC100243594 | Non-specific serine/threonine protein kinase |
| VqNAC2 | LOC100261123 | NAC domain-containing protein |

Table S6 The information of phosphoprotemoics oxidoreductases.

| Gene names | Gene ID | Protein names |
| --- | --- | --- |
| VqCu/ZnSOD1 | LOC100241809 | superoxide dismutase;Superoxide dismutase [Cu-Zn] |
| VqCu/ZnSOD2 | LOC100260705 | superoxide dismutase [Cu-Zn] 2 |
| Vq2Cys-Prx | LOC100259748 | thioredoxin-dependent peroxiredoxin |
| VqPrx Q | LOC100250698 | Peroxiredoxin Q |
| VqPrx4 | LOC104882489 | peroxidase |
| VqPrx20 | LOC100260742 | Class III peroxidase |
| VqMDHAR | LOC100258894 | monodehydroascorbate reductase |
| VqGPX6 | LOC100254672 | Glutathione peroxidase |
| VqGPX8 | LOC100260742 | Glutathione peroxidase |
| VqAPX3 | LOC100246866 | L-ascorbate peroxidase |
| VqAPX6 | LOC100254009 | L-ascorbate peroxidase |
| VqCAT1 | LOC100853165 | Catalase |
| VqCAT2 | LOC100232861 | Catalase |
| VqCAT3 | LOC100244516 | Catalase |

**Sequences**

>VqLecRK ORF

ATGGCAACTACACTGGCTTCTCAGTCTCTAGGCCATTGTCTTCTGCCTCTGCTGCTGCTGCTTCTGCTTCTGCTACTGCTGCTGTTAGTTTCGAGTGATGCTCAAATTTCTCGAAACTTTACTTCGGGCTCAACCCTCATTGCGAGAGACAACAATTCCTTTTTGGCGTCCCCGAATGGTGATTTCGCTTTTGGATTCCAACAGGTCGGAAGTGGAGGCTTCTTGCTAGCTATTTGGTTCAACAAAGTACCTGAAAGGACCGTAGTTTGGTCAGCCAACCGCGATAGTCTGGTGCAAACTGGATCCAGAGTTCAACTTACCACTGATGGAGAATTCATGCTCAATGACCCTAAAGGTAAACAGATGTGGAAGGCTGATCTGAACAGTACTGAAGTTGTCTATGCAGCCATGCTGGACACTGGAAACTTTGTGCTTGCAGGCCACAATTCCACCTATCTGTGGCAGAGCTTCAATCATTCAACAGACACAATATTACCCACCCAGATACTGAATCAAGGCAGCAAACTTGTTGCTCGTTTCTCGGAGGTGAATTACTCAAGTGGAAGATTCATGCTTATATTGCAAACTGACGGGAATCTTGTGCTTTACACCACTGATTTCCCAATGGATTCTGCAAATTCTGCATATTGGGCAACTGCCACTGTAGGCAGCGGGTTTCAGGTGATCTACAATGAGTCTGGTGACATATACCTCATAGGGAACAACAGGAGAAAACTCAGTGATGTACTGTCAAATAAAGAACCAACAGGAGAATTCTATCAGAGAGCAATTCTTGAATATGATGGAGTTTTCAGACAGTATGTCCACCCGAAGTCTGCTGGATCAGGTGCTCCTATGGCCTGGTCCCCTTTATCCGCATTCATACCTGAAAATATCTGCACAAATATTACCGCAAGTACAGGCAGTGGAGCTTGCGGGTTCAACAGCTACTGCACACTAGGAGATCATCAGAGACCTATTTGCAAGTGCCCTCCTGGTTACACCTTTTTGGATCCACACAATGAAGTGAAGGGATGCAGACAGGACTTTTATCCAGAGATTTGTGATGAAGGATCTCATGAAACAGGTAGGTTTGATTTTGAAAGGATGACAAACGTGGATTGGCCAATGTCAGATTATGACCGATTTCAACTATTTACTGAGGATGATTGCAGAAAAGCTTGCTTGGAAGATTGTTTTTGTGCGGTTGCAATTTTCAGAGATGGGGATTGTTGGAAGAAGAAAATCCCTCTTTCAAATGGGAGGTTTGAGTCTACTAATGACCGAATAGCTCTCATTAAAGTAGAGAAGAAGAATTCTTCTTTCCCACATGGAGGTGAAGGTTTTAAGGACAAGCATGAGTCCATTCTGATCCTTGCTGGATCAGTGCTGCTAGGCAGCTCGGTGCTTCTGAATGTTCTTCTCCTCCTAGCAACTGCTACATTCATCCTCCGCTTGTATTGCAGAAAACCAGCGATCATTGAATCACAACAAGTCATGGTGGGAAGAAACCTACAAAGTTTCACTTACCATGAGCTGGAGGAGGCCACCAATGGATTCAAGGATGAACTAGGAAAGGGGGCTTTTGGAACAGTCTACAAAGGGAGCTGCAATGGAAATCTGGTTGCAGTAAAAAAGTTAGAAAGGATGGTGAAAGAAGGAGAGCGGGAATTTGAAACAGAAGTGAGTGCGATCGTCAGGACAAATCACAAGAATCTAGTCCAACTTCTGGGGTTCTGCAATGAGGGGCTACACCGGCTTCTAGTATACGAGTTCATGAGCAATGGCTCCTTAGCAACCTTCCTATTTGGAAGTTCAAGACCTAAATGGCACCAAAGAATACAGATTATCCTAGGAACTGCAAAAGGCCTTCTGTACTTACATGAAGAGTGCAGCATCCAGACCATACATTGTGACATCAAGCCTCAAAACATTCTCCTGGATGACTCCTTAACAGCAAGGATATCAGACTTTGGATTGGCTAAGTTTCTGAAAACAGATCAGACGCGAACCATGACTGGAATCAGGGGAACCAAAGGTTATGTTGCCCCTGAGTGGTTCAAGACAGTGCCCATTACAGTTAAGGTTGATGTTTACAGCTTTGGAATTGTGTTGCTTGAGCTCATATTCTGCAGGAAGAATTTTGAAGTGGAGGCTGAGGATAAAAGTCCAGTTGTATTGGCTGAATTGGCATACTATTGCTACAAAGAAGGGAAACTGGATATGCTACTGGACAATGATGAAGAGGCACTGGAAGACATGGAGAGGCTGGAGAAGTTTGTGATGATAGCATTTTGGTGCATTCAGGATGATCCACATCAAAGGCCTGGAATGAAGAAAGTCACTCAGATGCTTGAAGGAGCCATTGAAGTTTCTTCTCCTCCAGACTCATCCTCATTTACCCTTTCAGTTTGA

>VqBAK1 ORF

ATGGACCCGGGGATCTTCGGTTCGGTTTTTGTTTCCTTGATTATAGTATTCTCCGCGTTTCTGAGGGTCTCTGGTAATTCCGAAGGTGATGCTTTGAATGCGTTGAAGTCAAATTTAGCTGATCCTAACAATGTTTTGCAAAGTTGGGATGCTACCCTTGTCAATCCTTGCACATGGTTTCATGTTACATGCAACAGTGACAATAGTGTTACAAGAGTTGATCTTGGAAATGCAAATTTATCCGGTCAACTGGTTTCACAGCTTGGTCAGCTTACAAATTTGCAATATCTGGAACTTTATAGTAATAACATAAGTGGCAAAATACCAGAGGAGCTTGGGAATTTGACAAACTTGGTGAGCTTGGATCTTTACATGAACAAGTTAAGTGGTCCAATTCCGACGACGTTGGGCAAGCTTGCAAAACTACGTTTCCTGCGGCTTAACAACAACACGTTGACAGGAACTATTCCAAGATCTTTAACTACTGTTATGACACTGCAAGTCCTGGATCTTTCGAACAATCAGCTAACAGGAGATATACCAGTTGACGGCTCATTTTCATTATTTACTCCTATCAGTTTTAACAATAATAGACTAAACCCACTTCCAGTTTCTCCACCACCACCAATTTCTCCTACACTAACAGCTTCTTCAGGAAACAGTGCCACTGGAGCCATTGCTGGAGGAGTTGCTGCTGGTGCTGCACTTCTGTTTGCTGCCCCTGCAATCGTACTTGCCTGGTGGCGACGAAGGAAACCACAGGAGCACTTTTTTGATGTACCTGCTGAAGAGGATCCAGAAGTTCATTTGGGGCAGCTTAAAAGGTTTTCTCTGCGTGAACTACAAGTTGCAACGGATAATTTTAGTAACAAACACATTCTGGGTAGAGGTGGATTTGGTAAAGTTTACAAAGGACGTTTAACTGATGGTTCTCTAGTGGCAGTAAAAAGACTGAAAGAGGAGCGTACTCAGGGTGGGGAACTGCAATTTCAGACAGAAGTCGAAATGATCAGCATGGCTGTGCACCGGAATCTACTTCGTCTACGTGGCTTTTGCATGACACCAACAGAACGGTTGCTTGTCTATCCCTTTATGGTTAATGGAAGTGTTGCATCATGTTTGAGAGAGCGAGTTGATGGACAGTCTCCCCTTAATTGGCCAATAAGGAAACAAATTGCTTTAGGATCAGCAAGGGGGCTTGCTTATTTGCATGATCATTGTGACCCTAAGATTATTCATCGTGATGTGAAGGCTGCAAACATATTGTTGGATGAAGAGTTCGAAGCAGTAGTAGGAGACTTTGGGCTGGCTAAACTCATGGACTACAAGGATACTCATGTTACCACTGCTGTACGTGGCACAATTGGGCATATTGCTCCGGAGTACCTCTCCACTGGGAAGTCTTCAGAGAAGACTGATGTTTTTGGATATGGAGTAATGCTTCTTGAGCTTATCACTGGACAGAGGGCTTTTGATCTTGCGCGGCTTGCCAATGATGATGATGTTATGTTACTTGATTGGGTAAAAGGACTACTGAAAGATAAGAAGTTGGAGACACTGGTTGATGCCGATCTGCAGGGTGATTACATTGAGGTAGAAGTAGAGGAGCTAATTCGGGTGGCTCTCCTCTGCACAGATGGTGCTGCTGCACAACGACCCAAAATGTCAGAGGTGGTCAGAATGCTTGAAGGTGATGGTTTGGCCGAGAGATGGGAACAATGGGAGAAGGATGATATCATCCGCCAAGAGTACAACCATATCCCCCACCCAGACTCTAATTGGATTGACTCCACCGCAGGCCTCCGCCCTGATGAATTGTCTGGTCCGAGATGA

>VqCu/ZnSOD1

ATGGTGAAGGCTGTTGCCGTTCTTAACAGTAATGAGGGTGTTTGTGGAACTATCTACTTCGCTGAAGAAGGAGATGGTTCGACTACAGTGACTGGGAGTCTTTCTGGCCTTAAGCCTGGACTTCATGGCTTCCATGTGCATGCCCTTGGGGACACAACAAATGGTTGCATGTCAACTGGACCTCATTTCAATCCTGCTGGAAAAGAGCATGGTGCTCCTGAAGACGAGAATCGTCATGCTGGTGATTTAGGAAATGTCATTGTTGGTGAGGATGGTACTGTTAATTTCAAAATTGTTGACAAGCAGATTCCTCTCACTGGATCAAACTCCATTGTTGGAAGGGCTGTTGTTGTCCATGCTGATCCTGATGATCTTGGAAACGGGGGACATGAGCTCAGCAAAAGCACAGGAAATGCTGGTGGCAGAGTAGCCTGTGGAGTTATCGGATTGCAAGGCTAA

>VqCAT2

ATGGATCCTTACAAGTATCGTCCTTCAAGCGCCTATAATTCTCCTTACTTCACCACAAATGCTGGTGCGCCTGTTTACAACAATGACTCGTCGCTCACCGTTGGATCAAGAGGACCAGTTCTTCTTGAGGATTATCATTTGGTGGAGAAGCTTGCAAATTTTCAAAGAGAACGCATTCCAGAACGTGTGGTCCATGCTAGAGGAGCTAGTGCTAAAGGGTTTTTTGAGGTCACTCACGATGTCTCTGACCTCACATGTGCTGATTTCCTTCGTGCCCCTGGAGTGCAGACTCCAGTTATTGTTAGGTTTTCCACTGTTATCCACGAGCGTGGCAGCCCTGAGACCATAAGGGACCCTCGAGGTTTTGCTGTGAAGTTCTATACTAGAGAGGGCAATTTTGATATGGTGGGAAACAATTTCCCAGTCTTCTTTATTCGTGATGCAATGAAGTTCCCTGATGTGATCCATGCTTTTAAACCCAATCCGAAGTCCCATATCCAGGAGTATTGGAGAGTTGTTGACTTCTTGTCCTACCATCCAGAAAGCTTGAGCACATTTGCCTTCCTCTTCGATGATGTGGGTGTCCCACAGGACTATAGGCACATGGAAGGCTTTGGTGTTCACACCTTCACTCTAATCAACAAGGCTGGGAAGGCAAATTATGTGAAGTTCCACTGGAAACCCACGTGTGGAGTCAAGTGTTTGTTGGAGGATGAAGCCATAAGAGTAGGAGGAACTAACCACAGTCATGCTACTCAGGATCTCTATGACTCGATCAAGGCTGGAAACTATCCTGAGTGGAAGCTTTTCATCCAGACGATGGATCCTGAACATGAAGACAAGTTTGACTTTGATCCACTTGATGTTACCAAGACTTGGCCTGAGGATATCTTGCCCTTGCAGCCAGTGGGCCGGTTGGTATTAAACAAGAATATAGATAATTTCTTTGCTGAGAATGAGATGCTTGCCTTCAACCCAGCTCTTGTTGTACCTGGTATATATTACACAAATGATAAAATGCTCCAAGGTAGAATCTTTGCCTATTCTGATACTCAGAGGCACCGCCTTGGACCGAACTATCTGCAGCTTCCAGTAAATGCACCCAAGTGTGCTTTTCACAACAATCACCATGACGGTTCCATGAATTTCATGCACAGGGATGAGGAGGTCGACTACTTCCCTTCAAGGTTTGATCCTGTTCGTCATGCGGAGACATTCCCTATTCCTTCTACTATTTGCCATGGCAAGCGTGAGAAGGTCGTCATTGAGAAGGAGAACAATTTCAAGCAGCCTGGTGAGAGATACCGTTCATTTGCACCGGACAGGCAAGAGCGTTTCATCAACAGATGGGTTGAGACCTTGTCTGATCCAAGGGTCACCTATGAGATCCGCAGTATCTGGATTTCATATTGGTCTCAGGCTGACAGGTCTCTGGGTCAGAAGCTTGCTTCTCGTCTTAATGTCAAGCCTAAGTATTGA
